# Impact of switching between reference biologics and biosimilars of tumour necrosis factor inhibitors for rheumatoid arthritis: a systematic review and network meta-analysis

**DOI:** 10.1038/s41598-023-40222-5

**Published:** 2023-08-22

**Authors:** Bruna de Oliveira Ascef, Matheus Oliveira Almeida, Ana Cristina de Medeiros-Ribeiro, Danieli Castro de Oliveira Andrade, Haliton Alves de Oliveira Junior, Patrícia Coelho de Soárez

**Affiliations:** 1 Departamento de Medicina Preventiva, Faculdade de Medicina - FMUSP, Universidade de Sao Paulo, Av. Dr. Arnaldo, 455 – 2º andar – sala 2214, São Paulo, SP 01246-903 Brazil; 2 Health Technology Assessment Unit, Hospital Alemão Oswaldo Cruz, São Paulo, Brazil; 3 Disciplina de Reumatologia do Hospital das Clínicas da Faculdade de Medicina da Universidade de Sao Paulo, São Paulo, SP Brazil; 4 Departamento de Medicina Preventiva, Faculdade de Medicina - FMUSP, Universidade de Sao Paulo, São Paulo, SP Brazil

**Keywords:** Rheumatology, Outcomes research

## Abstract

What is the impact of switching between biologics and biosimilars of adalimumab, etanercept, and infliximab on efficacy and safety for rheumatoid arthritis? A systematic review and network meta-analysis were performed to compare switching and non-switching groups of treatments. Pooled Risk Relative (RR) or standardised mean differences (SMD) with 95% credible intervals (95% CrIs) were obtained. Seventeen randomized trials with a switching phase involving 6,562 patients were included. Results showed that a single switch from biologics to biosimilars compared to continuing biologics had comparable effects for primary and co-primary outcomes, the American College of Rheumatology criteria with 20% response (ACR20) (7 trials, 1,926 patients, RR 0.98, 95% CrIs 0.93 to 1.03) and the Health Assessment Questionnaire—Disability Index (HAQ-DI) (5 trials, 1,609 patients, SMD − 0.07, 95% CrIs − 0.23 to 0.1), and within the equivalence margins: ACR20 [RR 0.94, 1.06] and HAQ-DI [SMD − 0.22, 0.22]. The risk of treatment-emergent adverse events, discontinuation, and positive anti-drug antibodies were comparable after switching. Safety results were imprecise, and the follow-up period might not be sufficient to evaluate long-term effects, especially malignancies. Overall, the practice of single switching between approved biologics and biosimilars of Tumour Necrosis Factor inhibitors is efficacious and safe for rheumatoid arthritis.

## Introduction

Biosimilars have emerged into the global market as a cost-saving option and have taken their place in the optimization of the current clinical management of rheumatoid arthritis, a lifelong disease that can lead to severe joint damage and disability^1–3^. Biosimilars are complex molecules intended to be highly similar in terms of quality, safety, and efficacy to an already licensed drug (referred to as a reference biologic drug)^4^. The use of tumour necrosis factor inhibitors (TNFIs) biosimilars for patients using biologic disease-modifying antirheumatic drugs has been the standard of care for rheumatoid arthritis in areas of the world where biosimilars are available^1,3,5^. In situations where a patient will have their treatment exchanged or replaced by another treatment, the decision-making on switching should be based on the best level of evidence available^1^.

The key question about biosimilars is whether switching from the reference biologic to a biosimilar or within multiple switching scenarios would affect the response to the treatment^1,6–9^. In clinical practice, concerns arose with switching which possibly could lead to increased immune reactions, loss of efficacy, and/or more risk of safety issues^10^. There is no scientific rationale to support or refute these concerns^1^. Multiple switching studies have been reported with the conclusion that switching between reference biologics and biosimilars is reasonable^11^ and some authors have argued that there is already available a large body of clinical studies on switching that would be enough to convince the medical community about the switching of reference biologics and biosimilars^10–12^. Previous systematic reviews have attempted to gather this evidence, but most of the results are either descriptive or qualitative^11–17^, and the unique pooled estimates available included a restricted number of trials and participants^18^.

The typical switching study involves a three-arm trial in which patients in the reference biologic group are re-randomized either to continue in the biologic group or to switch to the biosimilars and in parallel, patients initially allocated to the biosimilar group continue to receive a biosimilar throughout the study period^11,19,20^. A standard meta-analysis restricted to head-to-head comparisons would be not suitable for comparisons of multi-arms trials. In this case, network meta-analysis (NMA), which is a generalization of pair-wise meta-analysis, can be used to synthesize a greater share of the available evidence and provide clinically relevant estimates to better support decision-making^21,22^. Therefore, we aimed to assess the impact of switching on the treatment of rheumatoid arthritis through a network of evidence of head-head comparisons of switching and non-switching arms of TNFi biologics and biosimilars.

## Methods

### Protocol and registration

Previously, we registered this systematic review (PROSPERO: CRD42019137155) and published a single protocol^20^ for two objectives: 1) efficacy and safety (objective 1) and switching (objective 2). Results regarding objective 1 have been published elsewhere^23^. Here, we present the study for objective 2. All changes in the protocol were explicitly mentioned as ad-hoc modifications (see Supplementary Methods file 1—Table S1). We reported the preferred reporting items for systematic reviews incorporating network meta-analysis (PRISMA-NMA) (Supplementary Methods file 2—Table S2)^24^.

### Eligibility criteria and selection process

Eligible participants were patients with rheumatoid arthritis. Interventions of interest were biosimilars of adalimumab, etanercept, and infliximab. Comparators of interest were the reference biologic drugs (i.e., adalimumab, etanercept, and infliximab originals). We included randomized controlled trials (RCT) or quasi-RCT that had at least a period (re-randomized or the open-label extension) assessing the impact of switching between treatments. Two investigators independently assessed titles, abstracts, and full-length articles against the eligibility criteria. The full description of eligibility criteria and selection process are given in Supplementary Methods file 3 and Table S3.

### Evidence sources and search strategy

The detailed evidence sources and search strategy are provided in Supplementary Methods file 4. We systematically searched in MEDLINE via PubMed, EMBASE, Cochrane Central Register of Controlled Trials, and Latin American and Caribbean Health Science from database inception to April 2021. We also searched for unpublished/ongoing trials in four trial registry databases and performed citation searches.

### Data extraction

The data collection process and the list of variables extracted are shown in Supplementary Methods file 5. Briefly, two reviewers independently extracted all pertinent quantitative data per study arm. If available, we used data referred to as the Per-Protocol (PP) population as it is the most conservative approach to assess equivalence between treatments^25^.

### Outcomes measures

We previously prespecified the outcomes of interest elsewhere^20^ In summary, we assessed the impact of switching on efficacy (encompassing clinician and patient-reported outcomes), safety, and immunogenicity. For this study, the time point of interest for all outcomes is at 6 months after the first switch (i.e., 6 months after re-randomization or 6 months after the first switch on the open-label extension phase). If efficacy outcome data were reported at different time points, we used the time point closest to 6 months after the first switch.

### Primary and co-primary outcomes

The prespecified primary efficacy endpoint was the treatment success at 6 months after the first switch, according to the American College of Rheumatology 20% response criteria (ACR20). We prespecified as a co-primary outcome the Health Assessment Questionnaire—Disability Index (HAQ-DI) 6 months after the first switch (see Supplementary Methods file 6 for details).

### Secondary outcomes: efficacy

As prespecified, we assessed seven secondary outcomes that capture disease activity, one measures functional capacity/quality of life, and one assesses structural damage (Supplementary Methods file 7).

### Secondary outcomes: safety and immunogenicity

The prespecified safety and immunogenicity outcomes included: the proportion of patients with treatment-emergent adverse events (TEAEs), serious TEAEs, special adverse events, mortality, overall discontinuation rates, positive anti-drug antibodies (ADAs) formation, and positive neutralizing antibodies (Nabs) (further details see Supplementary Methods file 8).

### Assessment of risk of bias

We used the recommendations of Moots et al.^26^ and the Food Drug Administration guidance^19^ to assess the risk of bias across six specifics domains of switching trials: (1) Randomized and blinded design with appropriate control arms; (2) At least 1-way switch from originator to a biosimilar; (3) The assessment of immunogenicity; (4) The washout period between treatment; (5) Enough power to assess efficacy and safety (equivalence phase), and (6) Enough follow-up periods. A detailed description is given in Supplementary Methods File 9 and Table S4.

### Geometry of network

We identified four general types of switching and non-switching groups in the included trials as follows:

- *Ref → Ref* patients taking reference biologics drugs continued the treatment (non-switching group).
- *Bios → Bios* patients taking biosimilars continued the treatment (non-switching group).
- *Ref → Bios* patients taking reference biologics drugs switched to biosimilars (switching group).
- *Bios → Ref* patients taking biosimilars switched to reference biologics drugs (switching group).

Our NMA model provides six or three possible pairwise comparisons estimating the comparative efficacy, safety, and immunogenicity within and between switching and non-switching groups. We treated the multi-arm studies as multiple independent two-arm studies in NMA. We assessed the transitivity (similarity) assumption by comparing the main methodological and clinical characteristics across all eligible trials by arm level. A network plot linking the switching and non-switching arms of biosimilars, and reference biologics drugs was constructed to indicate the level of pair-wise comparison.

## Data synthesis

### Pairwise and network meta-analysis

We conducted a pairwise and NMA with multi-arm trials within a Bayesian random-effects framework and a frequentist fixed-effects approach which both accounted for the correlation between the treatment difference effect for each group of multi-arm trials^27^. We selected the Bayesian random effect model for NMA as the primary analysis because it is usually the most conservative option and Bayesian estimates can be interpreted in terms of probabilities and give a framework that supports decision-making^21^.

The approaches to approximate means and standard deviations from the reported statistics are shown in Supplementary Methods file 10. Binary outcomes were summarized using the Risk Relative (RR) as a metric, whereas continuous outcomes were summarized as standardised mean difference (SMD).

A Bayesian random-effects NMA based on the Markov chain Monte Carlo (MCMC) simulation from the posterior distribution was applied to estimate the relative effects. For binary outcomes, we used binomial likelihood and modeled the log relative risk directly. For continuous outcomes, we used the normal likelihood and the identity link. Given the head-to-head comparisons (e.g., two active interventions), we assumed non-informative but biologically plausible priors for treatment effects. Details of model fitting, model diagnostics, and estimation methods are presented in Supplementary Methods file 11.

We presented summary treatment effect estimates and between-trial variance derived from the median and corresponding 95% credible intervals (CrIs) from the 2.5th and 97.5th percentile of the posterior distribution.

Bayesian pairwise meta-analysis (direct evidence) was performed to assess the consistency of NMA. As treatments included in each network of trials have inherent variability, we assumed common τ^2^ across comparisons in both NMA and inconsistency models. We contrasted the posterior summaries and deviance information criteria (DIC) of the inconsistency and consistency models^28^.

As a sensitivity analysis, we performed a consistency model of NMA within a frequentist approach and using fixed-effects models. For continuous outcomes, we used the inverse-variance model. For safety or immunogenicity outcomes, we used the Mantel–Haenszel method. The results were summarized with 95% Confidence Intervals (CI).

We performed subgroup analyses by type of reference molecule (infliximab, etanercept, and adalimumab) for all efficacy outcomes.

We performed a visual inspection of comparison-adjusted funnel plots for each outcome (≥ 10 studies) to investigate the association between trial size (precision) and treatment effects. Comparison-adjusted funnel plots were considered symmetrical when about the zero line there were no small-sample effects present.

We used Stata 16.0. and WinBUGS software (version 1.4.3, MRC Biostatistics Unit, Cambridge, UK). Network plots were generated using Stata 16.0.

### Margins of equivalence

We estimated the posterior probabilities of equivalence for the primary (ACR20) and co-primary (HAQ-DI) outcomes using prespecified margins of equivalence computed from large placebo-controlled trials and endorsed by experienced rheumatologists^20^. The observation that 95% CrIs fall entirely to two sides of the margins of equivalence defines equivalence^20^. For the ACR20 outcome, we assumed an equivalence margin on the relative risk of [0.94, 1.06]. For the HAQ-DI outcome, the equivalence margin on SMD units was [-0.22,0.22]. Thus, the probability of equivalence was defined as the proportion of Markov Chain Monte Carlo simulations in which the random-effects summary estimate was contained within the equivalence margins.

### Assessment of overall certainty of evidence

The Grading of Recommendations Assessment, Development and Evaluation (GRADE) 4-step approach was used for rating the quality of effect estimates from the NMA of each outcome (Supplementary Methods file 12)^29,30^.

## Results

### Study selection and characteristics

The evidence flow diagram (Fig. S1) and description of the study selection process are provided in Supplementary Results file 1. In total, 17 randomized controlled trials^31–70^ assessed equivalence with at least one switching phase. Among these, three trials had a subsequent-second switching phase (Table S5).

**Figure 1:**
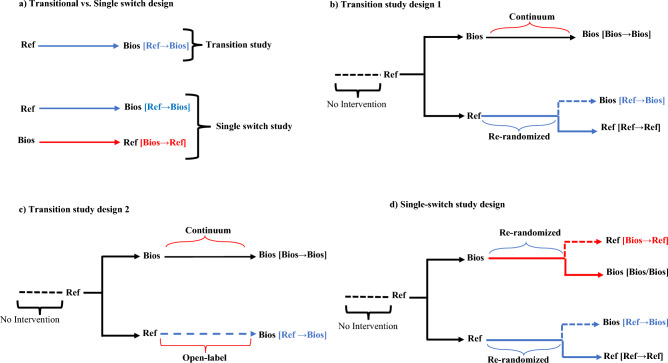
Main switching study designs of included studies. Figure 1a shows a transitional study design in which there is a single switch from a reference biologic to a biosimilar, but not the contrary. While single switch design there is a single switch from a reference biologic to a biosimilar and from a biosimilar to a reference biologic. Figure 1b shows Transition study design 1 (two non-switching groups as a control): Trials in which there is a single switch from one treatment (biologic drug) to another (biosimilar drug), but not the contrary. Firstly, patients were randomly allocated to either a biosimilar or a biologic drug (first period). Then, in the second period, the trial became a three-arm trial in which patients in the biologic drug group were re-randomized either to continue in the biologic group or to switch to the biosimilar drug treatment. Patients initially allocated to the biosimilar group continued to receive a biosimilar throughout the study period. Figure 1c shows Transition study design 2 (randomized trials with an open-label extension; single non-switching group as a control): Trials in which there is a single switch from a biologic drug to a biosimilar drug, but not the contrary. Firstly, patients were randomly allocated to either a biosimilar or a biologic drug (first period). Then, in the open-label extended phase (second period), all patients (intervention and control groups) received a biosimilar drug. Figure 1d shows Single-switch study design 1: Trial in which there is a single switch from each treatment to the other. Firstly, patients were randomly allocated to either a biosimilar or a biologic drug (first period). Then, in the second period, treatments were randomly switched in both directions. The groups within blue square brackets are switching groups Ref → Bios. The groups within red square brackets are switching groups Bios → Ref. The groups within black square brackets are non-switching groups Ref → Ref or Bios → Bios. Bios: biosimilar; Ref: reference biologic. Ref → Ref: patients taking reference biologics continued the treatment (non-switching group); Bios → Bios: patients taking biosimilars continued the treatment (non-switching group); Ref → Bios: patients taking reference biologics drugs switched to biosimilars (switching group); Bios → Ref: patients taking biosimilars switched to reference biologics drugs (switching group).

Table 1 summarizes the design of the first switching phase and the main baseline characteristics of patients. The median follow-up of the first switching was 28 weeks (interquartile [IQR]: 24–38). All trials had a transitional study design in which there is a single switch from a reference biologic to a biosimilar, but not the contrary, except in two trials^31–37,60^. In these last, there is a single switch from each treatment to the other (Fig. 1a). Six transitional trials^40,42,44,45,50,68,70^ had three arms in which patients in the reference biologic group were re-randomized either to continue in the reference biologic group or to switch to the biosimilar treatment and patients initially allocated to the biosimilar group continued to receive a biosimilar (Fig. 1b)Nine transitional trials^39,47,53,55,57,59,62,68,69^ were designed with two arms and in an open-label extended phase in which all patients received the biosimilar drug (Fig. 1c). In only one trial^35–37^, treatments were randomly switched in both directions (four-arms trial) (Fig. 1d). There were seven trials assessing biosimilars of adalimumab (3,698 patients), five for etanercept biosimilars (1,316 patients), and five for infliximab biosimilars (1,549 patients).

**Table 1:** First switching study design and arms comparisons, and the main baseline characteristics of patients per arm.

Authors	Study design (follow-up, weeks)	Arms (No of participants)	Baseline (n)	Age, mean, years	Female, n (%)	BMI, mean (kg/m2)	RA duration, mean (years)	RF positive, n (%)	CRP, mean (mg/dL)	MTX dose, mean (mg/week)	Previous MTX, n (%)	Previous bDMARDS, n (%)	Previous Corticosteroids, n (%)
Alten ^31–37^	Single switch 1(30)	FKB327/FKB327 (216)	216	52.7	162 (75)	–	–	–	–	16.2	–	34 (15.7)	127 (58.8)
		FKB327/ADA (108)	108	52.1	85 (79)	–	–	–	–	15.5	–	21 (19.4)	70 (64.8)
		ADA/ADA (213)	213	54.0	171 (80)	–	–	–	–	15.7	–	39 (18.3)	137 (64.3)
		ADA/FKB327 (108)	108	52.3	83 (77)	–	–	–	–	16.2	–	24 (20.4)	69 (63.9)
Cohen ^38,39^	Transitional design 2 (72)	ABP-501/ABP-501 (230)	230	54.7	188 (82)	–	9.1	–	–	–	–	60 (26.1)	–
		ADA/ABP-501 (237)	237	56.1	191 (81)	–	9.5	–	–	–	–	69 (29.1)	–
Fleischmann ^40–42^	Transitional design 1 (26)	PF-06410293/PF-06410293 (283)	283	51.3	229 (81)	27.5	6.9	–	–	15.2	–	8 (2.8)	155 (54.8)
		ADA/ADA (135)	135	53.6	108 (80)	28.4	7.1	–	–	15.7	–	4 (3.0)	77 (57)
		ADA/PF-06410293 (134)	134	53.4	95 (71)	27.5	6.6	–	–	14.7	–	1 (0.7)	80 (59.7)
Cohen ^43,44^	Transitional design 1 (34)	BI-695501/BI-695501 (298)	–	–	–	–	–	–	–	–	–	–	–
		ADA/ADA (148)	–	–	–	–	–	–	–	–	–	–	–
		ADA/BI-695501 (147)	–	–	–	–	–	–	–	–	–	–	–
Weinblatt ^45,46^	Transitional design 1 (28)	SB5/SB5 (254)	271	49.8	200 (74)	26.2	5.4	203 (75)	0.6	15.1	–	–	–
		ADA/ADA (129)	129	52.8	103 (80)	26.9	5.6	94 (73)	0.5	15.2	–	–	–
		ADA/SB5 (125)	125	51.7	105 (84)	27.2	5.3	80 (64)	0.6	15.4	–	–	–
Wiland ^47,48^	Transitional design 2 (24)	GP2017/GP2017 (159)	159	52.9	135 (85)	28.5	8.0	126 (79)	–	17.2	–	–	–
		ADA/GP2017 (166)	166	53.5	132 (80)	28.0	7.1	130 (78)	–	17.6	–	–	–
Kay ^49,50^	Transitional design 1 (28)	CT-P17/CT-P17 (303)	303	53.0	232 (77)	–	6.7	–	–	–	–	–	–
		ADA/ADA (153)	153	53.0	122 (80)	–	6.6	–	–	–	–	–	–
		ADA/CT-P17 (152)	152	53.0	123 (81)	–	6.4	–	–	–	–	–	–
Emery ^51–53^	Transitional design 2 (52)	SB4/SB4 (126)	126	49.9	107 (85)	26.7	5.7	99 (79)	0.6	16.9	–	–	–
		ETN/SB4 (119)	119	52.1	107 (85)	26.1	5.8	89 (75)	0.4	16.5	–	–	–
Odell ^54,55^	Transitional design 2 (24)	CHS-0214/ CHS-0214 (284)	–	–	–	–	–	–	–	–	–	–	–
		ETN/ CHS-0214 (280)	–	–	–	–	–	–	–	–	–	–	–
Matsuno ^56,57^	Transitional design 2 (48)	LBEC0101/LBEC0101 (70)	69	52.6	53 (77)	–	8.1	48 (70)	0.2	12.6		11 (15.9)	57 (82.6)
		ETN/LBEC0101 (78)	78	54.5	69 (88)	–	7.9	52 (67)	0.3	12.6		8 (10.3)	70 (89.7)
Matucci-Cerinic ^58,59^	Transitional design 2 (24)	GP2015/GP2015 (175)	175	55.1	149 (85)	–	8.7	130 (74)	1.2	16.0	53 (30.3)	–	–
		ETN/GP2015 (166)	166	52.2	131 (79)	–	8.1	118 (71_	1.1	17.0	46 (27.7)	–	–
Yamanaka ^60^	Single switch 2 (28)	YLB113/ETN (10)	–	–	–	–	–	–	–	–	–	–	–
		ETN/YLB113 (8)	–	–	–	–	–	–	–	–	–	–	–
Yoo ^61–63^	Transitional design 2 (48)	CT-P13/CT-P13 (158)	158	50.0	125 (79)	26.8	–	–	0.4	–	–	–	–
		IFX/CT-P13 (144)	144	49.0	122 (85)	25.6	–	–	0.4	–	–	–	–
Kay ^64,65^	Transitional design 2 (38)	BOW015/BOW015 (104)	–	–	–	–	–	–	–	–	–	–	–
		IFX/BOW015 (53)	–	–	–	–	–	–	–	–	–	–	–
Choe ^66–68^	Transitional design 1 (24)	SB2/SB2 (201)	201	51.8	158 (79)	26.6	6.3	140 (70)	0.8	14.7	–	–	–
		IFX/IFX (101)	101	51.5	79 (78)	26.8	6.7	66 (65)	0.8	15.2	–	–	–
		IFX/SB2 (94)	94	53.0	77 (82)	26.3	6.3	67 (71)	0.7	14.3	–	–	–
Matsuno ^69^	Transitional design 2 (24)	NI071/NI071 (108)	–	–	–	–	–	–	–	–	–	–	–
		IFX/NI071 (102)	–	–	–	–	–	–	–	–	–	–	–
Genovese ^70^	Transitional design 1 (28)	ABP710/ABP710 (244)	–	–	–	–	–	–	–	–	–	–	–
		IFX/IFX (121)	–	–	–	–	–	–	–	–	–	–	–
		IFX/ABP710 (119)	–	–	–	–	–	–	–	–	–	–	–

RA: rheumatoid arthritis; ADA: adalimumab; ETN: etanercept; IFX: Infliximab; BMI: body mass index; RF: Rheumatoid factor; CRP: C reactive protein; Single-switch design: Trials in which there is a single switch from each treatment to the other. Firstly, patients were randomly allocated to either a biosimilar or a biologic drug (first period). Then, in the second period, treatments were randomly switched in both directions. Single switch 2: in the study of Yamanaka et al. ^60^, patients were randomly allocated to either a biosimilar or a biologic drug (stage A). Then, in parallel, a group of patients continued the treatments to evaluate long-term safety and immunogenicity (Stage B) and selected patients were crossed over in both directions (switch group/Stage C). However, it was not clear if stage C was generated randomly. Transition design 1: Trials in which there is a single switch from one treatment (biologic drug) to another (biosimilar drug), but not the contrary. Firstly, patients were randomly allocated to either a biosimilar or a biologic drug (first period). Then, in the second period, the trial become a three-arm trial in which patients in the biologic drug group were re-randomized either to continue in the biologic group or to switch to the biosimilar drug treatment. Patients initially allocated to the biosimilar group continue to receive a biosimilar throughout the study period. Transition design 2: Trials in which there is a single switch from a biologic drug to a biosimilar drug, but not the contrary. Firstly, patients were randomly allocated to either a biosimilar or a biologic drug (first period). Then, in the open-label extended phase (second period), all patients (intervention and control groups) received the biosimilar drug.

In total, 17 trials provided data on 6,562 patients with moderate to severe rheumatoid arthritis with previous use of methotrexate. The median of participants per arm was 120 (IQR: 123–204) (Table 1). Among 12 trials with available baseline data, patients’ characteristics were well balanced across switching and non-switching arms. The median (IQR) age of the patients was 53 years old (52–53) and a median of 80% female (IQR: 78–82), and patients had rheumatoid arthritis with a median of 7 years (IQR: 6–8) and with an average of 15.5 mg methotrexate (IQR:15.1–18.1) used per week before study enrolment. The therapy schemes were kept the same from the efficacy and safety phases.

### Risk of bias in switching trials

**Figure 2:**
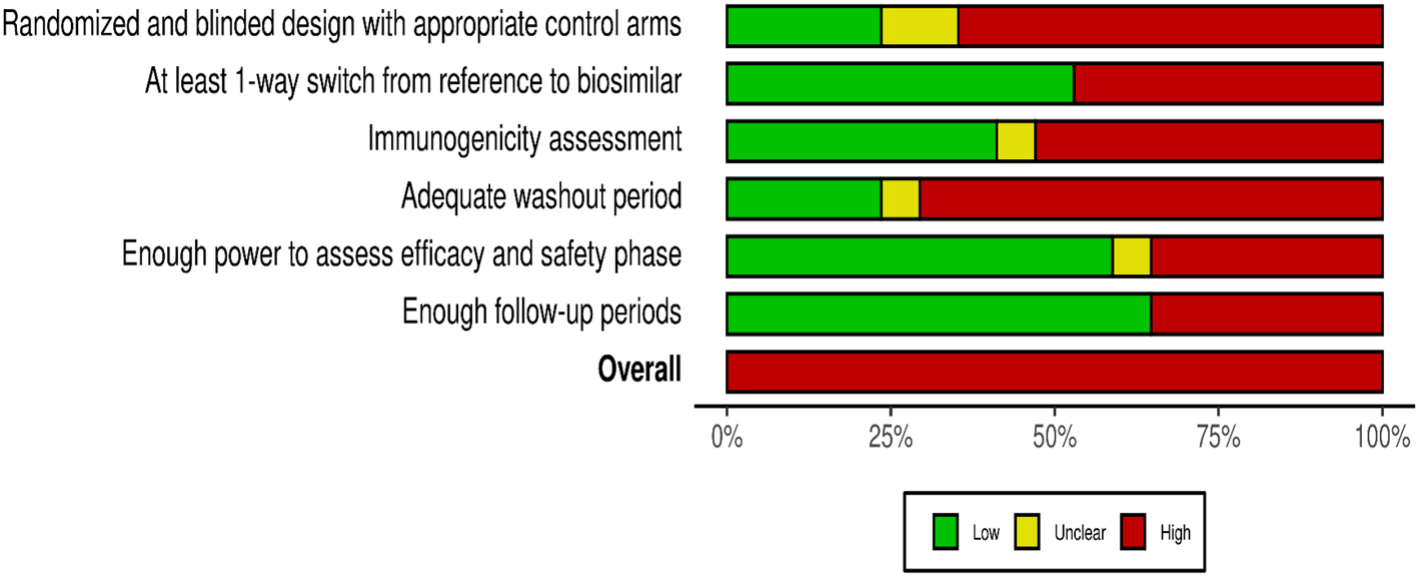
Summary plot of the risk of bias of specific domains of switching studies. Based on the judgment of risk of bias in 17 switching trials of biosimilars and reference biologic drugs. Each domain was judged by two independent reviewers as low, unclear, or high risk of bias.

Figure 2 presents the summary plot of the risk of bias. All included trials had at least one domain judged as at high risk of bias. Overall, four of 17 trials had randomization before the switch and kept period blinding with appropriate arms, nine trials conducted at least a 1-way switch from reference to a biosimilar, seven trials performed the immunogenicity assessment properly, and four had adequate washout period before the switch, 10 trials had enough power to assess the equivalence in the previous efficacy and safety phase, and 11 had enough follow-up period. The full assessment across the six specific domains of switching studies is given in Tables S6 and S7.

### Evidence synthesis

We provide the evidence synthesis divided into (1) Efficacy; (2) Safety, and (3) Immunogenicity outcomes. For each category of the outcome, the results are presented as follows: (a) The network evidence plot of eligible trials for all outcomes (Figs. S2–S6); (b) League tables summarizing Bayesian random effects NMA estimates (Tables 2, 3, 4); (c) The summary of Bayesian NMA estimates for each outcome pooled with all TNFIs together and according to the type of molecule (only for efficacy outcomes) (Table S8); (d) The summary of Bayesian NMA estimates for each outcome pooled with all TNFIs together (only for safety and immunogenicity outcomes) (Tables S9 and S10); (e) Consistency and inconsistency models, and GRADE quality ratings for each comparison of direct evidence and NMA evidence (Tables S11– S13); and (e) Assessments of small-study effects (Figs. S7–S11).

**Table 2:** Efficacy outcomes: the summary estimates from the Bayesian random-effects network meta-analysis of switching and non-switching arms of biosimilars and reference biologics drugs for arthritis rheumatoid.

Primary outcomes				
HAQ− DI				
ACR20	*Bios → Bios*	− 0.011 (− 0.350, 0.304)	− 0.065 (− 0.231, 0.098) **[96.4%]**	N/A
	0.984 (0.938, 1.025) **[97.1%]**	*Ref → Ref*	− 0.076 (− 0.422, 0.243)	N/A
	0.997 (0.963, 1.032) [99.8%]	0.982 (0.930, 1.026) [94.8%]	*Ref→Bios*	N/A
	0.989 (0.839, 1.113) [61.8%]	0.973 (0.821, 1.092) [60.1%]	0.992 (0.841, 1.117) [61.4%]	*Bios→Ref*
Secondary outcomes				
ACR70				
ACR50	*Bios → Bios*	0.973 (0.853, 1.105)	1.007 (0.908, 1.110)	0.892 (0.597, 1.255)
	0.982 (0.903, 1.061)	*Ref → Ref*	0.980 (0.848, 1.120)	0.868 (0.576, 1.224)
	1.009 (0.946, 1.076)	0.992 (0.906, 1.076)	*Ref→Bios*	0.885 (0.591, 1.255)
	0.956 (0.739, 1.175)	0.939 (0.721, 1.151)	0.948 (0.730, 1.170)	*Bios→Ref*
CDAI				
SDAI	*Bios → Bios*	− 0.003 (− 0.167, 0.162)	0.016 (− 0.140, 0.169)	N/A
	− 0.002 (− 0.165, 0.162)	*Ref → Ref*	0.012 (− 0.169, 0.193)	N/A
	− 0.002 (− 0.155, 0.150)	− 0.004 (− 0.185, 0.175)	*Ref→Bios*	N/A
	N/A	N/A	N/A	*Bios→Ref*
DAS28− CRP				
DAS28− ESR	*Bios → Bios*	0.048 (− 0.124, 0.209)	0.007 (− 0.106, 0.128)	− 0.007 (− 0.340, 0.318)
	− 0.075 (− 0.214, 0.069)	*Ref → Ref*	0.055 (− 0.118, 0.225)	0.040 (− 0.303, 0.368)
	0.019 (− 0.081, 0.119)	− 0.056 (− 0.204, 0.099)	*Ref→Bios*	− 0.015 (− 0.354, 0.311)
	N/A	N/A	N/A	*Bios→Ref*
mTRSS	*Bios → Bios*	N/A	N/A	N/A
	− 0.096 (− 0.431, 0.225)	*Ref → Ref*	N/A	N/A
	− 0.017 (− 0.268, 0.286)	− 0.111 (− 0.425, 0.236)	*Ref→Bios*	N/A
	N/A	N/A	N/A	*Bios→Ref*

Abbreviations in italics represent non-switching arms and switching arms. The values given are RR [95% CrIs]) for binary outcomes, in which RR greater than 1 favors the arm in the column; and SMD (95% CrIs) for continuous outcomes, in which SMD less than 0 favors the arm in the row. For primary outcomes, ACR20 and HAQ-DI, the posterior probability of equivalence (%) of each pairwise comparison were given in bold, if available. Pr (E): the posterior probability of equivalence was based on the prespecified margins of equivalence for ACR20 response [0.94, 1.06]; and for HAQ-DI on a standardised mean difference scale [−  0.22, 0.22].

Ref-Ref: patients taking reference biologics drugs continued the treatment (non-switching group); Bios-Bios: patients taking biosimilars continued the treatment (non-switching group); Ref-Bios: patients taking reference biologics drugs switched to biosimilars (switching group); Bios-Ref: patients taking biosimilars switched to reference biologics drugs (switching group).

ACR20: the American College of Rheumatology 20% response criteria; HAQ-DI: the Health Assessment Questionnaire—Disability Index; ACR50: the ACR 50% response criteria; ACR70: the ACR 70% response criteria; CDAI: clinical disease activity score; SDAI: simplified disease activity score; DAS28-ESR: disease activity score in 28 joints based on the erythrocyte sedimentation rate; DAS28-CRP: DAS28 based on C-reactive protein; mTRSS: Sharp-Van Der Heidje Modified Score Methods; RR: relative risk; SMD: standardized mean differences; CrIs: credible intervals; N/A: not available.

**Table 3:** Safety outcomes: Bayesian random-effects network meta-analysis of switching and non-switching arms of biosimilars and reference biologics drugs for arthritis rheumatoid.

Safety outcomes				
Serious TEAE				
TEAE	*Bios → Bios*	0.882 (0.461, 1.542)	1.171 (0.739, 1.901)	2.020 (0.627, 5.515)
	0.921 (0.772, 1.104)	*Ref → Ref*	1.032 (0.537, 1.852)	1.796 (0.524, 4.495)
	1.082 (0.949, 1.233)	0.996 (0.826, 1.203)	*Ref→Bios*	1.729 (0.526, 4.707)
	1.126 (0.734, 1.661)	1.035 (0.678, 1.547)	1.039 (0.675, 1.552)	*Bios→Ref*
IRRs				
Hypersensitivity	*Bios → Bios*	1.032 (0.334, 3.216)	0.822 (0.336, 2.038)	N/A
	0.914 (0.183, 4.177	*Ref → Ref*	0.847 (0.262, 2.848)	N/A
	0.727 (0.087, 3.442)	0.657 (0.079, 3.089)	*Ref→Bios*	N/A
	N/A	N/A	N/A	*Bios→Ref*
Serious Infections				
Malignancies	*Bios → Bios*	1.809 (0.442, 6.200)	1.286 (0.680, 2.744)	N/A
	0.783 (0.161, 3.136)	*Ref → Ref*	1.809 (0.442, 6.200)	N/A
	1.078 (0.337, 3.361)	0.847 (0.162, 3.447	*Ref→Bios*	N/A
	N/A	N/A	N/A	*Bios→Ref*
Discontinuation rates	*Bios → Bios*	N/A	N/A	N/A
	1.176 (0.869, 1.574)	*Ref → Ref*	N/A	N/A
	0.931 (0.748, 1.152)	1.094 (0.787, 1.500)	*Ref→Bios*	N/A
	0.603 (0.264, 1.234)	0.707 (0.307, 1.463)	0.648 (0.284, 1.338)	*Bios→Ref*

Abbreviations in italics represent non-switching arms and switching arms. The values given are RR [95% CrIs]) for binary outcomes, in which RR greater than 1 favors the arm in the column.

Ref-Ref: patients taking reference biologics drugs continued the treatment (non-switching group); Bios-Bios: patients taking biosimilars continued the treatment (non-switching group); Ref-Bios: patients taking reference biologics drugs switched to biosimilars (switching group); Bios-Ref: patients taking biosimilars switched to reference biologics drugs (switching group). TEAEs: treatment-emergent adverse events; IRRs: infusion-related reactions; RR: relative risk; CrI: credible intervals; N/A: not available.

**Table 4:** Immunogenicity outcomes: Bayesian random-effects network meta-analysis of switching and non-switching arms of biosimilars and reference biologics drugs for arthritis rheumatoid.

Immunogenicity outcomes				
Nabs				
ADAs	*Bios → Bios*	0.835 (0.559, 1.263)	0.882 (0.605, 1.285)	0.927 (0.420, 1.986)
	0.893 (0.744, 1.065)	*Ref → Ref*	0.738 (0.475, 1.153)	0.774 (0.351, 1.687)
	0.993 (0.855, 1.158)	0.888 (0.729, 1.078)	*Ref→Bios*	1.050 (0.468, 2.301)
	1.355 (0.878, 2.045)	1.211 (0.782, 1.835)	1.362 (0.881, 2.071)	*Bios→Ref*

Abbreviations in italics represent non-switching arms and switching arms. The values given are RR [95% CrIs]) for binary outcomes, in which RR greater than 1 favors the arm in the column.

Ref-Ref: patients taking reference biologics drugs continued the treatment (non-switching group); Bios-Bios: patients taking biosimilars continued the treatment (non-switching group); Ref-Bios: patients taking reference biologics drugs switched to biosimilars (switching group); Bios-Ref: patients taking biosimilars switched to reference biologics drugs (switching group)*;* RR: relative risk; CrIs: credible interval; N/A: not available; ADAs: Positive anti-drug antibodies; Nabs: Positive neutralizing antibodies.

### Network structures

**Figure 3:**
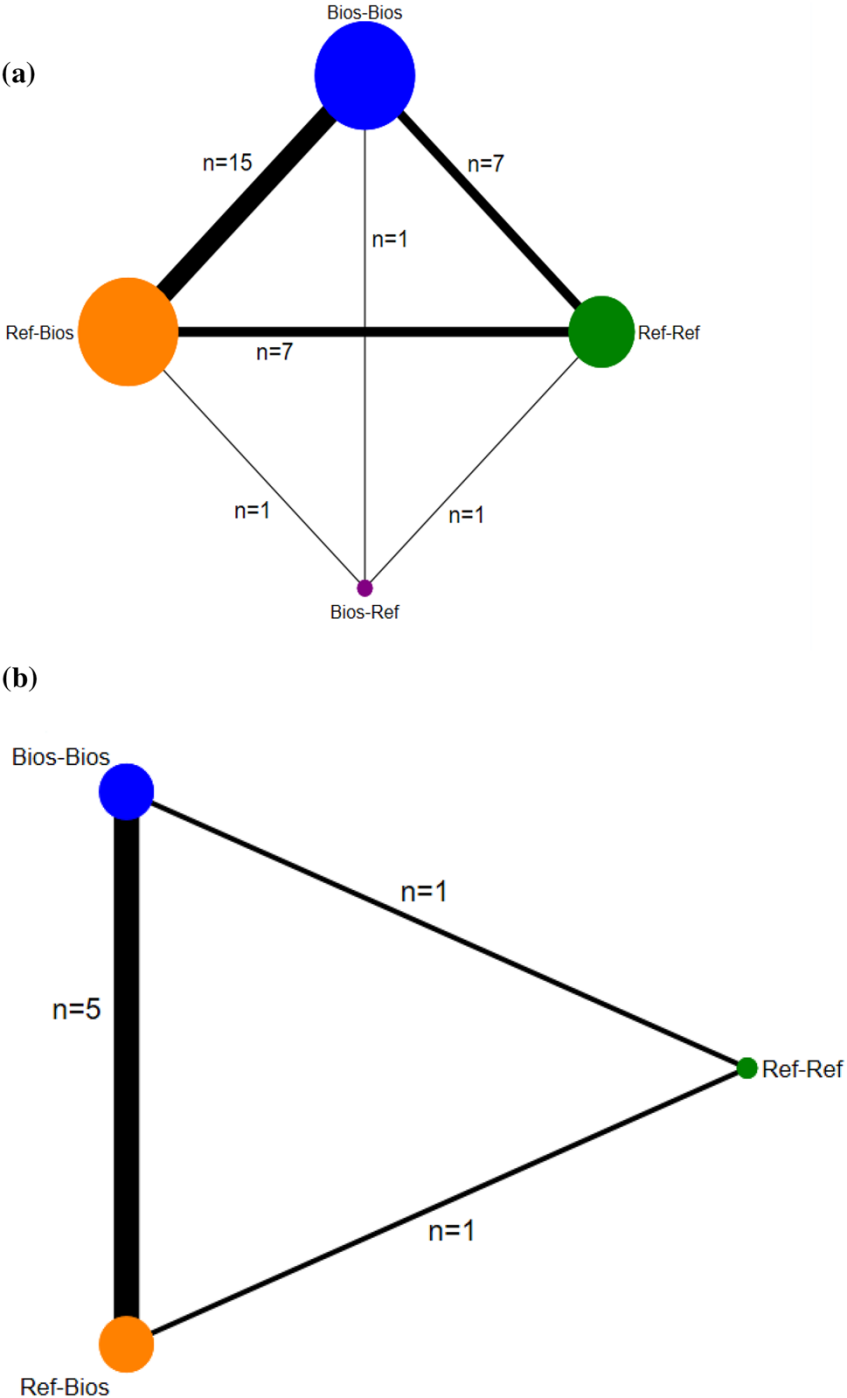
Possible treatment networks for a) ACR20 and b) HAQ-DI.The graph in Fig. 3** (panel A)** comprises four nodes representing two non-switching arms (Ref-Ref, Bios-Bios) and two switching arms (Ref-Bios and Bios-Ref), and six edges (arrows). Figure 3 (panel B) comprises three nodes representing two non-switching arms (Ref-Ref, Bios-Bios) and one switching (Ref-Bios), and three edges (arrows). The size of nodes is proportional to the number of patients analyzed for interventions. The thickness of lines is proportional to the number of studies contributing to the direct comparison. ACR20: the American College of Rheumatology 20% response criteria; HAQ-DI: the Health Assessment Questionnaire—Disability Index (HAQ-DI). Ref -Ref: patients taking reference biologics drugs continued the treatment (non-switching group); Bios-Bios: patients taking biosimilars continued the treatment (non-switching group); Ref-Bios: patients taking reference biologics drugs switched to biosimilars (switching group); Bios-Ref: patients taking biosimilars switched to reference biologics drugs (switching group).

Figure 3 presents the network evidence plots for ACR20 and HAQ-DI and Figs. S2–S6 for all other outcomes. The nodes show the switching and non-switching arms compared, and the edges show the available direct comparisons among the switching and non-switching arms. Two possible network structures consist of closed loops formed by multi-arm trials of four nodes as represented in Fig. 3 (panel a) for ACR20, and three nodes as in Fig. 3 (panel b) for HAQ-DI. The pairwise comparison contributing most to the network was Bios-Bios (non-switching arm) versus Ref-Bios (switching arm) and least, all comparisons that had the Bios-Ref as the switching arm.

### Impact of switching on the efficacy

Table 2 presents the results of random-effects summary estimates of all efficacy outcomes 6 months after the first switching. Bayesian 95% Crls were consistently compatible with the hypothesis of no difference between switching and non-switching arms, with all 95% encompassing the null effect (i.e., an RR = 1.0) with low heterogeneity (τ^2^ = 0.0) for all efficacy outcomes**.** Similar findings in the direction of the null effect are expected in future exchangeable studies (Table S8).

### Primary outcome (ACR20)

The Bayesian NMA for rates of ACR20 included 15 switching trials, 6,007 participants treated with either biosimilar or reference biologics of TNFIs, and four nodes (Fig. 3, panel a). The Bayesian random-effects summary of all pair comparisons crossed the line of null effect with no evidence of heterogeneity (τ^2^ = 0.0). The 95% Bayesian CrIs for three pair-wise comparisons were entirely contained within the [0.94 to 1.06] prespecified equivalence margins. The effect estimates for other comparisons involving the switching arm (Bios-Ref) were less imprecision because there was only one study, but still compatible with the hypothesis of equivalence. The posterior probability of equivalence varies from 61.4 to 99.8% with the lowest probability in comparisons with Bios-Ref (Table 2).

### Co-primary outcome (HAQ-DI)

The Bayesian NMA for the HAQ-DI outcome included five switching trials, 1,732 participants, and three nodes (Fig. 3, panel b). The average effects on HAQ-DI scores were equivalent in those patients switching from reference to a biosimilar (Ref-Bios) compared to those patients that continued the treatment with biosimilars (Bios-Bios) (5 trials, 1,609 patients: SMD -0.07, 95%CrIs -0.23 to 0.1, τ^2^ = 0.01). For this comparison, the posterior probability of equivalence was 96.4%. For the other comparisons, there is a lower probability of equivalence because only one trial assessed the non-switching arm Ref-Ref, and CrIs crossed the bounds of the equivalence margin (Tables 2 and S8).

### Secondary outcomes of efficacy

We found similar effects within or between switching and non-switching arms, with Bayesian 95% CrIs crossing the null effect for all secondary efficacy outcomes of disease activity (ACR50, ACR70, CDAI, SDAI, DAS28-ESR, and DAS28-CRP) and the prevention of structural damage progression (mTRSS) (Tables 2 and S8). We did not conduct NMA for SF-36 because there were not enough trials (n = 2).

### Additional subgroup analysis of efficacy

The results of subgroup analysis by type of TNFIs molecule are consistent but less precise when compared with all TNFIs pooled together and with higher heterogeneity between trials. Overall, the multi-arms trials from adalimumab contributed more to the network evidence, followed by infliximab, and least, etanercept (Table S8).

### Impact of switching on safety 

The Bayesian NMA for the overall TEAE and discontinuation rates included 15 switching trials involving more than 6400 patients and four nodes (Fig. S4). The evidence showed that the risk for both outcomes was similar within and between switching and non-switching arms. The 95% CrIs for the risk for serious TEAE, hypersensitivity, IRRs, malignancies, and serious infections crossed the null effect but were imprecise (small number of events and high heterogeneity between trials). We are unable to perform NMA for ISRs, active tuberculosis, and mortality due to rare event rates (n < 30) (Tables 3 and S9).

### Impact of switching on immunogenicity

The Bayesian NMA for the risk of ADAs included 16 switching trials, 6,006 patients, and four nodes (Fig. S5). The immunogenicity profiles of patients taking either biosimilars or reference biologics were similar within and between switching and non-switching arms (Tables 4 and S10).

### Exploration of inconsistency

By contrasting the inconsistency and consistency models, we did not find any evidence of inconsistency in summary effect estimates (Table S11). The absolute differences between values of DIC of Bayesian inconsistency and consistency models were inferior to 3 for all outcomes and were considered not important (Table S12).

### Sensitivity analyses

The results were comparable, and all conclusions are unchanged using Frequentist fixed-effect approach for NMA, which did not consider heterogeneity between the studies (Tables S11 to S13).

### Publication bias

We found suspected asymmetry in the comparison-adjusted funnel plots for ACR20, ACR50, ACR70, and serious TEAE, suggesting a higher probability of publication bias (Figs. S7, S8, and S10).

### Certainty of evidence

There was low to moderate certainty that switching between biologics and biosimilars of TNFIs results in little to no differences in effects on efficacy when compared to those continuing the original treatments. There was moderate certainty that the switching arm (Ref-Bios) had similar rates of TEAE compared to the non-switching arms. There was low to moderate certainty that rates of discontinuation and formation of ADAs and Nabs were similar within or between switching and non-switching arms. There was very low certainty about the impact of switching on the risk of serious TEAE, and special adverse of interest due to a low number of events and high heterogeneity between trials (Table S12).

## Discussion

To the best of our knowledge, this is the most comprehensive systematic review and NMA within a Bayesian framework to evaluate the impact of switching between biosimilars of adalimumab, etanercept, and infliximab and its reference biologics drugs on rheumatoid arthritis treatment. Data were obtained from 17 switching trials including 6,562 patients with moderate to severe rheumatoid arthritis. Overall, the evidence from the NMA showed that neither efficacy, safety, nor immunogenicity was affected by switching. Although the precision of safety and immunogenicity findings was limited, the duration of follow-up may be inadequate for a comprehensive assessment of long-term effects, particularly concerning malignancy risks.

We would like to highlight the main findings from our NMA. First, the clinical response or functional capacity of patients after switching was equivalent within and between switching and non-switching groups, and all comparisons met prespecified margins of equivalence. Second, there is evidence of a similar risk of experiencing TEAE or discontinuing study rates i.e., comparable safety and tolerability were observed after switching. Given the rare rates of some adverse events, however, we are uncertain about the effects of switching in these cases. Third, the immune response was comparable for patients in both switching and non-switching treatment arms.

The robust findings from our NMA add to the other systematic reviews^11–14,16–18^ providing together enough evidence to support switching between reference biologics and biosimilars of TNFIs in patients with rheumatoid arthritis. This conclusion is in line with publications of the main medical societies of rheumatoid arthritis supporting the single switch from a reference biologic to an approved biosimilar in clinical practice^1,3,5,7^. In the opposite line, Numan and Faccin^15^ showed that the evidence from 98 switching studies was inconclusive and inconsistent, including 10 randomized trials and 30 real-world studies involving rheumatoid arthritis populations. The authors argued that included studies had divergent rates of discontinuation and a lack of key design elements for assessing switching. In our study, there was evidence of similar discontinuation rates between patients who switched treatments and those who continued the original treatment. Although we extended the finding that these trials were at high risk of bias, new studies should attempt to incorporate essential aspects of quality and reporting when assessing the impact of switching between treatments.

Our study provides novel evidence regarding switching from a biosimilar to a reference, i.e., switching back to the original treatment as being an efficacious and safe practice. Even though, the confidence of the effects estimates for this type of switch was limited by the low number of trials available. Of note, our evidence does not address the issue of switching from a biosimilar to another biosimilar of the same reference biologic drugs or switching multiple times. These questions remained to be answered and should be the object of future research^1,8^.

Proper interpretation of the results of our NMA requires consideration of some of its features and limitations. First, several comparisons such as those involving the switching arm (Bios-Ref) included only one or few studies, limiting the confidence of estimates. Second, special attention should be given to the interpretation of some safety and immunogenicity outcomes, which indicated the null effect between switching and non-switching arms. The switching phase duration varied between trials, and for some outcomes such as serious infections, data were sparse (low rates of events and wide CrIs) with high heterogeneity between studies. Third, we included trials from three different molecules of the same drug class, so possibly we could not detect differences inherent to each molecule. Indeed, we did not observe any systematic differences in the main demographic and clinical characteristics of the populations analyzed. Also, we did not identify any systematic differences across trials since we included RCT of equivalence followed by a switching phase. We did not find any inconsistency between the consistency (NMA) and inconsistency model (direct evidence). Furthermore, our subgroup and sensitive analysis showed consistent results with the main analysis. Finally, this study assessed only the impact of the first switching after 6 months after re-randomization or after the open-label extension, and we cannot infer from our results a decision to a multiple switching context or long-term effects of switching.

## Conclusion

Our Bayesian NMA provided compelling evidence that switching treatments, either from a reference biologic to a biosimilar or from a biosimilar to a reference biologic of TNFIs did not impact significantly the clinical, safety, and immunogenicity responses when compared to non-switching treatments. Although safety results were imprecise and the follow-up period might not be sufficient to evaluate long-term effects, mainly regarding malignancies. Nonetheless, these findings support the rational practice of switching reference biologics and biosimilar drugs of adalimumab, etanercept, and infliximab for patients with rheumatoid arthritis.

### Supplementary Information

Supplementary Information.

## Data Availability

The datasets used and/or analyzed during the current study available from the corresponding author on reasonable request.
