# Supplementary material for: Impact of switching between reference biologics and biosimilars of tumour necrosis factor inhibitors for rheumatoid arthritis: a systematic review and network meta-analysis
Source: Sci Rep. 2023 Aug 22;13:13699. doi: 10.1038/s41598-023-40222-5 (PMC10444768; doi:10.1038/s41598-023-40222-5)
Supplement: Supplementary file 1 — Supplementary Information. [file 41598_2023_40222_MOESM1_ESM.docx]

1. **Impact of switching between reference biologics and biosimilars of Tumour Necrosis Factor inhibitors for rheumatoid arthritis: a systematic review and network meta-analysis**

Bruna de Oliveira Ascef, Matheus Oliveira Almeida, Ana Cristina de Medeiros Ribeiro, Danieli Castro de Oliveira Andrade, Haliton Alves de Oliveira Junior, Patrícia Coelho de Soárez .

SUPPLEMENTARY FILE

[METHODS 4](#_Toc140147373)

[Supplementary Methods file 1. Changes to the protocol with reasons. 4](#_Toc140147374)

[Supplementary Table S1. Changes to the protocol with reasons. 4](#_Toc140147375)

[Supplementary Methods file 2 – PRISMA-NMA checklist. 5](#_Toc140147376)

[Supplementary Table S2 – PRISMA-NMA checklist. 5](#_Toc140147377)

[Supplementary Methods file 3 – Description of the eligibility criteria and study selection 8](#_Toc140147378)

[Supplementary Methods file 4 – Evidence sources and search strategy 10](#_Toc140147379)

[Supplementary Table S3 – Evidence sources and search strategy 10](#_Toc140147380)

[Supplementary Methods file 5. Data collection process 13](#_Toc140147381)

[Supplementary Methods file 6. Prespecified primary and co-primary outcomes of efficacy 14](#_Toc140147382)

[Supplementary Methods file 7. Prespecified secondary outcomes of efficacy 15](#_Toc140147383)

[Supplementary Methods file 8. Prespecified outcomes of safety and immunogenicity 16](#_Toc140147384)

[Supplementary Methods file 9. Risk of bias assessment 17](#_Toc140147385)

[Supplementary Table S4 – Risk of bias of switching trials. 17](#_Toc140147386)

[Supplementary Methods file 10. Approximate Bayesian computation model and other approximations. 20](#_Toc140147387)

[Supplementary Methods file 11. Bayesian model fitting, model diagnostics, and estimation methods. 21](#_Toc140147388)

[Supplementary Methods file 12. GRADE-NMA. 22](#_Toc140147389)

[RESULTS 24](#_Toc140147390)

[Supplementary Results file 1. Study selection results 24](#_Toc140147391)

[Supplementary Figure S1. Flow diagram of the evidence selection process, and main reasons for exclusions. 25](#_Toc140147392)

[Supplementary Results file 2. Full study design of included trials 26](#_Toc140147393)

[Supplementary Table S5 – Full study design of included trials. 26](#_Toc140147394)

[Supplementary Results file 3. Risk of bias of included studies 29](#_Toc140147395)

[Supplementary Table S6. Full risk of bias assessment of specific domains of switching studies: domains 1, 2, and 3. 29](#_Toc140147396)

[Supplementary Table S7. Full risk of bias assessment of specific domains of switching studies: domains 4, 5, and 6. 30](#_Toc140147397)

[Supplementary Results file 4. Network map for each outcome analyzed. 31](#_Toc140147398)

[Supplementary Figure S2. Evidence network maps for secondary outcomes of efficacy: A) ACR50, B) ACR70, C) SDAI, and D) CDAI. 31](#_Toc140147399)

[Supplementary Figure S3. Evidence network maps for secondary outcomes of efficacy: A) DAS28-ESR, B) DAS28-CRP, and C) mTRSS. 33](#_Toc140147400)

[Supplementary Figure S4. Evidence network maps for secondary outcomes of safety: A) overall TEAEs, B) Serious TEAEs, C) Hypersensitivity and IRRs. 35](#_Toc140147401)

[Supplementary Figure S5. Evidence network maps for secondary outcomes of safety: A) Malignancies, B) Serious infections, and C) Overall Discontinuation rates. 36](#_Toc140147402)

[Supplementary Figure S6. Evidence network maps for secondary outcomes of immunogenicity: A) Positive anti-drug antibodies, and B) Positive neutralizing antibodies. 38](#_Toc140147403)

[Supplementary Results file 5. Efficacy outcomes: evidence synthesis 39](#_Toc140147404)

[Supplementary Table S8. Efficacy outcomes: Bayesian random-effects network meta-analyses analyzing the impact of switching or non-switching treatments of biosimilars and reference biologics considering all TNFi of interest together and by type of molecule. 39](#_Toc140147405)

[Supplementary Results file 6. Safety outcomes: evidence synthesis 44](#_Toc140147406)

[Supplementary Table S9. Safety outcomes: Bayesian random-effects network meta-analyses analyzing the impact of switching or non-switching treatments of biosimilars and reference biologics of all TNFi of interest. 44](#_Toc140147407)

[Supplementary Results file 7. Immunogenicity outcomes: evidence synthesis 46](#_Toc140147408)

[Supplementary Table S10. Immunogenicity outcomes: Bayesian random-effects network meta-analyses analyzing the impact of switching or non-switching treatments of biosimilars and reference biologics of all TNFi of interest. 46](#_Toc140147409)

[Supplementary Results file 8. Inconsistency and consistency models, and GRADE assessment 47](#_Toc140147410)

[Supplementary Table S11. Efficacy outcomes: inconsistency and consistency models, and certainty of the evidence of direct and network estimates for each pairwise comparison. 47](#_Toc140147411)

[Supplementary Table S12. Safety outcomes: inconsistency and consistency models, and certainty of the evidence of direct and network estimates for each pairwise comparison. 50](#_Toc140147412)

[Supplementary Table S13. Immunogenicity outcomes: inconsistency and consistency models, and GRADE assessment of the certainty of evidence from direct evidence and network evidence for each pairwise comparison. 52](#_Toc140147413)

[Supplementary Results file 9. Deviance information criteria 53](#_Toc140147414)

[Supplementary Table S14. Deviance information criteria values of consistency and inconsistency models. 53](#_Toc140147415)

[Supplementary Results file 10. Publication bias assessment 54](#_Toc140147416)

[Supplementary Figure S7. Comparison-adjusted funnel plot for primary outcomes: ACR 20. 54](#_Toc140147417)

[Supplementary Figure S8. Comparison-adjusted funnel plot for secondary outcomes of efficacy: A) ACR50 and B) ACR70. 55](#_Toc140147418)

[Supplementary Figure S9. Comparison-adjusted funnel plot for secondary outcomes of efficacy: DAS28-CRP. 56](#_Toc140147419)

[Supplementary Figure S10. Comparison-adjusted funnel plot for safety outcomes: A) overall TEAEs, B) serious TEAEs, C) Malignancies, and D) overall Discontinuation rates. 57](#_Toc140147420)

[Supplementary Figure S11. Comparison-adjusted funnel plot for immunogenicity outcomes: A) Positive anti-drug antibodies, and B) Positive neutralizing antibodies. 59](#_Toc140147421)

[Supplementary References file 1. References 60](#_Toc140147422)

# METHODS

# Supplementary Methods file 1. Changes to the protocol with reasons.

# Supplementary Table S1. Changes to the protocol with reasons.

| **Change** | **Protocol (original analysis/ approach)** | **Change** | **Reason** |
| --- | --- | --- | --- |
| 1 | Pairwise meta-analysis | Pairwise and Network meta-analysis | The typical switching study involves a three-arm trial in which patients in the reference biologic group are re-randomized either to continue in the biologic group or to switch to the biosimilars and in parallel, patients initially allocated to the biosimilar group continue to receive a biosimilar throughout the study period.^1-3^. A standard meta-analysis restricted to head-to-head comparisons would be not suitable for comparisons of multi-arms trials. In this case, Network meta-analysis (NMA), which is a generalization of pair-wise meta-analysis, can be used to synthesize a greater share of the available evidence and provide clinically relevant estimates to better support decision-making. ^4,5^ |
| 2 | Frequentist model | Bayesian model | We adopted the Bayesian framework because of its flexibility in terms of statistical inference. ^6^ Bayesian modelling facilitates clinical interpretability by readily calculating the posterior probability of equivalence (i.e., the probability that the posterior summary estimate lies within the prespecified equivalence margins). |
| 3 | Subgroup analysis only for primary outcomes (type of molecule) | Subgroup analysis for all outcomes of efficacy | We decided to perform subgroup analyses by type of molecule as we assessed together three molecules. This way, we verified if the effects of each molecule would change the magnitude or direction of the main conclusions. Also, these data are relevant for clinicians and patients. |
| 4 | Subgroup analysis for primary outcomes  (/switching studies vs. transitional studies) | Not perform | We did not perform the subgroup analysis by switching designs because 15 of 17 trials had a transitional study design. |
| 5 | GRADE | GRADE imprecision based on minimal clinically important differences (MCID) | GRADE working group recommends using thresholds to judge the imprecision criteria. In our protocol, we defined MCID only as primary and co-primary outcomes. But we rated the certainty of all outcomes, so we defined the MCID for all outcomes to guide the reviewers in judging the imprecision of the confidence estimates. |

# Supplementary Methods file 2 – PRISMA-NMA checklist.

#

# Supplementary Table S2 – PRISMA-NMA checklist.

| **Section/Topic** | **Item #** | **Checklist Item** | **Reported on Page #** |
| --- | --- | --- | --- |
| **TITLE** |  |  |  |
| Title | 1 | Identify the report as a systematic review *incorporating a network meta-analysis (or related form of meta-analysis).* | ***1*** |
|  |  |  |  |
| **ABSTRACT** |  |  |  |
| Structured summary | 2 | Provide a structured summary including, as applicable:  **Background:** main objectives  **Methods:** data sources; study eligibility criteria, participants, and interventions; study appraisal; and *synthesis methods, such as network meta-analysis.*  **Results:** number of studies and participants identified; summary estimates with corresponding confidence/credible intervals; *treatment rankings may also be discussed. Authors may choose to summarize pairwise comparisons against a chosen treatment included in their analyses for brevity.*  **Discussion/Conclusions:** limitations; conclusions and implications of findings.  **Other:** primary source of funding; systematic review registration number with registry name. | 2 and 3 |
|  |  |  |  |
| **INTRODUCTION** |  |  |  |
| Rationale | 3 | Describe the rationale for the review in the context of what is already known*, including mention of why a network meta-analysis has been conducted.* | ***4*** |
| Objectives | 4 | Provide an explicit statement of questions being addressed, with reference to participants, interventions, comparisons, outcomes, and study design (PICOS). | 4 |
|  |  |  |  |
| **METHODS** |  |  |  |
| Protocol and registration | 5 | Indicate whether a review protocol exists and if and where it can be accessed (e.g., Web address); and, if available, provide registration information, including registration number. | 5 |
| Eligibility criteria | 6 | Specify study characteristics (e.g., PICOS, length of follow-up) and report characteristics (e.g., years considered, language, publication status) used as criteria for eligibility, giving rationale. *Clearly describe eligible treatments included in the treatment network, and note whether any have been clustered or merged into the same node (with justification).* | 5,6 and web-appendix 3 |
| Information sources | 7 | Describe all information sources (e.g., databases with dates of coverage, contact with study authors to identify additional studies) in the search and date last searched. | 5 and web-appendix 4 |
| Search | 8 | Present full electronic search strategy for at least one database, including any limits used, such that it could be repeated. | 5 and web-appendix 4 |
| Study selection | 9 | State the process for selecting studies (i.e., screening, eligibility, included in systematic review, and, if applicable, included in the meta-analysis). | 5 and web-appendix 4 |
| Data collection process | 10 | Describe method of data extraction from reports (e.g., piloted forms, independently, in duplicate) and any processes for obtaining and confirming data from investigators. | 5 and web-appendix 5 |
| Data items | 11 | List and define all variables for which data were sought (e.g., PICOS, funding sources) and any assumptions and simplifications made. | 6 and Web-appendix 5 |
| **Geometry of the network** | **S1** | Describe methods used to explore the geometry of the treatment network under study and potential biases related to it. This should include how the evidence base has been graphically summarized for presentation, and what characteristics were compiled and used to describe the evidence base to readers. | **9 and 10** |
| Risk of bias within individual studies | 12 | Describe methods used for assessing risk of bias of individual studies (including specification of whether this was done at the study or outcome level), and how this information is to be used in any data synthesis. | 8, 9 and web-appendix 9 |
| Summary measures | 13 | State the principal summary measures (e.g., risk ratio, difference in means). *Also describe the use of additional summary measures assessed, such as treatment rankings and surface under the cumulative ranking curve (SUCRA) values, as well as modified approaches used to present summary findings from meta-analyses.* | 10 to 12, and web-appendix 10 and 11 |
| Planned methods of analysis | 14 | Describe the methods of handling data and combining results of studies for each network meta-analysis. This should include, but not be limited to:   - *Handling of multi-arm trials;* - *Selection of variance structure;* - *Selection of prior distributions in Bayesian analyses; and* - *Assessment of model fit.* | 10 to 12, and web-appendix 10 and 11 |
| **Assessment of Inconsistency** | **S2** | Describe the statistical methods used to evaluate the agreement of direct and indirect evidence in the treatment network(s) studied. Describe efforts taken to address its presence when found. | 11 |
| Risk of bias across studies | 15 | Specify any assessment of risk of bias that may affect the cumulative evidence (e.g., publication bias, selective reporting within studies). | 11 |
| Additional analyses | 16 | Describe methods of additional analyses if done, indicating which were pre-specified. This may include, but not be limited to, the following:   - Sensitivity or subgroup analyses; - Meta-regression analyses; - *Alternative formulations of the treatment network; and* - *Use of alternative prior distributions for Bayesian analyses (if applicable).* | *11* |
|  |  |  |  |
| **RESULTS†** |  |  |  |
| Study selection | 17 | Give numbers of studies screened, assessed for eligibility, and included in the review, with reasons for exclusions at each stage, ideally with a flow diagram. | 12 and web-appendix 13 |
| **Presentation of network structure** | **S3** | Provide a network graph of the included studies to enable visualization of the geometry of the treatment network. | Figure 2 and web appendix 16 |
| **Summary of network geometry** | **S4** | Provide a brief overview of characteristics of the treatment network. This may include commentary on the abundance of trials and randomized patients for the different interventions and pairwise comparisons in the network, gaps of evidence in the treatment network, and potential biases reflected by the network structure. | 14, and 15 |
| Study characteristics | 18 | For each study, present characteristics for which data were extracted (e.g., study size, PICOS, follow-up period) and provide the citations. | 12 and 13 |
| Risk of bias within studies | 19 | Present data on risk of bias of each study and, if available, any outcome level assessment. | 14 |
| Results of individual studies | 20 | For all outcomes considered (benefits or harms), present, for each study: 1) simple summary data for each intervention group, and 2) effect estimates and confidence intervals. *Modified approaches may be needed to deal with information from larger networks.* | Not presented because of the volume of outcomes and comparison data. |
| Synthesis of results | 21 | Present results of each meta-analysis done, including confidence/credible intervals. *In larger networks, authors may focus on comparisons versus a particular comparator (e.g. placebo or standard care), with full findings presented in an appendix. League tables and forest plots may be considered to summarize pairwise comparisons.* If additional summary measures were explored (such as treatment rankings), these should also be presented. | 15 to 17, tables 2 to 4, and web appendix 17 to 20 |
| **Exploration for inconsistency** | **S5** | Describe results from investigations of inconsistency. This may include such information as measures of model fit to compare consistency and inconsistency models, *P* values from statistical tests, or summary of inconsistency estimates from different parts of the treatment network. | 17 and web appendix 17 to 21 |
| Risk of bias across studies | 22 | Present results of any assessment of risk of bias across studies for the evidence base being studied. | 18 and web appendix 21 |
| Results of additional analyses | 23 | Give results of additional analyses, if done (e.g., sensitivity or subgroup analyses, meta-regression analyses*, alternative network geometries studied, alternative choice of prior distributions for Bayesian analyses,* and so forth). | 18 and web appendix 20 |
|  |  |  |  |
| **DISCUSSION** |  |  |  |
| Summary of evidence | 24 | Summarize the main findings, including the strength of evidence for each main outcome; consider their relevance to key groups (e.g., healthcare providers, users, and policy-makers). | 18 and 19 |
| Limitations | 25 | Discuss limitations at study and outcome level (e.g., risk of bias), and at review level (e.g., incomplete retrieval of identified research, reporting bias). *Comment on the validity of the assumptions, such as transitivity and consistency. Comment on any concerns regarding network geometry (e.g., avoidance of certain comparisons).* | 20 and 21 |
| Conclusions | 26 | Provide a general interpretation of the results in the context of other evidence, and implications for future research. | 21 |
|  |  |  |  |
| **FUNDING** |  |  |  |
| Funding | 27 | Describe sources of funding for the systematic review and other support (e.g., supply of data); role of funders for the systematic review. This should also include information regarding whether funding has been received from manufacturers of treatments in the network and/or whether some of the authors are content experts with professional conflicts of interest that could affect use of treatments in the network. | ***22*** |

# Supplementary Methods file 3 – Description of the eligibility criteria and study selection

**Participants:** patients with RA that had been diagnosed with validated and established international criteria. No limitations were imposed based on age, baseline RA severity, sex, lines of treatment (e.g., treatment-naïve patients or second line of treatment), or any other major demographic characteristics.**Interventions and comparators:** any biosimilars of adalimumab, etanercept, and infliximab. Comparators of interest were the reference biologic drugs (i.e., adalimumab, etanercept, and infliximab originals). No restrictions were imposed on dosages, treatment schedules, co-treatment, or combined therapies. We chose these three main biologics because they belong to the same drug class of tumour necrosis factor inhibitors (TNFIs) with comparative safety and effectiveness in the management of RA.^7^ Also, these three disease-modifying antirheumatic drugs are the most prescribed first-line biologic therapy and have the highest number of approved biosimilars for RA in the market. ^8,9^

**Type of study:**

We included randomized controlled trials (RCT) with two-or-multiple-part designs. The following four main designs of switching trials were considered.^1^

*Single-switch design:* Trials in which there is a single switch from each treatment to the other. All patients receive the study interventions in successive periods. Firstly, patients are randomly allocated to either a biosimilar or a biologic drug (first period). Then, in the second period, treatments are randomly switched in both directions (group 1: biologic → biosimilar; group 2: biosimilar → biologic OR group 1: biologic → biosimilar, group 2: biosimilar → biologic; group 3: biologic → biologic; group 4: biosimilar → biosimilar).

*Transition design 1* (two non-switching groups as a control): Trials in which there is a single switch from one treatment (biologic drug) to another (biosimilar drug), but not the contrary. Firstly, patients are randomly allocated to either a biosimilar or a biologic drug (first period). Then, in the second period, the trial becomes a three-arm trial in which patients in the biologic drug group are re-randomized either to continue in the biologic group or to switch to the biosimilar drug treatment. Patients initially allocated to the biosimilar group continue to receive a biosimilar throughout the study period (experimental group: biologic → biosimilar; control arm 1: biologic → biologic; control arm 2: biosimilar → biosimilar).

*Transition design 2* (randomized trials with an open-label extension; single non-switching group as a control): Trials in which there is a single switch from a biologic drug to a biosimilar drug, but not the contrary. Firstly, patients are randomly allocated to either a biosimilar or a biologic drug (first period). Then, in the open-label extended phase (second period), all patients (intervention and control groups) receive the biosimilar drug (experimental group: biologic → biosimilar; control arm 1: biosimilar → biosimilar).

*Multiple switches design*: Also known as interchangeability design, in which multiple switches between treatments are allowed throughout the trial follow-up.

**Screening and selection process**

We used a customized web platform for data extraction and curation using Ragic (www.ragic.com). This database was carefully designed to simultaneously allow for study screening and selection and data extraction for the systematic review. Two independent investigators conducted fully independently all steps. Specifically, during the screening phase, two reviewers evaluated titles and abstracts, and disagreements were solved by a consensus. After that, for each study selected, full-length articles were downloaded, and then the eligibility criteria of pre-selected trials were re-assessed by the two reviewers and a third reviewer was consulted in case of discordances. The main reasons to exclude were categorized and reported.

# Supplementary Methods file 4 – Evidence sources and search strategy

**Evidence sources**

Previously, we published a detailed description of evidence sources and search strategy in our protocol^1^, and we reproduce it here (Supplementary Methods 4 Table S3). The search strategies were initially run from database inception until 07 September 2021 using the following electronic databases: MEDLINE via PubMed, EMBASE, Cochrane Central Register of Controlled Trials (CENTRAL), and Latin American and Caribbean Health Science (LILACS). We searched for non-published or ongoing trials in the main register databases of clinical trials, the EU Clinical Trial Register (https://www.clinicaltrialsregister.eu), International Clinical Trials Registry Platform-World Health Organization (http://apps.who.int/trialsearch/), and Clinicaltrials (https://clinicaltrials.gov/). Also, we manually screen the references of all included trials as well as previous systematic reviews. A citation search was done in Google Scholar and Epistemonikos (https://www.epistemonikos.org/) to retrieve relevant reports citing all relevant included articles. No language limitation was imposed.

# Supplementary Table S3 – Evidence sources and search strategy

| **Database (molecule)** | **Search Strategy** |
| --- | --- |
| PubMed -etanercept | ((((((((("Arthritis, Rheumatoid"[Mesh]) OR Rheumatoid Arthritis)) OR ((((("Arthritis, Juvenile"[Mesh]) OR "Rheumatoid Arthritis, Systemic Juvenile" [Supplementary Concept]) OR Juvenile Arthritis) OR Arthritis, Juvenile Chronic) OR Arthritis, Juvenile Idiopathic)))))) AND ((((((((((("Biosimilar Pharmaceuticals"[Mesh]) OR Pharmaceuticals, Biosimilar) OR Follow-on Biologics) OR Biologics, Follow-on) OR Follow on Biologics) OR Subsequent Entry Biologics) OR Biologics, Subsequent Entry) OR Biosimilars)))) AND (((((((((("Etanercept"[Mesh]) OR TNFR-Fc Fusion Protein) OR Recombinant Human Dimeric TNF Receptor Type II IgG Fusion Protein) OR TNF Receptor Type II IgG Fusion Protein) OR Erelzi) OR ("GP2015" [Supplementary Concept] OR "LBEC0101" [Supplementary Concept]))) OR benepali) OR etanercept szzs))) |
| PubMed - infliximab | (((((((((((("Arthritis, Rheumatoid"[Mesh]) OR Rheumatoid Arthritis)) OR ((((("Arthritis, Juvenile"[Mesh]) OR "Rheumatoid Arthritis, Systemic Juvenile" [Supplementary Concept]) OR Juvenile Arthritis) OR Arthritis, Juvenile Chronic) OR Arthritis, Juvenile Idiopathic))))))))) AND ((((((((((((((("Biosimilar Pharmaceuticals"[Mesh]) OR Pharmaceuticals, Biosimilar) OR Follow-on Biologics) OR Biologics, Follow-on) OR Follow on Biologics) OR Subsequent Entry Biologics) OR Biologics, Subsequent Entry) OR Biosimilars))))))) AND (((((((("Infliximab"[Mesh])) OR "SB2 infliximab" [Supplementary Concept]) OR "GP1111" [Supplementary Concept])) OR "CT-P13" [Supplementary Concept])) OR (Infliximab-abda[Text Word]) OR Infliximab-dyyb[Text Word]) OR Infliximab-qbtx[Text Word]) OR Infliximab-axxq[Text Word]) OR Inflectra[Text Word]) OR Renflexis[Text Word]) OR Ixifi[Text Word]) OR Zessly[Text Word]) OR Flixabi) OR Remsima[Text Word]))) |
| PubMed - adalimumab | (((((((((((("Arthritis, Rheumatoid"[Mesh]) OR Rheumatoid Arthritis)) OR ((((("Arthritis, Juvenile"[Mesh]) OR "Rheumatoid Arthritis, Systemic Juvenile" [Supplementary Concept]) OR Juvenile Arthritis) OR Arthritis, Juvenile Chronic) OR Arthritis, Juvenile Idiopathic))))))))) AND ((((((((((((((("Biosimilar Pharmaceuticals"[Mesh]) OR Pharmaceuticals, Biosimilar) OR Follow-on Biologics) OR Biologics, Follow-on) OR Follow on Biologics) OR Subsequent Entry Biologics) OR Biologics, Subsequent Entry) OR Biosimilars))))))) AND ((((((((((((((((((((("Adalimumab"[Mesh]) OR D2E7 Antibody) OR Antibody, D2E7) OR Adalimumab-adbm) OR Adalimumab-atto) OR Adalimumab-adaz[Text Word]) OR Adalimumab-bwwd[Text Word]) OR Adalimumab-afzb[Text Word]) OR Amjevita[Text Word]) OR Hadlima[Text Word]) OR Cyltezo[Text Word]) OR Hyrimoz[Text Word]) OR Abrilada[Text Word]) OR Halimatoz[Text Word]) OR Hefiya[Text Word]) OR Imraldi[Text Word]) OR Hulio[Text Word]) OR Kromeya[Text Word]) OR Idacio[Text Word])) OR (((("ABP 501" [Supplementary Concept]) OR "GP2017" [Supplementary Concept]) OR "BI 695501" [Supplementary Concept]) OR "PF-06410293" [Supplementary Concept])) |
| EMBASE - etanercept | ('rheumatoid arthritis'/exp OR 'rheumatoid arthritis' OR 'juvenile rheumatoid arthritis'/exp OR 'juvenile rheumatoid arthritis') AND ('biosimilar agent'/exp OR 'biosimilar agent' OR (('biosimilar' OR 'biosimilar'/exp OR biosimilar) AND pharmaceuticals) OR ('follow on' AND ('biologics' OR 'biologics'/exp OR biologics)) OR (subsequent AND entry AND ('biologics' OR 'biologics'/exp OR biologics))) AND ('etanercept'/exp OR 'etanercept' OR 'etanercept szzs'/exp OR 'etanercept szzs' OR 'erelzi' OR 'erelzi'/exp OR erelzi OR 'benepali' OR 'benepali'/exp OR benepali OR sb4 OR 'gp2015'/exp OR gp2015) |
| EMBASE - infliximab | ('rheumatoid arthritis'/exp OR 'rheumatoid arthritis' OR 'juvenile rheumatoid arthritis'/exp OR 'juvenile rheumatoid arthritis') AND ('biosimilar agent'/exp OR 'biosimilar agent' OR (('biosimilar' OR 'biosimilar'/exp OR biosimilar) AND pharmaceuticals) OR ('follow on' AND ('biologics' OR 'biologics'/exp OR biologics)) OR (subsequent AND entry AND ('biologics' OR 'biologics'/exp OR biologics))) AND ('infliximab'/exp OR 'infliximab' OR 'inflectra' OR 'inflectra'/exp OR inflectra OR 'ixifi' OR 'ixifi'/exp OR ixifi OR 'renflexis' OR avsola OR 'zessly'/exp OR zessly OR 'remsima'/exp OR remsima OR 'renflexis'/exp OR renflexis OR 'infliximab dyyb'/exp OR 'infliximab dyyb' OR 'infliximab qbtx'/exp OR 'infliximab qbtx' OR 'infliximab axxq' OR 'infliximab abda'/exp OR 'infliximab abda' OR ctp13 OR sb2 OR 'abp710'/exp OR abp710 OR 'gp1111'/exp OR gp1111 OR 'pf06438179'/exp OR pf06438179) AND [embase]/lim |
| EMBASE - adalimumab | (('rheumatoid arthritis'/exp OR 'rheumatoid arthritis' OR 'juvenile rheumatoid arthritis'/exp OR 'juvenile rheumatoid arthritis') AND ('biosimilar agent'/exp OR 'biosimilar agent' OR (('biosimilar' OR 'biosimilar'/exp OR biosimilar) AND pharmaceuticals) OR ('follow on' AND ('biologics' OR 'biologics'/exp OR biologics)) OR (subsequent AND entry AND ('biologics' OR 'biologics'/exp OR biologics))) AND ('adalimumab'/exp OR 'adalimumab' OR 'amjevita' OR 'amjevita'/exp OR amjevita OR 'cyltezo' OR 'cyltezo'/exp OR cyltezo OR hadlima OR 'hyrimoz' OR 'hyrimoz'/exp OR hyrimoz OR 'adalimumab atto'/exp OR hefiya OR imraldi OR hulio OR kromeya OR idacio OR abrilada OR 'adalimumab atto' OR 'adalimumab adbm'/exp OR 'adalimumab adbm' OR 'adalimumab adaz'/exp OR 'adalimumab adaz') OR 'adalimumab afzb' OR abp501 OR gp2017 OR sb5 OR fkb327 OR msb11022 OR pf06410293) AND [embase]/lim |
| CENTRAL - etanercept | #1 MeSH descriptor: [Biosimilar Pharmaceuticals] explode all trees  #2 MeSH descriptor: [Etanercept] explode all trees  #3 #1 AND #2 |
| CENTRAL - infliximab | #1 MeSH descriptor: [Biosimilar Pharmaceuticals] explode all trees  #2 MeSH descriptor: [Infliximab] explode all trees  #3 #1 AND #2 |
| CENTRAL - adalimumab | #1 MeSH descriptor: [Biosimilar Pharmaceuticals] explode all trees  #2 MeSH descriptor: [Adalimumab] explode all trees  #3 #1 AND #2 |
| LILACS – etarnercept | biosimilar [Palavras] and etanercept [Palavras] |
| LILACS - infliximab | biosimilar [Palavras] and infliximab [Palavras] |
| LILACS - adalimumab | biosimilar [Palavras] and adalimumab [Palavras] |
| EU Clinical Trial Register- etanercept | biosimilar AND etanercept AND rheumatoid arthritis |
| EU Clinical Trial Register- infliximab | biosimilar AND infliximab AND rheumatoid arthritis |
| EU Clinical Trial Register- adalimumab | biosimilar AND adalimumab AND rheumatoid arthritis |
| Clinical Trial NIH- etanercept | BIOSIMILAR \| Etanercept \| rheumatoid arthritis |
| Clinical Trial NIH- infliximab | BIOSIMILAR \| Infliximab \| rheumatoid arthritis |
| Clinical Trial NIH- adalimumab | BIOSIMILAR \| Adalimumab \| rheumatoid arthritis |
| ICTRP- etanercept | biosimilar AND etanercept AND rheumatoid arthritis |
| ICTRP- infliximab | biosimilar AND infliximab AND rheumatoid arthritis |
| ICTRP- adalimumab | biosimilar AND adalimumab AND rheumatoid arthritis |

# Supplementary Methods file 5. Data collection process

Whenever available, we collected the population per-protocol (PP) data for our analysis because this approach frequently results in narrower confidence intervals than the intention-to-treat analysis and can be more conservative for equivalence testing. ^2,10,11^

Two investigators extracted all data independently, and discrepancies sound was solved via a consensus or consultation with a third reviewer. Further details on data extraction management were given in our protocol. ^1^

In each phase of the trial, we identified all types of biosimilars with their specific trial registry name. Later, we categorized all interventions in a general designation and abbreviation format. For example, CT-P13, a biosimilar was referred to as biosimilar (Bios), and their reference, infliximab, was like reference (Ref).

The following categories of information were obtained from the included trials: (i) Study details; (ii) Baseline characteristics of participants; and (iii) Study outcomes variable (continuous and binary).

1. Study details characteristics: year of publication, sample size, trial design characteristics, and trial duration (time from randomization until end of follow-up) per phase of the study, i.e., efficacy and switching phase, study registration details.
2. Baseline characteristics of participants: demographic characteristics (age, sex), clinical characteristics (RA severity, Factor Rheumatoid positive, etc), and characteristics of treatments (dosage, frequency of administration, and adjuvant therapy).
3. Outcomes: all outcomes were prespecified in the registered PROSPERO synopses and were categorized into three types: efficacy (encompassing outcomes related to disease activity, functional capacity, quality of life, and structural damage progression), safety, and immunogenicity. For efficacy outcomes, we extracted data from the time points closest to 6 months after the first switching. For safety and immunogenicity outcomes, we collected data from the longest follow-up available in the first switching phase. For continuous outcomes, we extracted as mean, standard deviations, median, interval interquartile 25% and 75%, minimum and maximum, n total participants. These values were extracted from baseline, follow up and change from baseline. For binary outcomes, the number of patients with the event and the number total of participants across treatments were extracted.

# Supplementary Methods file 6. Prespecified primary and co-primary outcomes of efficacy

The prespecified primary of efficacy was the treatment success at 6 months after the first switch according to the American College of Rheumatology 20% response criteria (ACR20), which measures the clinical response in RA trials. ^12^ We also prespecified The Health Assessment Questionnaire - Disability Index (HAQ-DI) change from baseline to 6 months after the first switching as a co-primary outcome, which measures functional disability based on patient-reported outcomes. ^13^ The decision to have a primary and co-primary outcome was based on the need to address different response domains to therapy. ^1^ While ACR20 captures the disease severity change over time (from a clinical perspective), HAQ-DI measures the self-perceived disability, directly incorporating the patient's perceived benefits from the treatment. There is compelling evidence indicating that clinical responses and patient-perceived benefits of therapy should be considered jointly when inferring the efficacy of biological therapies in patients with RA. ^14-16^

The ACR20 requires at least a 20% improvement in the core set measures for a patient to reach improvement. ^12^ Specifically, a participant was a responder if the following 3 criteria for improvement from baseline were met: ≥ 20% improvement in TJC; ≥ 20% improvement in SJC; and ≥ 20% improvement in at least 3 of the 5 following parameters: Patient's assessment of pain (VAS 0 a 100 mm); Patient's global assessment of disease activity (Likert scale from 0 to 10); Physician's global assessment of disease activity (Likert scale from 0 to 10); Patient's self-assessment of physical function (HAQ-DI); CRP level. ACR20 was summarised as relative risk (RR) with an RR greater than 1.0, indicating a higher response probability with biosimilar drugs than in reference biologics.

The co-primary outcome HAQ-DI assesses the functional status of patients through the evaluation of eight domains of daily life activities. The highest score reported for any component question in each domain determines the final score for that domain. By convention, the overall disability index is expressed on a 0 to 3 scale, representing an average score across the domains. A HAQ-DI of 0 indicates no functional disability, whereas a HAQ-DI of 3 denotes severe functional disability. ^13^ HAQ-DI was presented as the standardised mean differences (SMD, Cohen's effect size), and an SMD smaller than 0 indicates a better outcome for biosimilar drugs than in reference biologics.

# Supplementary Methods file 7. Prespecified secondary outcomes of efficacy

The following prespecified secondary outcomes of efficacy were assessed 6 months after the first switching. We also included a range of clinical and patient-reported outcomes englobing disease activity measures, functional capacity/ quality of life, and structural damage as follows:

Measures of disease activity:

1. Clinical outcomes (binary): the American College of Rheumatology criteria with 50% (ACR50) and 70% (ACR70) responses, European League Against Rheumatism (EULAR) good response versus nonresponse, EULAR moderate response versus non-response. These outcomes were summarised as RR and with an RR greater than 1.0, indicating a higher response probability with biosimilar drugs than in reference biologics.
2. Patient-reported outcomes (continuous): Simplified disease activity score (SDAI), clinical disease activity score (CDAI), disease activity score in 28 joints based on the erythrocyte sedimentation rate (DAS28-ESR), disease activity score in 28 joints with four components based on C-reactive protein (DAS28-CRP). These outcomes were presented as SMD and SMD smaller than 0 indicates a better outcome for biosimilar drugs than in reference biologics.

Functional capacity/quality of life:

1. Patient-reported outcomes (continuous): the Medical Outcomes Study 36-item Short-Form Health Survey (SF-36) (physical and mental components summaries). These outcomes were presented as SMD, and an SMD smaller than 0 indicates a better outcome for biosimilar drugs than in reference biologics.

Prevention of structural damage progression:

1. Clinical outcomes (continuous): scores of Sharp/ van der Heijde or Sharp-van der Heidje Modified Score Method (mTRSS). This outcome was presented as SMD, and an SMD smaller than 0 indicates a better outcome for biosimilar drugs than in reference biologics.

# Supplementary Methods file 8. Prespecified outcomes of safety and immunogenicity

The prespecified safety outcomes included: the proportion of patients with treatment-emergent adverse events (TEAEs), serious TEAEs, all-cause mortality, treatment-related mortality, overall mortality, and special adverse events of interest including infusion-related reactions (IRRs), injection site reactions (ISRs), hypersensitivity, malignancies, active tuberculosis, and serious infections. We also assessed the overall discontinuation rates (dropouts). Those outcomes were calculated as RR and a RR greater than 1.0 indicates that the risk of the outcome is increased by the biosimilar drugs compared to reference biologics.

For immunogenicity outcomes, we assessed the proportion of patients with positive anti-drug antibodies (ADAs), and the proportion of patients with ADAs who developed positive neutralizing antibodies (Nabs). Both outcomes were summarised as RR and a RR greater than 1.0 indicates that the risk of the outcome is increased by the biosimilar drugs compared to reference biologics.

# Supplementary Methods file 9. Risk of bias assessment

Two review authors independently assessed the risk of bias in the included studies. For switching trials, we used the recommendations of Moots et al.^17^ and the Food Drug Administration guidance^2^ to assess the risk of bias across six specifics domains: 1) Randomized and blinded design with appropriate control arms; 2) At least 1-way switch from originator to a biosimilar; 3) The assessment of immunogenicity; 4) The washout period between treatment; 5) Enough power to assess efficacy and safety (equivalence phase), and 6) Enough follow-up periods

We rated each domain of risk of bias as being at a low, unclear, or high risk of bias. If the trial has one or more domains with a high risk of bias, we considered it a high-risk-of-bias study. If the trial has more than two domains at uncertain risk of bias, we judged the risk of bias to be uncertain. If the trial has a low risk of bias in all domains or one domain had an uncertain bias, it was considered a low-risk-of-bias study. Detailed information about the criteria for the judgement is reproduced here from our protocol.^1^ We use *robvis* tool to create risk-of-bias plots^18^.

# Supplementary Table S4 – Risk of bias of switching trials.

| **Domain** | **Criteria to identify bias** | **Description/ Judgment** |
| --- | --- | --- |
| **Domain 1 – The randomized and blinded design with appropriate control arm** | There was a randomization step before the switch? | Yes, No, or Unclear |
|  | Did they keep the switching period blinded? | Yes, No, or Unclear |
|  | The study population was selected for a positive response or less disease severity? | Yes, No, or Unclear |
|  | What is your judgment about the randomized and blinded design with appropriate control arms? | **Low risk of bias:** There was a randomization step before the switch; They kept the switching period blinded; The study population was not selected for a positive response or less disease severity.  **High risk of bias:** There is no randomization step before the switch; It was an open-label period; The study population was selected for a positive response or less disease severity.  **Unclear risk of bias:** Insufficient information to permit judgment of ‘Low risk’ or ‘High risk’. |
| **Domain 2 – The number and way of switching** | The study had at least 1 arm incorporating switching between the proposed interchangeable product and the reference. | Yes, No, or Unclear |
|  | What is your judgment about the number and way of switching? | **Low risk of bias:** At least 1 arm incorporates switching between the proposed interchangeable product and the reference product, whereas the other arm remains on the reference product.  **High risk of bias:** The 1 arm incorporating switching between the proposed interchangeable product and the reference product was not available.  **Unclear risk of bias:** Insufficient information to permit judgment of ‘Low risk’ or ‘High risk’. |
| **Domain 3 – The assessment of immunogenicity** | Immunogenicity was adequately measured in both switching and non-switching arms. | Yes, No, or Unclear |
|  | Immunogenicity was measured for enough time (more than 12 months). | Yes, No, or Unclear |
|  | What is your judgment about the assessment of immunogenicity? | **Low risk of bias:** Immunogenicity was adequately measured in both switching and non-switching arms; Immunogenicity was measured for sufficient time (more than 12 months).  **High risk of bias:** Immunogenicity was not measured in both switching and non-switching arms; Immunogenicity was not measured for sufficient time (less than 12 months).  **Unclear risk of bias:** Insufficient information to permit judgment of ‘Low risk’ or ‘High risk’. |
| **Domain 4 – The washout period between treatment** | Had the study a wash-period before the switch? * | Yes, No, or Unclear |
|  | If yes, the washout was done in enough time? ** | Yes, No, or Unclear |
|  | What is your judgment about the washout period between treatments (multiple switching)? | **Low risk of bias:** There is an enough and appropriate wash-out period before switching  **High risk of bias:** There is no wash-out period before switching  **Unclear risk of bias:** Insufficient information to permit judgment of ‘Low risk’ or ‘High risk’. |
| **Domain 5 – Enough power to assess efficacy and safety (equivalence studies)** | Had the study a small number (<50) of patients in the switch groups? | Yes, No, or Unclear |
|  | Was there a high rate of differential loss of participants before switching? | Yes, No, or Unclear |
|  | Was the study powered to assess efficacy in individual diseases? | Yes, No, or Unclear |
|  | What is your judgment about enough power to assess efficacy and safety (equivalence studies)? | **Low risk of bias:** Statistical power was enough, i.e. There are many patients in the switch groups.  There was a low or similar rate of loss of participants before switching;  The study was powered to assess efficacy in individual diseases.  **High risk of bias:** Statistical power was limited because of the small patient numbers in the switch groups;  There was a high or differential loss of participants before switching; The study was NOT powered to assess efficacy in individual diseases.  **Unclear risk of bias:** Insufficient information to permit judgment of ‘Low risk’ or ‘High risk’. |
| **Domain 6 – An enough follow-up period** | The follow-up period after a switch was sufficiently long to allow the detection of clinically relevant differences (equal to or less than 24 weeks)? | Yes, No, or Unclear |
|  | What is your judgment about enough follow-up period? | **Low risk of bias:** The follow-up period after a switch was sufficiently long to allow the detection of clinically relevant differences (More than 24 weeks)  **High risk of bias:** The follow-up period after a switch was not sufficiently long to allow the detection of clinically relevant differences (equal to or less than 24 weeks)  **Unclear risk of bias:** Insufficient information to permit judgment of ‘Low risk’ or ‘High risk’. |

# Supplementary Methods file 10. Approximate Bayesian computation model and other approximations.

We used an Approximate Bayesian Computation (ABC) model ^19^ to estimate mean and standard deviations based on available summary statistics.

# Supplementary Methods file 11. Bayesian model fitting, model diagnostics, and estimation methods.

Model parameters were estimated by Markov chain Monte Carlo simulations (Gibbs sampling). Models were fitted with three chains, a burn-in period of 250,000 simulations, and 166,667 additional simulations (totalizing 500,000 iterations). We checked convergence by examining trace plots and the Gelman–Rubin statistic. Auto-correlation plots were used to check auto-correlation.

# Supplementary Methods file 12. GRADE-NMA.

Two reviewers assessed the overall certainty of the evidence, and disagreements were settled mostly by consensus and discussed with a third reviewer when needed. The Grading of Recommendations Assessment, Development, and Evaluation (GRADE) four-step approach was used for rating the quality of treatment effect estimates from the Network meta-analysis (NMA) of each outcome. ^20-22^This approach consists of: (1) presenting the direct and indirect estimates of effect for the pairwise comparison, (2) rating the certainty of both direct and indirect estimates, (3) presenting the NMA estimate for each comparison of the evidence network., and (4) rating the certainty of each NMA, based on the ratings of the direct and indirect estimates and the assessment of coherence (i.e., the extent of similarity of direct and indirect estimates). In the most recent advances of GRADE for NMA ^21^ is stated that there is no need to rate the indirect evidence when the certainty of the direct evidence is high, and the contribution of the direct evidence to the network estimate is at least as great as that of the indirect evidence.

Our results showed that the credible interval or confidence intervals of the NMA estimates were mostly more precise than the direct estimates. Then, we assume that the direct estimates are contributing greater to the network evidence. Furthermore, the network evidence is derived from multi-arm trials and closed-loops networks. For these reasons, we did not present or rate the indirect evidence, consequently, there is no need to assess the coherence criteria. We applied the conventional criteria of study limitation, inconsistency, indirectness, and publication bias for rating the confidence estimates for the direct comparisons. We rated the quality of NMA based on direct quality rating, and the imprecision of the NMA estimates. Our judgement is based on the primary analysis of Bayesian random-NMA, the width of the Bayesian Credible Intervals (CrIs) around the effect estimate, the magnitude of the effect estimate, and the overlap of the %95 CrIs in the equivalence margins based on the minimum clinically important difference (MICD), sample size and the number of events. We also verified the confidence of NMA estimates with sensitivity analysis using a fixed-effect model. The rationale for estimating the margins of equivalence or MCID is described elsewhere.^1,23^ We rated the certainty of evidence as very low, low, moderate, or high. We present our results and certainty assessment based on recommendations of GRADE for NMA.^20,21^

**DIRECT EVIDENCE**

1. **Study limitations (Risk of bias)**:

We downgraded in cases in which one or more risk of bias domains was high, or several domains were unclear.

1. **Inconsistency**

We downgraded if:

- wide variance of point estimates across studies with minimal or no overlap of CrIs.
- I^2^ was higher than 50% and analysis of between-study variance τ^2^ indicated possibly moderate to high heterogeneity.

1. **Indirectness**

We downgraded if:

- there were differences regarding the study population or when the time of the outcome assessments differed.
- subgroup analysis detected important differences in the direction of effect estimates.

1. **Publication bias**

We performed a visual inspection on comparison-adjusted funnel plots for each outcome. Additionally, we also assessed the comparison-adjusted funnel plots for each pairwise comparison separately, but the plots were not shown because of the number of comparisons.

We downgraded if:

- Asymmetry of the funnel plot was suspected.

**NETWORK EVIDENCE**

1. **Imprecision:**

We downgraded if:

- the CrIs of effects estimates were wide, and when the CrIs crossed the equivalence margins based on the MCDI. Our previous publications ^1,23^ give a detailed description of the methods for estimating the thresholds for the MCDI of each outcome. The following MCDI were considered:

Primary and co-primary outcomes

- - - ACR20: [RR 0.94, 1.04]
    - HAQ-DI: [-0.15, 0.15 units of original scale] or [SMD -0.22, 0.22]

Secondary outcomes

- ACR50, ACR70: [RR 0.75, 1.25]
- SDAI: [-12, 12 units of original scale] or [SMD -0.80, 0.80].
- CDAI: [-13, 13 units of original scale] or [SMD -0.80, 0.80].
- DAS28-ESR: [-1.2, 1.2 units of original scale] or [SMD -0.74, 0.74]
- DAS28-CRP: [-1.0, 1.0 unit of original scale] or [-0.61, 0.61]
- mTRSS: [-5, 5 units of original scale] or [-0.60, 0.60]
- Safety outcomes: the number of events was less than 400, and [RR 0.75, 1.25]
- Immunogenicity outcomes: the number of events was less than 400, and [RR 0.75, 1.25]
- when CrIs from Bayesian estimates and confidence interval (CI) from the frequentist approach did not overlap.
- In the case of NMA of sparse networks, when the CrIs of direct evidence was narrow, but Bayesian NMA was wider, we downgraded in case of the presence of wide intervals also in the estimates using a frequentist framework and fixed-effects models.

We upgraded if:

- in the case of CrIs from NMA are narrower than those from direct evidence, and the interval falls into the thresholds for the minimal important difference.
- in the case of wider CrIs from NMA, but the estimates from direct evidence were narrow and the frequentist and fixed-effect estimates fell into the minimal important difference thresholds.

# RESULTS

# Supplementary Results file 1. Study selection results

Up to April 2021, two reviewers independently screened 2023 titles and abstracts records. Of these, 131 full-text articles were downloaded and assessed for full eligibility criteria. In total, 25 RCTs ^24-71^ were eligible for answering the first objective of the systematic review focusing on efficacy, safety, and immunogenicity between biosimilars and reference biologics in patients with moderate to severe RA.^23^ Among these, six RCTs ^64-69^ did not perform a switching phase and in two^70,71^ the switching phase data was not available. Therefore, 17 trials ^24-63^ with 2-part study designs including a switching phase were eligible to answer the objective of the present systematic review focusing on the impact of switching between treatments. The evidence flow diagram of the study selection process is provided in **Supplementary Figure S1.**


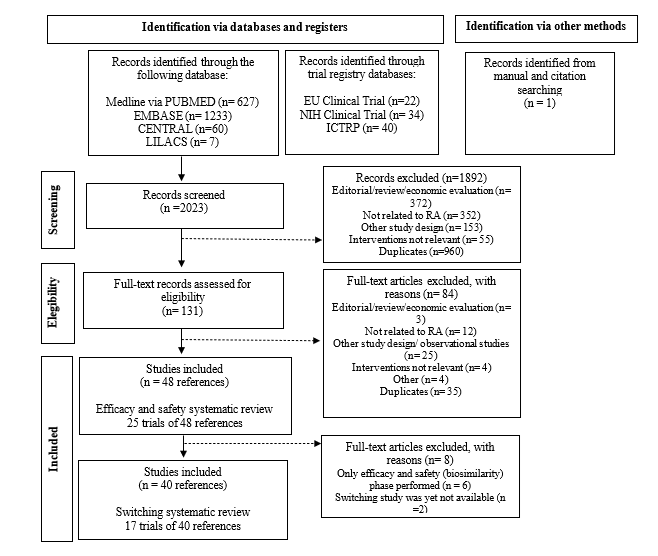


# Supplementary Figure S1. Flow diagram of the evidence selection process, and main reasons for exclusions.

CENTRAL: Cochrane Central Register of Controlled Trials; LILACS: Latin American and Caribbean Health Science; EU: European; NIH: National Institutes of Health; ICTRP: International Clinical Trials Registry Platform-World Health Organization; RA: rheumatoid arthritis.

# Supplementary Results file 2. Full study design of included trials

# Supplementary Table S5 – Full study design of included trials.

|  | **Efficacy and safety phase** | | **First switching** | | **Second switching** | | **Full trial** |
| --- | --- | --- | --- | --- | --- | --- | --- |
| **Author, year / Study information** | **Arms (n)** | **Design (follow-up)** | **Arms (n)** | **Design (follow-up)** | **Arms** | **Design (follow-up)** | **Follow-up** |
| **Alten, 2017**  ^24-30^  NCT02260791 and NCT02405780 | FKB327 (366) | RCT of EQ (24) | FKB327/FKB327 (216) | Single switch (30) | FKB327/FKB327/FKB327 (189) | Transitional design 3 (50) | 104 |
|  |  |  | FKB327/ADA (108) |  | FKB327/ADA/FKB327 (100) |  |  |
|  | ADA (362) |  | ADA/ADA (213) |  | ADA/ADA/FKB327 (190) |  |  |
|  |  |  | ADA/FKB327 (108) |  | ADA/FKB327/FKB327 (93) |  |  |
| **Cohen, 2017** ^31,32^  NCT01970475 and NCT02114931 | ABP-501 (264) | RCT of EQ (26) | ABP-501/ABP-501 (230) | Transitional design 2 (72) | -- | -- | 98 |
|  | ADA (262) |  | ADA/ABP-501 (237) |  |  |  |  |
| **Fleishmann, 2018** ^33-35^  NCT02480153 | PF-06410293 (297) | RCT of EQ (26) | PF-06410293/PF-06410293 (283) | Transitional design 1 (26) | PF-06410293/PF-06410293/PF-06410293 (259) | Transitional design 3 (40) | 92 |
|  | ADA (300) |  | ADA/ADA (135) |  | ADA/ADA/PF-06410293 (121) |  |  |
|  |  |  | ADA/PF-06410293 (134) |  | ADA/PF-06410293/PF-06410293 (127) |  |  |
| **Cohen, 2018** ^36,37^  NCT02137226 and NCT02640612 | BI-695501 (324) | RCT of EQ (24) | .BI-695501/BI-695501 (298) | Transitional design 1 (34) | BI-695501/BI-695501/BI-695501 (225) | Transitional design 3 (48) | 106 |
|  | ADA (321) |  | ADA/ADA (148) |  | ADA/ADA/BI_695501 (103) |  |  |
|  |  |  | ADA/BI-695501 (147) |  | ADA/BI_695501/BI_695501 (102) |  |  |
| **Weinblatt, 2018** ^38,39^  NCT02167139 | SB5 (271) | RCT of EQ (24) | SB5/SB5 (254) | Transitional design 1 (28) | -- | -- | 52 |
|  | ADA (273) |  | ADA/ADA (129) |  | -- | -- |  |
|  |  |  | ADA/SB5 (125) |  |  |  |  |
| **Willand, 2019** ^40,41^  NCT02744755 | GP2017 (177) | RCT of EQ (24) | GP2017/GP2017 (159) | Transitional design 2 (24) | -- | -- | 48 |
|  | ADA (176) |  | ADA/GP2017 (166) |  |  |  |  |
| **Kay, 2021** ^42,43^  NCT03789292 | CT-P17 (324) | RCT of EQ (24) | CT-P17/CT-P17 (303) | Transitional design 1 (28) | -- | -- | 52 |
|  | ADA (324) |  | ADA/ADA (153) |  |  |  |  |
|  |  |  | ADA/CT_P17 (151) |  |  |  |  |
| **Emery, 2015** ^44-46^  NCT01895309 | SB4 (299) | RCT of EQ (52) | SB4/SB4 (126) | Transitional design 2 (52) | -- | -- | 100 |
|  | ETN (297) |  | ETN/SB4 (119) |  |  |  |  |
| **Odell, 2016** ^47,48^  NCT02115750 | CHS-0214 (324) | RCT of EQ (24) | CHS-0214/ CHS-0214 (284) | Transitional design 2 (24) | -- | -- | 48 |
|  | ETN (323) |  | ETN/ CHS-0214(280) |  |  |  |  |
| **Matsuno, 2017** ^49,50^  NCT02357069 and NCT02715908 | LBEC0101 (187) | RCT of EQ (52) | LBEC0101/LBEC0101 (70) | Transitional design 2 (48) | -- | -- | 100 |
|  | ETN (187) |  | ETN/LBEC0101 (78) |  |  |  |  |
| **Matucci-Cerinic, 2018**  ^51,52^  NCT02638259 | GP2015  (186) | RCT of EQ (24) | GP2015/GP2015  (175) | Transitional design 2 (24) | -- | -- | 48 |
|  | ETN  (190) |  | ETN/GP2015  (166) |  |  |  |  |
| **Yamanaka, 2020**  ^53^  2015-002809-12 | YLB113 (266) | RCT of EQ (56) | YLB113/ETN (10) | Single switch 2 (28) | -- | -- | 56 |
|  | ETN (262) |  | ETN/YLB113 (8) |  |  |  |  |
| **Yoo, 2013** ^54-56^  NCT01217086 and NCT01571219 | CT-P13 (304) | RCT of EQ (54) | CT-P13/CT-P13 (158) | Transitional design 2 (48) | -- | -- | 102 |
|  | IFX (302) |  | IFX/CT_P13 (144) |  |  |  |  |
| **Kay, 2014** ^57,58^  NCT02683564 | BOW015 (127) | RCT of EQ (16) | BOW015/BOW015 (104) | Transitional design 2 (38) | -- | -- | 54 |
|  | IFX (62) |  | IFX/BOW015 (53) |  |  |  |  |
| **Choe, 2015** ^59-61^  NCT01936181 | SB2 (291) | RCT of EQ (54) | SB2/SB2 (201) | Transitional design 1 (24) | -- | -- | 78 |
|  | IFX (293) |  | IFX/IFX (101) |  |  |  |  |
|  |  |  | INF/SB2 (94) |  |  |  |  |
| **Matsuno, 2018**  ^62^  NCT01927263 | NI071  (126) | RCT of EQ (30) | NI071/NI071 (108) | Transitional design 2 (24) | -- | -- | 54 |
|  | IFX  (116) |  | IFX/NI071 (102) |  |  |  |  |
| **Genovese, 2020** ^63^  NCT02937701 | ABP710 (279) | RCT of EQ (22) | ABP710/ABP710 (244) | Transitional design 1 (28) | -- | -- | 50 |
|  | IFX (279) |  | IFN/IFN (121) |  |  |  |  |
|  |  |  | IFN/ABP710 (108) |  |  |  |  |

Follow-up time in weeks. RCT: randomized controlled trials; EQ: trials that assessed equivalence efficacy between a biosimilar and a biologic drug. NI: trials that assessed non-inferiority efficacy between a biosimilar and biologic drug. ADA: reference adalimumab; ETN: reference etanercept; IFX: reference infliximab.

Single-switch design 1: Trials in which there is a single switch from each treatment to the other. Firstly, patients were randomly allocated to either a biosimilar or a biologic drug (first period). Then, in the second period, treatments were randomly switched in both directions.

Single switch 2: In the study of Yamanaka et al. ^53^, patients were randomly allocated to either a biosimilar or a biologic drug (stage A). Then, in parallel, a group of patients continue the treatments to evaluate long-term safety and immunogenicity (Stage B) and selected patients were crossed over in both directions (switch group/Stage C). However, it was not clear if Stage C was randomized.

Transition design 1 (two non-switching groups as a control): Trials in which there is a single switch from one treatment (biologic drug) to another (biosimilar drug), but not the contrary. Firstly, patients were randomly allocated to either a biosimilar or a biologic drug (first period). Then, in the second period, the trial became a three-arm trial in which patients in the biologic drug group were re-randomized either to continue in the biologic group or to switch to the biosimilar drug treatment. Patients initially allocated to the biosimilar group continued to receive a biosimilar throughout the study period.

Transition design 2 (randomized trials with an open-label extension; single non-switching group as a control): Trials in which there is a single switch from a biologic drug to a biosimilar drug, but not the contrary. Firstly, patients were randomly allocated to either a biosimilar or a biologic drug (first period). Then, in the open-label extended phase (second period), all patients (intervention and control groups) received the biosimilar drug.

Transitional design 3: After the second period of either a single switch or transitional design 1, there was an open-label extended phase (third period), in which all patients (intervention and control groups) received the biosimilar drug.

# Supplementary Results file 3. Risk of bias of included studies

# Supplementary Table S6. Full risk of bias assessment of specific domains of switching studies: domains 1, 2, and 3.

| **Author, year** | **Domain 1 – Randomized** **and blinded design with appropriate control arm** | | | | **Domain 2 – At least a 1-way switch from reference to biosimilar** | | **Domain 3 – The assessment of immunogenicity** | | |
| --- | --- | --- | --- | --- | --- | --- | --- | --- | --- |
|  | Randomization step before the switch | Switching period blinded | Population was selected for positive responses | **Overall judgment domain 1** | At least 1-way switch from reference to biosimilar | **Overall judgment domain 2** | Adequately measured in both arms | Sufficient time for follow-up | **Overall judgment domain 3** |
| Alten, 2017 ^24-30^ | Yes | No | No | High risk | Yes | Low risk | Yes | Yes | Low risk |
| Cohen, 2017 ^31,32^ | No | No | No | High risk | No | High risk | Yes | Yes | Low risk |
| Fleischmann, 2018 ^33-35^ | Yes | Unclear | No | Unclear risk | Yes | Low risk | Yes | Yes | Low risk |
| Cohen, 2018 ^36,37^ | Yes | Unclear | Yes | High risk | Yes | Low risk | Yes | Yes | Low risk |
| Weinblatt, 2018 ^38,39^ | Yes | Yes | Unclear | Low risk | Yes | Low risk | Yes | No | High risk |
| Wiland, 2019 ^40,41^ | No | No | No | Low risk | No | High risk | Yes | No | High risk |
| Kay, 2021 ^42,43^ | Yes | Yes | No | Low risk | Yes | Low risk | Yes | No | High risk |
| Emery, 2015 ^44-46^ | No | No | Unclear | High risk | No | High risk | Yes | Yes | Low risk |
| Odell, 2016 ^47,48^ | No | No | Yes | High risk | No | High risk | Unclear | Unclear | Unclear risk |
| Matsuno, 2017 ^49,50^ | No | No | No | High risk | Yes | Low risk | Yes | No | High risk |
| Matucci-Cerinic, 2018 ^51,52^ | No | No | No | High risk | No | High risk | Yes | Yes | Low risk |
| Yamanaka, 2020 ^53^ | Unclear | Yes | Yes | High risk | Yes | Low risk | Yes | No | High risk |
| Yoo, 2013 ^54-56^ | No | No | Unclear | High risk | No | High risk | Yes | Yes | Low risk |
| Kay, 2014 ^57,58^ | No | No | Yes | High risk | No | High risk | Yes | No | High risk |
| Choe, 2015 ^59-61^ | Yes | Yes | No | Low risk | Yes | Low risk o | Yes | No | High risk |
| Matsuno, 2018 ^62^ | Yes | No | No | High risk | No | High risk | Yes | No | High risk |
| Genovese, 2020 ^63^ | Yes | Unclear | No | Unclear risk | Yes | Low risk | Yes | No | High risk |

# Supplementary Table S7. Full risk of bias assessment of specific domains of switching studies: domains 4, 5, and 6.

| **Author, year** | **Domain 4 – Adequate washout period before switching** | | | **Domain 5 – Enough power to assess efficacy and safety (equivalence studies)** | | | | **Domain 6 – An enough follow-up period** | |
| --- | --- | --- | --- | --- | --- | --- | --- | --- | --- |
|  | **Washout period before the switch** | **Washout was done for enough time** | **Overall judgment domain 4** | **Small number of patients in the switch groups** | **High rate of differential loss of participants before switching** | **The study powered to assess efficacy in individual diseases** | **Overall judgment domain 5** | **Follow-up period after the switch was sufficient** | **Overall judgment domain 6** |
| Alten, 2017 ^24-30^ | Yes | Yes | Low risk | No | No | Yes | Low risk | Yes | Low risk |
| Cohen, 2017 ^31,32^ | Yes | Yes | Low risk | No | No | Yes | Low risk | Yes | Low risk |
| Fleischmann, 2018 ^33-35^ | No | No | High risk | No | No | Yes | Low risk | Yes | Low risk |
| Cohen, 2018 ^36,37^ | No | No | High risk | No | No | Yes | Low risk | Yes | Low risk |
| Weinblatt, 2018 ^38,39^ | No | No | High risk | No | No | Yes | Low risk | Yes | Low risk |
| Wiland, 2019 ^40,41^ | No | No | High risk | No | No | Yes | Low risk | No | High risk |
| Kay, 2021 ^42,43^ | Yes | Yes | Low risk | No | No | Yes | Low risk | No | High risk |
| Emery, 2015 ^44-46^ | No | No | High risk | No | Yes | Yes | High risk | Yes | Low risk |
| Odell, 2016 ^47,48^ | No | Unclear | High risk | No | No | Unclear | Low risk | No | High risk |
| Matsuno, 2017 ^49,50^ | No | No | High risk | No | Yes | Yes | High risk | Yes | Low risk |
| Matucci-Cerinic, 2018 ^51,52^ | No | No | High risk | No | No | Yes | Low risk | No | High risk |
| Yamanaka, 2020 ^53^ | No | No | High risk | Yes | Yes | Yes | High risk | No | High risk |
| Yoo, 2013 ^54-56^ | Unclear | Unclear | Unclear risk | No | Yes | Yes | High risk | Yes | Low risk |
| Kay, 2014 ^57,58^ | No | No | High risk | No | Unclear | Unclear | Unclear risk | Yes | Low risk |
| Choe, 2015 ^59-61^ | Yes | Yes | Low risk | No | Yes | Yes | High risk | Yes | Low risk |
| Matsuno, 2018 ^62^ | No | No | High risk | No | No | No | High risk | No | High risk |
| Genovese, 2020 ^63^ | No | No | High risk | No | No | Yes | Low risk | Yes | Low risk |

# Supplementary Results file 4. Network map for each outcome analyzed.

# Supplementary Figure S2. Evidence network maps for secondary outcomes of efficacy: A) ACR50, B) ACR70, C) SDAI, and D) CDAI.

The graphs in Figures (A) and (B) comprise four nodes representing two non-switching arms (Ref-Ref, Bios-Bios) and two switching arms (Ref-Bios and Bios-Ref), and six edges (arrows). Figures (C) and (D) comprise three nodes representing two non-switching arms (Ref-Ref, Bios-Bios) and one switching (Ref-Bios), and three edges (arrows). The size of nodes is proportional to the number of patients analyzed for interventions. The thickness of lines is proportional to the number of studies contributing to the direct comparison. Ref -Ref: patients taking reference biologics drugs continued the treatment (non-switching group); Bios-Bios: patients taking biosimilars continued the treatment (non-switching group); Ref-Bios: patients taking reference biologics drugs switched to biosimilars (switching group); Bios-Ref: patients taking biosimilars switched to reference biologics drugs (switching group); ACR50: the American College of Rheumatology 50% response criteria; ACR70: the American College of Rheumatology 70% response criteria; SDAI: simplified disease activity score; CDAI: clinical disease activity score).

# Supplementary Figure S3. Evidence network maps for secondary outcomes of efficacy: A) DAS28-ESR, B) DAS28-CRP, and C) mTRSS.

The graphs in Figures (A) and (C) comprise three nodes representing two non-switching arms (Ref-Ref, Bios-Bios) and one switching (Ref-Bios), and three edges (arrows). Figure (B) comprises four nodes representing two non-switching arms (Ref-Ref, Bios-Bios) and two switching arms (Ref-Bios and Bios-Ref), and six edges (arrows). The size of nodes is proportional to the number of patients analyzed for interventions. The thickness of lines is proportional to the number of studies contributing to the direct comparison. Ref -Ref: patients taking reference biologics drugs continued the treatment (non-switching group); Bios-Bios: patients taking biosimilars continued the treatment (non-switching group); Ref-Bios: patients taking reference biologics drugs switched to biosimilars (switching group); Bios-Ref: patients taking biosimilars switched to reference biologics drugs (switching group); DAS28-ESR: disease activity score in 28 joints based on the erythrocyte sedimentation rate; DAS28-CRP: disease activity score in 28 joints with four components based on C-reactive protein; mTRSS: Sharp-Van Der Heidje Modified Score Method.

# Supplementary Figure S4. Evidence network maps for secondary outcomes of safety: A) overall TEAEs, B) Serious TEAEs, C) Hypersensitivity and IRRs.

The graphs in Figures (A) and (B) comprise four nodes representing two non-switching arms (Ref-Ref, Bios-Bios) and two switching arms (Ref-Bios and Bios-Ref), and six edges (arrows). Figures (C) and (D) comprise three nodes representing two non-switching arms (Ref-Ref, Bios-Bios) and one switching (Ref-Bios), and three edges (arrows). The size of nodes is proportional to the number of patients analyzed for interventions. The thickness of lines is proportional to the number of studies contributing to the direct comparison. Ref -Ref: patients taking reference biologics drugs continued the treatment (non-switching group); Bios-Bios: patients taking biosimilars continued the treatment (non-switching group); Ref-Bios: patients taking reference biologics drugs switched to biosimilars (switching group); Bios-Ref: patients taking biosimilars switched to reference biologics drugs (switching group); TEAEs: treatment-emergent adverse events; IRRs: infusion-related reactions.

# Supplementary Figure S5. Evidence network maps for secondary outcomes of safety: A) Malignancies, B) Serious infections, and C) Overall Discontinuation rates.

The graphs in Figures (A) and (B) comprise three nodes representing two non-switching arms (Ref-Ref, Bios-Bios) and one switching (Ref-Bios), and three edges (arrows). Figure (C) comprises four nodes representing two non-switching arms (Ref-Ref, Bios-Bios) and two switching arms (Ref-Bios and Bios-Ref), and six edges (arrows)The size of nodes is proportional to the number of patients analyzed for interventions. The thickness of lines is proportional to the number of studies contributing to the direct comparison. Ref -Ref: patients taking reference biologics drugs continued the treatment (non-switching group); Bios-Bios: patients taking biosimilars continued the treatment (non-switching group); Ref-Bios: patients taking reference biologics drugs switched to biosimilars (switching group); Bios-Ref: patients taking biosimilars switched to reference biologics drugs (switching group).

**
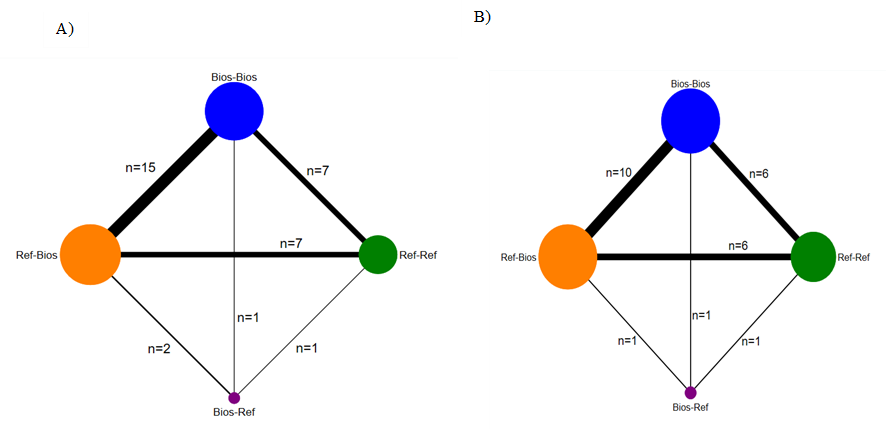
**

# Supplementary Figure S6. Evidence network maps for secondary outcomes of immunogenicity: A) Positive anti-drug antibodies, and B) Positive neutralizing antibodies.

The graphs in Figures (A) and (B) comprise four nodes representing two non-switching arms (Ref-Ref, Bios-Bios) and two switching arms (Ref-Bios and Bios-Ref), and six edges (arrows). The size of nodes is proportional to the number of patients analyzed for interventions. The thickness of lines is proportional to the number of studies contributing to the direct comparison. Ref -Ref: patients taking reference biologics drugs continued the treatment (non-switching group); Bios-Bios: patients taking biosimilars continued the treatment (non-switching group); Ref-Bios: patients taking reference biologics drugs switched to biosimilars (switching group); Bios-Ref: patients taking biosimilars switched to reference biologics drugs (switching group).

# Supplementary Results file 5. Efficacy outcomes: evidence synthesis

# Supplementary Table S8. Efficacy outcomes: Bayesian random-effects network meta-analyses analyzing the impact of switching or non-switching treatments of biosimilars and reference biologics considering all TNFi of interest together and by type of molecule.

| **Outcome** | **Group of interest** | **Control group** | **Trials (n)** | **Patients (n)** | **RR or SMD*** | **95% CrIs** | **95% PI** | **τ^2^** |
| --- | --- | --- | --- | --- | --- | --- | --- | --- |
| **Primary** |  |  |  |  |  |  |  |  |
| **ACR20** | **All TNFi** |  | 15 | 6007 |  |  |  | 0.006 |
|  | Bios-Bios | Ref-Ref | 7 | 2753 | 0.984 | 0.938, 1.025 | 0.911, 1.046 |  |
|  | Ref-Bios | Ref-Ref | 7 | 1926 | 0.982 | 0.930, 1.026 | 0.904, 1.044 |  |
|  | Bios-Ref | Ref-Ref | 1 | 208 | 0.973 | 0.821, 1.092 | 0.809, 1.097 |  |
|  | Ref-Bios | Bios-Bios | 15 | 4934 | 0.997 | 0.963, 1.032 | 0.928, 1.056 |  |
|  | Bios-Ref | Bios-Bios | 1 | 297 | 0.989 | 0.839, 1.113 | 0.826, 1.118 |  |
|  | Bios-Ref | Ref-Bios | 1 | 195 | 0.992 | 0.841, 1.117 | 0.827, 1.123 |  |
|  | **ADA** |  | 7 | 3502 |  |  |  | 0.014 |
|  | Bios-Bios | Ref-Ref | 5 | 2086 | 0.989 | 0.926, 1.041 | 0.877, 1.072 |  |
|  | Ref-Bios | Ref-Ref | 5 | 1399 | 0.989 | 0.921, 1.046 | 0.876, 1.075 |  |
|  | Bios-Ref | Ref-Ref | 1 | 208 | 0.978 | 0.811, 1.098 | 0.785, 1.110 |  |
|  | Ref-Bios | Bios-Bios | 7 | 2651 | 1.001 | 0.949, 1.054 | 0.897, 1.088 |  |
|  | Bios-Ref | Bios-Bios | 1 | 369 | 0.989 | 0.828, 1.117 | 0.800, 1.129 |  |
|  | Bios-Ref | Ref-Bios | 1 | 195 | 0.989 | 0.826, 1.119 | 0.798, 1.131 |  |
|  | **ETN** |  | 4 | 1115 |  |  |  | 0.095 |
|  | Ref-Bios | Bios-Bios | 4 | 1115 | 0.974 | 0.770, 1.109 | 0.502, 1.187 |  |
|  | **IFX** |  | 4 | 1390 |  |  |  | 0.056 |
|  | Bios-Bios | Ref-Ref | 2 | 1156 | 0.970 | 0.798, 1.094 | 0.656, 1.148 |  |
|  | Ref-Bios | Ref-Ref | 2 | 924 | 0.973 | 0.786, 1.092 | 0.647, 1.146 |  |
|  | Ref-Bios | Bios-Bios | 4 | 1168 | 1.003 | 0.877, 1.125 | 0.707, 1.198 |  |
| **HAQ-DI** | **All TNFi** |  | 5 | 1732 |  |  |  | 0.007 |
|  | Bios-Bios | Ref-Ref | 1 | 390 | -0.011 | -0.350, 0.304 | -- |  |
|  | Ref-Bios | Ref-Ref | 1 | 252 | 0.076 | -0.422, 0.243 | -- |  |
|  | Ref-Bios | Bios-Bios | 5 | 1609 | -0.065 | -0.231, 0.098 | -0.416, 0.281 |  |
|  | **ADA** |  | 2 | 753 |  |  |  | 0.348 |
|  | Bios-Bios | Ref-Ref | 1 | 329 | 0.024 | -3.691, 3.721 | -- |  |
|  | Ref-Bios | Ref-Ref | 1 | 252 | -0.136 | -3.827, 3.552 | -- |  |
|  | Ref-Bios | Bios-Bios | 2 | 630 | -0.160 | -2.970, 2.638 | -4.965, 4.666 |  |
|  | **ETN** |  | 3 | 979 |  |  |  | 0.014 |
|  | Ref-Bios | Bios-Bios | 3 | 979 | -0.006 | -0.581, 0.565 | -1.142, 1.112 |  |
| **Secondary** |  |  |  |  |  |  |  |  |
| Efficacy |  |  |  |  |  |  |  |  |
| **ACR50** | **All TNFi** |  | 14 | 5574 |  |  |  | 0.006 |
|  | Bios-Bios | Ref-Ref | 7 | 2753 | 0.982 | 0.903, 1.061 | 0.850, 1.114 |  |
|  | Ref-Bios | Ref-Ref | 7 | 1926 | 0.992 | 0.906, 1.076 | 0.857, 1.125 |  |
|  | Bios-Ref | Ref-Ref | 1 | 208 | 0.939 | 0.721, 1.151 | 0.698, 1.172 |  |
|  | Ref-Bios | Bios-Bios | 14 | 4500 | 1.009 | 0.946, 1.076 | 0.883, 1.134 |  |
|  | Bios-Ref | Bios-Bios | 1 | 297 | 0.956 | 0.739, 1.175 | 0.715, 1.197 |  |
|  | Bios-Ref | Ref-Bios | 1 | 195 | 0.948 | 0.730, 1.170 | 0.709, 1.191 |  |
|  | **ADA** |  | 6 | 3069 |  |  |  | 0.022 |
|  | Bios-Bios | Ref-Ref | 5 | 2086 | 1.018 | 0.804, 1.292 | 0.763, 1.335 |  |
|  | Ref-Bios | Ref-Ref | 5 | 1399 | 1.044 | 0.808, 1.346 | 0.769, 1.351 |  |
|  | Bios-Ref | Ref-Ref | 1 | 208 | 0.919 | 0.544, 1.550 | 0.448, 1,593 |  |
|  | Ref-Bios | Bios-Bios | 6 | 2217 | 1.026 | 0.544, 1.550 | 0.756, 1.679 |  |
|  | Bios-Ref | Bios-Bios | 1 | 369 | 0.904 | 0.536, 1.515 | 0.427, 1.646 |  |
|  | Bios-Ref | Ref-Bios | 1 | 195 | 0.881 | 0.518, 1.490 | 0.489, 1.647 |  |
|  | **ETN** |  | 4 | 1115 |  |  |  | 0.027 |
|  | Ref-Bios | Bios-Bios | 4 | 1115 | 1.015 | 0.679, 1.498 | 0.582, 1.510 |  |
|  | **IFX** |  | 4 | 1390 |  |  |  | 0.032 |
|  | Bios-Bios | Ref-Ref | 2 | 1156 | 0.831 | 0.508, 1.360 | 0.475, 1.543 |  |
|  | Ref-Bios | Ref-Ref | 2 | 924 | 0.831 | 0.493, 1.367 | 0.375, 1.549 |  |
|  | Ref-Bios | Bios-Bios | 4 | 1168 | 1.000 | 0.677, 1.440 | 0.454, 1.596 |  |
| **ACR70** | **All TNFi** |  | 14 | 5573 |  |  |  | 0.014 |
|  | Bios-Bios | Ref-Ref | 7 | 2753 | 0.973 | 0.853, 1.105 | 0.751, 1.232 |  |
|  | Ref-Bios | Ref-Ref | 7 | 1926 | 0.980 | 0.848, 1.120 | 0.750, 1.238 |  |
|  | Bios-Ref | Ref-Ref | 1 | 208 | 0.868 | 0.576, 1.224 | 0.542, 1.283 |  |
|  | Ref-Bios | Bios-Bios | 14 | 4449 | 1.007 | 0.908, 1.110 | 0.781, 1.253 |  |
|  | Bios-Ref | Bios-Bios | 1 | 297 | 0.892 | 0.597, 1.255 | 0.560, 1.317 |  |
|  | Bios-Ref | Ref-Bios | 1 | 195 | 0.885 | 0.591, 1.255 | 0.555, 1.316 |  |
|  | **ADA** |  | 6 | 3068 |  |  |  | 0.032 |
|  | Bios-Bios | Ref-Ref | 5 | 2086 | 0.988 | 0.768, 1.288 | 0.714, 1.382 |  |
|  | Ref-Bios | Ref-Ref | 5 | 1399 | 0.926 | 0.698, 1.222 | 0.612, 1.323 |  |
|  | Bios-Ref | Ref-Ref | 1 | 208 | 0.823 | 0.463, 1.466 | 0.379, 1.529 |  |
|  | Ref-Bios | Bios-Bios | 6 | 2217 | 0.937 | 0.725, 1.193 | 0.648, 1.229 |  |
|  | Bios-Ref | Bios-Bios | 1 | 369 | 0.832 | 0.468, 1.470 | 0.387, 1.530 |  |
|  | Bios-Ref | Ref-Bios | 1 | 195 | 0.889 | 0.497, 1.600 | 0.352, 1.695 |  |
|  | **ETN** |  | 4 | 1115 |  |  |  | 0.037 |
|  | Ref-Bios | Bios-Bios | 4 | 1115 | 1.024 | 0.645, 1.535 | 0.721, 1.683 |  |
|  | **IFX** |  | 4 | 1390 |  |  |  | 0.094 |
|  | Bios-Bios | Ref-Ref | 2 | 1156 | 0.933 | 0.470, 1.749 | 0.360, 1.845 |  |
|  | Ref-Bios | Bios-Bios | 4 | 1168 | 1.034 | 0.507, 1.916 | 0.430, 1.963 |  |
|  | Ref-Bios | Ref-Ref | 2 | 924 | 1.109 | 0.659, 1.800 | 0.760, 1.895 |  |
| **SDAI** | **All TNFi** |  | 4 | 1765 |  |  |  | 0.003 |
|  | Bios-Bios | Ref-Ref | 3 | 1156 | -0.002 | -0.165, 0.162 | -0.267, 0.264 |  |
|  | Ref-Bios | Ref-Ref | 3 | 754 | -0.004 | -0.185, 0.175 | -0.265, 0.289 |  |
|  | Ref-Bios | Bios-Bios | 4 | 1382 | -0.002 | -0.155, 0.150 | -0.264, 0.255 |  |
|  | **ADA** |  | 2 | 1131 |  |  |  | 0.025 |
|  | Bios-Bios | Ref-Ref | 2 | 854 | -0.024 | -0.862, 0.810 | -1.476, 1.417 |  |
|  | Ref-Bios | Ref-Ref | 2 | 559 | -0.007 | -0.840, 0.836 | -1.437, 1.435 |  |
|  | Ref-Bios | Bios-Bios | 2 | 849 | 0.017 | -0.811, 0.853 | -1.409, 1.462 |  |
|  | **ETN** |  | 1 | 238 |  |  |  | -- |
|  | Ref-Bios | Bios-Bios | 1 | 238 | -0.084 | -6.414, 6.217 | -- |  |
|  | **IFX** |  | 1 | 396 |  |  |  | -- |
|  | Bios-Bios | Ref-Ref | 1 | 302 | 0.023 | -6.260, 6.307 | -- |  |
|  | Ref-Bios | Ref-Ref | 1 | 195 | 0.058 | -6.214, 6.323 | -- |  |
|  | Ref-Bios | Bios-Bios | 1 | 295 | 0.030 | -6.248, 6.301 | -- |  |
| **CDAI** | **All TNFi** |  | 4 | 1765 |  |  |  | 0.003 |
|  | Bios-Bios | Ref-Ref | 3 | 1156 | -0.003 | -0.167, 0.162 | -0.270, 0.268 |  |
|  | Ref-Bios | Ref-Ref | 3 | 754 | 0.012 | -0.169, 0.193 | -0.264, 0.290 |  |
|  | Ref-Bios | Bios-Bios | 4 | 1382 | 0.016 | -0.140, 0.169 | -0.249, 0.276 |  |
|  | **ADA** |  | 2 | 1131 |  |  |  | 0.023 |
|  | Bios-Bios | Ref-Ref | 2 | 854 | -0.033 | -0.856, 0.780 | -1.450, 1.360 |  |
|  | Ref-Bios | Ref-Ref | 2 | 559 | 0.010 | -0.814, 0.814 | -1.402, 1.401 |  |
|  | Ref-Bios | Bios-Bios | 2 | 849 | 0.043 | -0.768, 0.855 | -1.360, 1.441 |  |
|  | **ETN** |  | 1 | 138 |  |  |  | -- |
|  | Ref-Bios | Bios-Bios | 1 | 238 | -0.108 | -6.390, 6.169 | -- |  |
|  | **IFX** |  | 1 | 396 |  |  |  | -- |
|  | Bios-Bios | Ref-Ref | 1 | 302 | 0.033 | -6.287, 6.334 | -- |  |
|  | Ref-Bios | Ref-Ref | 1 | 195 | 0.070 | -6.239, 6.379 | -- |  |
|  | Ref-Bios | Bios-Bios | 1 | 295 | 0.035 | -6.239, 6.333 | -- |  |
| **DAS28-ESR** | **All TNFi** |  | 8 | 2632 |  |  |  | 0.002 |
|  | Bios-Bios | Ref-Ref | 3 | 1146 | -0.075 | -0.214, 0.069 | -0.270, 0.127 |  |
|  | Ref-Bios | Ref-Ref | 3 | 744 | -0.056 | -0.204, 0.099 | -0.258, 0.153 |  |
|  | Ref-Bios | Bios-Bios | 8 | 2254 | 0.019 | -0.081, 0.119 | -0.156, 0.193 |  |
|  | **ADA** |  | 3 | 1350 |  |  |  | 0.005 |
|  | Bios-Bios | Ref-Ref | 2 | 844 | -0.140 | -0.402, 0.119 | -0.570, 0.286 |  |
|  | Ref-Bios | Ref-Ref | 2 | 549 | -0.116 | -0.387, 0.156 | -0.549, 0.319 |  |
|  | Ref-Bios | Bios-Bios | 3 | 1073 | 0.025 | -0.200, 0.252 | -0.378, 0.435 |  |
|  | **ETN** |  | 2 | 384 |  |  |  | 0.379 |
|  | Ref-Bios | Bios-Bios | 2 | 384 | -0.032 | -2.844, 2.789 | -4.902, 4.866 |  |
|  | **IFX** |  | 3 | 898 |  |  |  | 0.030 |
|  | Bios-Bios | Ref-Ref | 1 | 302 | 0.086 | -1.084, 1.291 | -- |  |
|  | Ref-Bios | Ref-Ref | 1 | 195 | 0.130 | -1.033, 1.340 | -- |  |
|  | Ref-Bios | Bios-Bios | 3 | 797 | 0.044 | -0.697, 0.798 | -1.438, 1.510 |  |
| **DAS28-CRP** | **All TNFi** |  | 10 | 3725 |  |  |  | 0.015 |
|  | Bios-Bios | Ref-Ref | 4 | 1553 | 0.048 | -0.124, 0.209 | -0.297, 0.379 |  |
|  | Ref-Bios | Ref-Ref | 4 | 1064 | 0.055 | -0.118, 0.225 | -0.286, 0.394 |  |
|  | Bios-Ref | Ref-Ref | 1 | 295 | 0.040 | -0.303, 0.368 | -- |  |
|  | Ref-Bios | Bios-Bios | 10 | 3044 | 0.007 | -0.106, 0.128 | -0.304, 0.327 |  |
|  | Bios-Ref | Bios-Bios | 1 | 295 | -0.007 | -0.340, 0.318v | -- |  |
|  | Bios-Ref | Ref-Bios | 1 | 193 | -0.015 | -0.354, 0.311 | -- |  |
|  | **ADA** |  | 5 | 2377 |  |  |  |  |
|  | Bios-Bios | Ref-Ref | 3 | 1240 | 0.074 | -0.234, 0.370 | -0.532, 0.667 | 0.039 |
|  | Ref-Bios | Ref-Ref | 3 | 847 | 0.068 | -0.239, 0.373 | -0.533, 0.670 |  |
|  | Bios-Ref | Ref-Ref | 1 | 295 | 0.045 | -0.472, 0.535 | -- |  |
|  | Ref-Bios | Bios-Bios | 5 | 1806 | -0.006 | -0.249, 0.246 | -0.574, 0.573 |  |
|  | Bios-Ref | Bios-Bios | 1 | 195 | -0.028 | -0.529, 0.456 | -- |  |
|  | Bios-Ref | Ref-Bios | 1 | 193 | -0.022 | -0.535, 0.463 | -- |  |
|  | **ETN** |  | 2 | 426 |  |  |  | 0.371 |
|  | Ref-Bios | Bios-Bios | 2 | 426 | 0.124 | -2.707, 2.936 | -4.731, 4.974 |  |
|  | **IFX** |  | 3 | 922 |  |  |  | 0.047 |
|  | Bios-Bios | Ref-Ref | 1 | 313 | -0.020 | -1.415, 1.322 | -- |  |
|  | Ref-Bios | Ref-Ref | 1 | 217 | -0.047 | -1.427, 1.307 | -- |  |
|  | Ref-Bios | Bios-Bios | 3 | 812 | -0.026 | -0.879, 0.847 | -1.718, 1.701 |  |
| **mTRSS** | **All TNFi** |  | 3 | 1343 |  |  |  | 0.010 |
|  | Bios-Bios | Ref-Ref | 2 | 854 | -0.096 | -0.431, 0.225 | -0.652, 0.450 |  |
|  | Ref-Bios | Ref-Ref | 2 | 559 | -0.111 | -0.425, 0.236 | -0.647, 0.464 |  |
|  | Ref-Bios | Bios-Bios | 3 | 663 | -0.017 | -0.268, 0.286 | -0.516, 0.535 |  |

Summary effects estimates are RR for binary outcomes and SMD for continuous outcomes.

All TNFi: all tumor necrosis factor inhibitors of interest (adalimumab, etanercept, and infliximab) pooled together; ADA: adalimumab; ETN: etanercept; IFX: infliximab; Ref-Ref: patients taking reference biologics drugs continued the treatment (non-switching group); Bios-Bios: patients taking biosimilars continued the treatment (non-switching group); Ref-Bios: patients taking reference biologics drugs switched to biosimilars (switching group); Bios-Ref: patients taking biosimilars switched to reference biologics drugs (switching group).

RR: Relative Risk; SMD: Standardised Mean Differences; CrIs: credible intervals; PI: predictive interval; τ^2^: Tau squared estimates between-study heterogeneity.

ACR20: the American College of Rheumatology 20% response criteria; ACR50: ACR 50% response criteria; ACR70: ACR 70% response criteria; HAQ-DI: the Health Assessment Questionnaire - Disability Index; CDAI: clinical disease activity score; SDAI: simplified disease activity score; DAS28-ESR: disease activity score in 28 joints based on the erythrocyte sedimentation rate; DAS28-CRP: disease activity score in 28 joints with four components based on C-reactive protein; mTRSS: Sharp-Van Der Heidje Modified Score Method.

# Supplementary Results file 6. Safety outcomes: evidence synthesis

# Supplementary Table S9. Safety outcomes: Bayesian random-effects network meta-analyses analyzing the impact of switching or non-switching treatments of biosimilars and reference biologics of all TNFi of interest.

| **Outcome** | **Intervention** | **Control** | **Trials (n)** | **Patients (n)** | **RR** | **95% CrIs** | **95% PI** | **τ^2^** |
| --- | --- | --- | --- | --- | --- | --- | --- | --- |
| **Overall TEAE** | **All TNFi** |  | 15 | 6277 |  |  |  | 0.004 |
|  | Bios-Bios | Ref-Ref | 7 | 2793 | 0.921 | 0.772, 1.104 | 0.716, 1.189 |  |
|  | Ref-Bios | Ref-Ref | 7 | 1874 | 0.996 | 0.826, 1.203 | 0.767, 1.293 |  |
|  | Bios-Ref | Ref-Ref | 2 | 321 | 1.035 | 0.678, 1.547 | 0.653, 1.597 |  |
|  | Ref-Bios | Bios-Bios | 15 | 5154 | 1.082 | 0.949, 1.233 | 0.862, 1.351 |  |
|  | Bios-Ref | Bios-Bios | 2 | 324 | 1.126 | 0.734, 1.661 | 0.707, 1.718 |  |
|  | Bios-Ref | Ref-Bios | 2 | 234 | 1.039 | 0.675, 1.552 | 0.651, 1.602 |  |
| **Serious TEAE** | **All TNFi** |  | 15 | 6277 |  |  |  | 0.173 |
|  | Bios-Bios | Ref-Ref | 7 | 2793 | 0.882 | 0.461, 1.542 | 0.238, 2.530 |  |
|  | Ref-Bios | Ref-Ref | 7 | 1874 | 1.032 | 0.537, 1.852 | 0.285, 2.964 |  |
|  | Bios-Ref | Ref-Ref | 2 | 321 | 1.796 | 0.524, 4.495 | 0.354, 5.601 |  |
|  | Ref-Bios | Bios-Bios | 15 | 5154 | 1.171 | 0.739, 1.901 | 0.375, 3.417 |  |
|  | Bios-Ref | Bios-Bios | 2 | 324 | 2.020 | 0.627, 5.515 | 0.429, 6.990 |  |
|  | Bios-Ref | Ref-Bios | 2 | 234 | 1.729 | 0.526, 4.707 | 0.360, 5.902 |  |
| **Hypersensitivity** | **All TNFi** |  | 2 | 1158 |  |  |  | 0.585 |
|  | Bios-Bios | Ref-Ref | 2 | 873 | 0.914 | 0.183, 4.177 | 0.065, 7.115 |  |
|  | Ref-Bios | Ref-Ref | 2 | 572 | 0.657 | 0.079, 3.089 | 0.030, 5.565 |  |
|  | Ref-Bios | Bios-Bios | 2 | 871 | 0.727 | 0.087, 3.442 | 0.032, 6.320 |  |
| **IRRs** | **All TNFi** |  | 4 | 1389 |  |  |  | 0.333 |
|  | Bios-Bios | Ref-Ref | 2 | 664 | 1.032 | 0.334, 3.216 | 0.141, 5.589 |  |
|  | Ref-Bios | Ref-Ref | 2 | 435 | 0.847 | 0.262, 2.848 | 0.113, 5.064 |  |
|  | Ref-Bios | Bios-Bios | 4 | 1167 | 0.822 | 0.336, 2.038 | 0.125, 4.082 |  |
| **Malignancies** | **All TNFi** |  | 10 | 4448 |  |  |  | 1.147 |
|  | Bios-Bios | Ref-Ref | 5 | 1926 | 0.783 | 0.161, 3.136 | 0.039, 6.538 |  |
|  | Ref-Bios | Ref-Ref | 5 | 1819 | 0.847 | 0.162, 3.447 | 0.042, 6.915 |  |
|  | Ref-Bios | Bios-Bios | 10 | 3483 | 1.078 | 0.337, 3.361 | 0.071, 9.245 |  |
| **Serious Infection** | **All TNFi** |  | 9 | 3290 |  |  |  | 0.210 |
|  | Bios-Bios | Ref-Ref | 3 | 1047 | 1.809 | 0.442, 6.200 | 0.258, 7.462 |  |
|  | Ref-Bios | Ref-Ref | 3 | 689 | 2.385 | 0.607, 7.237 | 0.373, 8.434 |  |
|  | Ref-Bios | Bios-Bios | 9 | 2925 | 1.286 | 0.680, 2.744 | 0.329, 4.278 |  |
| **Discontinuation rates** | **All TNFi** |  | 10 | 6405 |  |  |  | 0.020 |
|  | Bios-Bios | Ref-Ref | 7 | 2799 | 1.176 | 0.869, 1.574 | 0.715, 1.875 |  |
|  | Ref-Bios | Ref-Ref | 7 | 2029 | 1.094 | 0.787, 1.500 | 0.653, 1.779 |  |
|  | Bios-Ref | Ref-Ref | 1 | 321 | 0.707 | 0.307, 1.463 | 0.280, 1.598 |  |
|  | Ref-Bios | Bios-Bios | 15 | 5279 | 0.931 | 0.748, 1.152 | 0.590, 1.437 |  |
|  | Bios-Ref | Bios-Bios | 1 | 321 | 0.603 | 0.264, 1.234 | 0.242, 1.353 |  |
|  | Bios-Ref | Ref-Bios | 2 | 341 | 0.648 | 0.284, 1.338 | 0.259, 1.470 |  |

All TNFi: all tumor necrosis factor inhibitors of interest (adalimumab, etanercept, and infliximab) pooled together; Ref-Ref: patients taking reference biologics drugs continued the treatment (non-switching group); Bios-Bios: patients taking biosimilars continued the treatment (non-switching group); Ref-Bios: patients taking reference biologics drugs switched to biosimilars (switching group); Bios-Ref: patients taking biosimilars switched to reference biologics drugs (switching group).

RR: Relative Risk; CrIs: credible intervals; PI: predictive interval; τ^2^: Tau squared estimates between-study heterogeneity; TEAEs: treatment-emergent adverse events; IRRs: infusion-related reactions.

# Supplementary Results file 7. Immunogenicity outcomes: evidence synthesis

# Supplementary Table S10. Immunogenicity outcomes: Bayesian random-effects network meta-analyses analyzing the impact of switching or non-switching treatments of biosimilars and reference biologics of all TNFi of interest.

| **Outcome** | **Intervention** | **Control** | **Trials (n)** | **Patients (n)** | **RR** | **95% CrIs** | **95% PI** | **τ^2^** |
| --- | --- | --- | --- | --- | --- | --- | --- | --- |
| **ADAs** | **All TNFi** |  | 16 | 6006 |  |  |  | 0.005 |
|  | Bios-Bios | Ref-Ref | 7 | 2799 | 0.893 | 0.744, 1.065 | 0.679,1.162 |  |
|  | Ref-Bios | Ref-Ref | 7 | 2029 | 0.888 | 0.729, 1.078 | 0.669,1.172 |  |
|  | Bios-Ref | Ref-Ref | 1 | 321 | 1.211 | 0.782, 1.835 | 0.750, 1.905 |  |
|  | Ref-Bios | Bios-Bios | 15 | 5279 | 0.993 | 0.855, 1.158 | 0.773, 1.283 |  |
|  | Bios-Ref | Bios-Bios | 1 | 321 | 1.355 | 0.878, 2.045 | 0.842, 2.126 |  |
|  | Bios-Ref | Ref-Bios | 2 | 341 | 1.362 | 0.881, 2.071 | 0.844, 2.147 |  |
| **Nabs** |  |  | 10 | 1544 |  |  |  | 0.069 |
|  | Bios-Bios | Ref-Ref | 6 | 873 | 0.835 | 0.559, 1.263 | 0.375, 1.830 |  |
|  | Ref-Bios | Ref-Ref | 6 | 594 | 0.738 | 0.475, 1.153 | 0.325, 1.651 |  |
|  | Bios-Ref | Ref-Ref | 1 | 118 | 0.774 | 0.351, 1.687 | 0.271, 2.119 |  |
|  | Ref-Bios | Bios-Bios | 10 | 1130 | 0.882 | 0.605, 1.285 | 0.394, 1.886 |  |
|  | Bios-Ref | Bios-Bios | 1 | 118 | 0.927 | 0.420, 1.986 | 0.322, 2.490 |  |
|  | Bios-Ref | Ref-Bios | 1 | 118 | 1.050 | 0.468, 2.301 | 0.362, 2.865 |  |

Ref-Ref: patients taking reference biologics drugs continued the treatment (non-switching group); Bios-Bios: patients taking biosimilars continued the treatment (non-switching group); Ref-Bios: patients taking reference biologics drugs switched to biosimilars (switching group); Bios-Ref: patients taking biosimilars switched to reference biologics drugs (switching group); RR: Relative Risk; CrIs: credible interval; PI: predictive interval; τ^2^: Tau squared estimates between-study heterogeneity; ADAs: Positive anti-drug antibodies; Nabs: Positive neutralizing antibodies.

# Supplementary Results file 8. Inconsistency and consistency models, and GRADE assessment

# Supplementary Table S11. Efficacy outcomes: inconsistency and consistency models, and certainty of the evidence of direct and network estimates for each pairwise comparison.

|  |  | **Inconsistency model** | |  | **Consistency model** | | | |
| --- | --- | --- | --- | --- | --- | --- | --- | --- |
| **Outcome (No of trials and patients)** |  | **Direct evidence** | |  | **Frequentist fixed effects NMA** |  | **Bayesian random-effects NMA** | |
|  | **Comparison** | **RR or SMD (95% CrIs)** | **Certainty of evidence** |  | **RR or SMD (95% CI)** |  | **RR or SMD (95% CrIs)** | **Certainty of evidence** |
| Primary |  |  |  |  |  |  |  |  |
| **ACR20** |  |  |  |  |  |  |  |  |
| (15 trials, 6007 patients) | Bios-Bios vs. Ref-Ref | 0.938 (0.778, 1.134) | Moderate * |  | 0.981 (0.944, 1.020) |  | 0.984 (0.938, 1.025) | Moderate |
|  | Ref-Bios vs Ref-Ref | 0.913 (0.737, 1.129) | Moderate * |  | 0.977 (0.937, 1.018) |  | 0.982 (0.930, 1.026) | Moderate |
|  | Bios-Ref vs. Ref-Ref | 0.874 (0.490, 1.548) | Low * |  | 0.962(0.873, 1.061) |  | 0.973 (0.821, 1.092) | Low ‡ |
|  | Ref-Bios vs. Bios-Bios | 0.972 (0.795, 1.193) | Low *† |  | 0.995 (0.970, 1.021) |  | 0.997 (0.963, 1.032) | Low |
|  | Bios-Ref vs. Bios-Bios | 0.932 (0.519, 1.659) | Moderate * |  | 0.981(0.889, 1.083) |  | 0.989 0.839, 1.113) | Low ‡ |
|  | Bios-Ref vs. Ref-Bios | 0.957 (0.528, 1.720) | Moderate * |  | 0.986 (0.892, 1.089) |  | 0.992 (0.841, 1.117) | Low ‡ |
| **HAQ-DI** |  |  |  |  |  |  |  |  |
| (5 trials, 1732 patients) | Bios-Bios vs. Ref-Ref | 0.044 (-0.366, 0.454) | Moderate * |  | -0.004 (-0.209, 0.201) |  | -0.011 (-0.350, 0.304) | Low ‡ |
|  | Ref-Bios vs Ref-Ref | -0.169 (-0.592, 0.257) | Moderate * |  | -0.067 (-0.280, 0.146) |  | -0.076 (-0.422, 0.243) | Low ‡ |
|  | Ref-Bios vs. Bios-Bios | -0.025 (-0.239, 0.178) | Moderate * |  | -0.063 (-0.162, 0.036) |  | -0.065 (-0.231, 0.098) | Low ‡ |
| Secondary |  |  |  |  |  |  |  |  |
| **ACR50** |  |  |  |  |  |  |  |  |
| (14 trials, 5574 patients) | Bios-Bios vs. Ref-Ref | 0.951 (0.807, 1.121) | Moderate * |  | 0.990 (0.926, 1.060) |  | 0.982 (0.903, 1.061) | Moderate |
|  | Ref-Bios vs Ref-Ref | 1.008 (0.835, 1.212) | Moderate * |  | 1.005 (0.934, 1.080) |  | 0.992 (0.906, 1.076) | Moderate |
|  | Bios-Ref vs. Ref-Ref | 0.863 (0.540, 1.348) | Moderate * |  | 0.935 (0.772, 1.132) |  | 0.939 (0.721, 1.151) | Low ‡ |
|  | Ref-Bios vs. Bios-Bios | 1.060 (0.885, 1.269) | Low *† |  | 1.014 (0.967, 1.063) |  | 1.009 (0.946, 1.076) | Low |
|  | Bios-Ref vs. Bios-Bios | 0.909 (0.566, 1.422) | Moderate * |  | 0.944 (0.779, 1.143) |  | 0.956 (0.739, 1.175) | Low ‡ |
|  | Bios-Ref vs. Ref-Bios | 0.857 (0.530, 1.357) | Moderate * |  | 0.931 (0.767, 1.130) |  | 0.948 (0.730, 1.170) | Low ‡ |
| **ACR70** |  |  |  |  |  |  |  |  |
| (14 trials, 5573 patients) | Bios-Bios vs. Ref-Ref | 0.967 (0.807, 1.164) | Moderate * |  | 0.965 (0.869, 1.072) |  | 0.973 (0.853, 1.105) | Moderate |
|  | Ref-Bios vs Ref-Ref | 0.960 (0.781, 1.178) | Moderate * |  | 0 .986 (0.880, 1.105) |  | 0.980 (0.848, 1.120) | Moderate |
|  | Bios-Ref vs. Ref-Ref | 0.789 (0.453, 1.296) | Moderate * |  | 0.843 (0.608, 1.168) |  | 0.868 (0.576, 1.224) | Low ‡ |
|  | Ref-Bios vs. Bios-Bios | 0.994 (0.795, 1.226) | Low *† |  | 1.022 (0.948, 1.102) |  | 1.007 (0.908, 1.110) | Low |
|  | Bios-Ref vs. Bios-Bios | 0.816 (0.461, 1.349) | Moderate * |  | 0.873 (0.630, 1.211) |  | 0.892 (0.597, 1.255) | Low ‡ |
|  | Bios-Ref vs. Ref-Bios | 0.821 (0.465, 1.376) | Moderate * |  | 0.855 (0.614, 1.190) |  | 0.885 (0.591, 1.255) | Low ‡ |
| **SDAI** |  |  |  |  |  |  |  |  |
| (4 trials, 1765 patients) | Bios-Bios vs. Ref-Ref | -0.008 (-0.191, 0.170) | Moderate * |  | -0.005 (-0.126, 0.116) |  | -0.002 (-0.165, 0.162) | Moderate |
|  | Ref-Bios vs Ref-Ref | 0.011 (-0.190, 0.211) | Moderate * |  | -0.007 (-0.144, 0.131) |  | -0.004 (-0.185, 0.175) | Moderate |
|  | Ref-Bios vs. Bios-Bios | -0.087 (-0.432, 0.258) | Moderate * |  | -0.002 (-0.113, 0.109) |  | -0.002 (-0.155, 0.150) | Moderate |
| **CDAI** |  |  |  |  |  |  |  |  |
| (4 trials, 1765 patients) | Bios-Bios vs. Ref-Ref | -0.010 (-0.191, 0.167) | Moderate * |  | -0.007 (-0.128, 0.114) |  | -0.003 (-0.167, 0.162) | Moderate |
|  | Ref-Bios vs Ref-Ref | 0.030 (-0.169, 0.228) | Moderate * |  | 0.009 (-0.128, 0.147) |  | 0.012 (-0.169, 0.193) | Moderate |
|  | Ref-Bios vs. Bios-Bios | -0.076 (-0.418, 0.268) | Moderate * |  | 0.017 (-0.094, 0.128) |  | 0.016 (-0.140, 0.169) | Moderate |
| **DAS28-ESR** |  |  |  |  |  |  |  |  |
| (8 trials, 2632 patients) | Bios-Bios vs. Ref-Ref | -0.080 (-0.226, 0.070) | Moderate * |  | -0.079 (-0.199, 0.040) |  | -0.075 (-0.214, 0.069) | Moderate |
|  | Ref-Bios vs Ref-Ref | -0.046 (-0.214, 0.126) | Moderate * |  | -0.061 (-0.190, 0.069) |  | -0.056 (-0.204, 0.099) | Moderate |
|  | Ref-Bios vs. Bios-Bios | 0.006 (-0.128, 0.141) | Moderate * |  | 0.018 (-0.067, 0.104) |  | 0.019 (-0.081, 0.119) | Moderate |
| **DAS28-CRP** |  |  |  |  |  |  |  |  |
| (10 trials, 3725 patients) | Bios-Bios vs. Ref-Ref | 0.082 (-0.085, 0.240) | Moderate * |  | 0.060 (-0.039, 0.160) |  | 0.048 (-0.124, 0.209) | Moderate |
|  | Ref-Bios vs Ref-Ref | 0.011 (-0.163, 0.188) | Moderate * |  | 0.062 (-0.046, 0.171) |  | 0.055 (-0.118, 0.225) | Moderate |
|  | Bios-Ref vs. Ref-Ref | 0.084 (-0.245, 0.442) | Moderate * |  | 0.060 (-0.039, 0.160) |  | 0.040 (-0.303, 0.368) | Moderate§ |
|  | Ref-Bios vs. Bios-Bios | 0.065 (-0.077, 0.207) | Moderate * |  | 0.002 (-0.071, 0.075) |  | 0.007 (-0.106, 0.128) | Moderate |
|  | Bios-Ref vs. Bios-Bios | -0.010 (-0.200, 1.960) | Moderate * |  | -0.001 (-0.222, 0.221) |  | -0.007 (-0.340, 0.318) | Moderate§ |
|  | Bios-Ref vs. Ref-Bios | -0.020 (-0.200, 1.190) | Moderate * |  | -0.002 (-0.229, 0.223) |  | -0.015 (-0.354, 0.311) | Moderate§ |
| **mTRSS** |  |  |  |  |  |  |  |  |
| (3 trials, 1343 patients) | Bios-Bios vs. Ref-Ref | -0.084 (-0.789, 0.625) | Moderate * |  | -0.093 (-0.234, 0.048) |  | -0.096 (-0.431, 0.225) | Moderate§ |
|  | Ref-Bios vs Ref-Ref | -0.129 (-0.823, 0.598) | Moderate * |  | -0.099 (-0.258, 0.061) |  | -0.111 (-0.425, 0.236) | Moderate§ |
|  | Ref-Bios vs. Bios-Bios | 0.106 (-0.905, 1.117) | Moderate * |  | -0.005 (-0.132, 0.121) |  | -0.017 (-0.268, 0.286) | Moderate§ |

* Limitation (risk of bias) ‡Imprecision; § Greater precision; ¥ Inconsistency; †Publication bias. Summary effects estimates are RR for binary outcomes and SMD for the continuous outcome.

Ref-Ref: patients taking reference biologics drugs continued the treatment (non-switching group); Bios-Bios: patients taking biosimilars continued the treatment (non-switching group); Ref-Bios: patients taking reference biologics drugs switched to biosimilars (switching group); Bios-Ref: patients taking biosimilars switched to reference biologics drugs (switching group). See **Supplementary Methods file 12** for full judgment criteria.

RR: Relative Risk; SMD: Standardised Mean Differences; CrIs: credible intervals; CI: confidence interval; ACR20: the American College of Rheumatology 20% response criteria ACR50: ACR 50% response criteria; ACR70: ACR 70% response criteria; HAQ-DI: the Health Assessment Questionnaire - Disability Index (HAQ-DI); CDAI: clinical disease activity score; SDAI: simplified disease activity score; DAS28-ESR: disease activity score in 28 joints based on the erythrocyte sedimentation rate; DAS28-CRP: disease activity score in 28 joints with four components based on C-reactive protein; mTRSS: Sharp-Van Der Heidje Modified Score Method.

# Supplementary Table S12. Safety outcomes: inconsistency and consistency models, and certainty of the evidence of direct and network estimates for each pairwise comparison.

|  |  | **Inconsistency model** | |  | **Consistency model** | | | |
| --- | --- | --- | --- | --- | --- | --- | --- | --- |
| **Outcome (No of trials and patients)** |  | **Direct evidence** | |  | **Frequentist fixed effects NMA** |  | **Bayesian random-effects NMA** | |
|  | **Comparison** | **RR (95% CrIs)** | **Certainty of evidence** |  | **RR (95% CI)** |  | **RR (95% CrIs)** | **Certainty of evidence** |
| **TEAEs** |  |  |  |  |  |  |  |  |
| (15 trials, 6277 patients) | Bios-Bios vs. Ref-Ref | 0.899 (0.747, 1.077) | Moderate * |  | 0.944 (0.844, 1.057) |  | 0.921 (0.772, 1.104) | Moderate |
|  | Ref-Bios vs Ref-Ref | 1.043 (0.847, 1.277) | Moderate * |  | 0.986 (0.876, 1.110) |  | 0.996 (0.826, 1.203) | Moderate |
|  | Bios-Ref vs. Ref-Ref | 1.061 (0.683, 1.604) | Moderate * |  | 1.014 (0.831, 1.237) |  | 1.035 (0.678, 1.547) | Low ‡ |
|  | Ref-Bios vs. Bios-Bios | 1.162 (0.952, 1.414) | Moderate * |  | 1.045 (0.974, 1.121) |  | 1.082 (0.949, 1.233) | Moderate |
|  | Bios-Ref vs. Bios-Bios | 1.182 (0.760, 1.797) | Moderate * |  | 1.074 (0.882, 1.308) |  | 1.126 (0.734, 1.661) | Low ‡ |
|  | Bios-Ref vs. Ref-Bios | 1.017 (0.647, 1.572) | Moderate * |  | 1.023 (0.841, 1.255) |  | 1.039 (0.675, 1.552) | Low ‡ |
| **Serious TEAEs** |  |  |  |  |  |  |  |  |
| (15 trials, 6277 patients) | Bios-Bios vs. Ref-Ref | 0.942 (0.492, 1.700) | Low *¥ |  | 0.917 (0.564, 1.489) |  | 0.882 (0.461, 1.542) | Very low‡ |
|  | Ref-Bios vs Ref-Ref | 0.928 (0.428, 1.745) | Low *¥ |  | 1.216 (0.711, 2.079) |  | 1.032 (0.537, 1.852) | Very low‡ |
|  | Bios-Ref vs. Ref-Ref | 1.780 (0.461, 4.881) | Low *¥ |  | 2.038 (0.830, 5.005) |  | 1.796 (0.524, 4.495) | Very low‡ |
|  | Ref-Bios vs. Bios-Bios | 0.987 (0.432, 2.020) | Very low *¥† |  | 1.327 (0.872, 2.018) |  | 1.171 (0.739, 1.901) | Very low‡ |
|  | Bios-Ref vs. Bios-Bios | 1.880 (0.467, 5.993) | Low *¥ |  | 2.223 (0.908, 5.444) |  | 2.020 (0.627, 5.515) | Very low ‡ |
|  | Bios-Ref vs. Ref-Bios | 1.908 (0.477, 6.624) | Low *¥ |  | 1.675 (0.676, 4.149) |  | 1.729 (0.526, 4.707) | Very low ‡ |
| **Hypersensitivity** |  |  |  |  |  |  |  |  |
| (2 trials, 1158 patients) | Bios-Bios vs. Ref-Ref | 0.937 (0.184, 4.356) | Low *¥ |  | 0.803 (0.330, 1.955) |  | 0.914 (0.183, 4.177) | Very low ‡ |
|  | Ref-Bios vs Ref-Ref | 0.618 (0.067, 2.954) | Low *¥ |  | 0.772 (0.261, 2.287) |  | 0.657 (0.079, 3.089) | Very low ‡ |
|  | Ref-Bios vs. Bios-Bios | 0.677 (0.048, 4.751) | Low *¥ |  | 0.961 (0.356, 2.592) |  | 0.727 (0.087, 3.442) | Very low ‡ |
| **IRRs** |  |  |  |  |  |  |  |  |
| (4 trials, 1389 patients) | Bios-Bios vs. Ref-Ref | 1.050 (0.270, 3.820) | Low *¥ |  | 0.916 (0.497, 1.688) |  | 1.032 (0.334, 3.216) | Very low ‡ |
|  | Ref-Bios vs Ref-Ref | 0.797 (0.187, 3.292) | Low *¥ |  | 0.751 (0.368, 1.532) |  | 0.847 (0.262, 2.848) | Very low ‡ |
|  | Ref-Bios vs. Bios-Bios | 0.757 (0.124, 4.759) | Low *¥ |  | 0.819 (0.481, 1.394) |  | 0.822 (0.336, 2.038) | Very low ‡ |
| **Malignancies** |  |  |  |  |  |  |  |  |
| (10 trials, 4448 patients) | Bios-Bios vs. Ref-Ref | 0.800 (0.131, 3.614) | Low *¥ |  | 0.866 (0.301, 2.492) |  | 0.783 (0.161, 3.136) | Very low ‡ |
|  | Ref-Bios vs Ref-Ref | 0.601 (0.068, 3.145) | Low *¥ |  | 1.006 (0.334, 3.025 |  | 0.847 (0.162, 3.447) | Very low ‡ |
|  | Ref-Bios vs. Bios-Bios | 0.761 (0.071, 6.007) | Low *¥ |  | 1.160 (0.504, 2.673) |  | 1.078 (0.337, 3.361) | Very low ‡ |
| **Serious infections** |  |  |  |  |  |  |  |  |
| (9 trials, 3290 patients) | Bios-Bios vs. Ref-Ref | 1.291 (0.224, 5.239) | Low *¥ |  | 1.934 (0.471, 7.933) |  | 1.809 (0.442, 6.200) | Very low ‡ |
|  | Ref-Bios vs Ref-Ref | 3.147 (0.795, 8.351) | Low *¥ |  | 2.416 (0.604, 9.664) |  | 2.385 (0.607, 7.237) | Very low ‡ |
|  | Ref-Bios vs. Bios-Bios | 2.261 (0.684, 12.450) | Low *¥ |  | 1.249 (0.636, 2.451) |  | 1.286 (0.680, 2.744) | Very low ‡ |
| **Discontinuation Rates** |  |  |  |  |  |  |  |  |
| (10 trials, 6405 patients) | Bios-Bios vs. Ref-Ref | 1.163 (0.848, 1.580) | Moderate * |  | 1.170 (0.888, 1.542) |  | 1.176 (0.869, 1.574) | Low ‡ |
|  | Ref-Bios vs Ref-Ref | 1.118 (0.774, 1.583) | Moderate * |  | 1.095 (0.812, 1.477) |  | 1.094 (0.787, 1.500) | Low ‡ |
|  | Bios-Ref vs. Ref-Ref | 0.650 (0.264, 1.414) | Moderate * |  | 0.711 (0.349, 1.447) |  | 0.707 (0.307, 1.463) | Low ‡ |
|  | Ref-Bios vs. Bios-Bios | 0.961 (0.678, 1.363) | Moderate * |  | 1.447 (0.769, 1.139) |  | 0.931 (0.748, 1.152) | Moderate |
|  | Bios-Ref vs. Bios-Bios | 0.560 (0.224, 1.240) | Moderate * |  | 0.608 (0.302, 1.220) |  | 0.603 (0.264, 1.234) | Low ‡ |
|  | Bios-Ref vs. Ref-Bios | 0.582 (0.231, 1.306) | Moderate * |  | 0.649 (0.321, 1.312) |  | 0.648 (0.284, 1.338) | Low ‡ |

* Limitation (risk of bias) ‡Imprecision; § Greater precision; ¥ Inconsistency; †Publication bias.

Ref-Ref: patients taking reference biologics drugs continued the treatment (non-switching group); Bios-Bios: patients taking biosimilars continued the treatment (non-switching group); Ref-Bios: patients taking reference biologics drugs switched to biosimilars (switching group); Bios-Ref: patients taking biosimilars switched to reference biologics drugs (switching group).

RR: Relative Risk; CrIs: credible intervals; CI: confidence interval; TEAEs: treatment-emergent adverse events; IRRs: infusion-related reactions.

# Supplementary Table S13. Immunogenicity outcomes: inconsistency and consistency models, and GRADE assessment of the certainty of evidence from direct evidence and network evidence for each pairwise comparison.

|  |  | **Inconsistency model** | |  | **Consistency model** | | | |
| --- | --- | --- | --- | --- | --- | --- | --- | --- |
| **Outcome (No of trials and patients)** |  | **Direct evidence** | |  | **Frequentist fixed effects NMA** |  | **Bayesian random-effects NMA** | |
|  | **Comparison** | **RR (95% CrIs)** | **Quality of evidence** |  | **RR (95% CI)** |  | **RR (95% CrIs)** | **Quality of evidence** |
| **ADAs** |  |  |  |  |  |  |  |  |
| (16 trials, 6006 patients) | Bios-Bios vs. Ref-Ref | 0.888 (0.739, 1.061) | Moderate * |  | 0.932 (0.848, 1.024) |  | 0.893 (0.744, 1.065) | Moderate |
|  | Ref-Bios vs Ref-Ref | 0.893 (0.725, 1.097) | Moderate * |  | 0.928 (0.832, 1.034) |  | 0.888 (0.729, 1.078) | Low ‡ |
|  | Bios-Ref vs. Ref-Ref | 1.259 (0.808, 1.935) | Moderate * |  | 1.105 (0.909, 1.344) |  | 1.211 (0.782, 1.835) | Low ‡ |
|  | Ref-Bios vs. Bios-Bios | 1.006 (0.824, 1.236) | Moderate * |  | 0.997 (0.909, 1.092) |  | 0.993 (0.855, 1.158) | Moderate |
|  | Bios-Ref vs. Bios-Bios | 1.418 (0.901, 2.196) | Moderate * |  | 1.186 (0.977, 1.441) |  | 1.355 (0.878, 2.045) | Low ‡ |
|  | Bios-Ref vs. Ref-Bios | 1.410 (0.886, 2.199) | Moderate * |  | 1.190 (0.973, 1.456) |  | 1.362 (0.881, 2.071) | Low ‡ |
| **Nabs** |  |  |  |  |  |  |  |  |
| (10 trials, 1544 patients) | Bios-Bios vs. Ref-Ref | 0.848 (0.565, 1.287 | Moderate * |  | 0.942 (0.857, 1.035) |  | 0.835 (0.559, 1.263) | Low ‡ |
|  | Ref-Bios vs Ref-Ref | 0.714 (0.449, 1.129) | Moderate * |  | 0.944 (0.858, 1.039) |  | 0.738 (0.475, 1.153) | Low ‡ |
|  | Bios-Ref vs. Ref-Ref | 0.745 (0.301, 1.677) | Moderate * |  | 0.888 (0.668, 1.182) |  | 0.774 (0.351, 1.687) | Low ‡ |
|  | Ref-Bios vs. Bios-Bios | 0.843 (0.491, 1.410) | Moderate * |  | 1.002 (0.974, 1.031) |  | 0.882 (0.605, 1.285) | Low ‡ |
|  | Bios-Ref vs. Bios-Bios | 0.881 (0.332, 2.077) | Moderate * |  | 0.943 (0.708, 1.255) |  | 0.927 (0.420, 1.986) | Low ‡ |
|  | Bios-Ref vs. Ref-Bios | 1.044 (0.390, 2.554) | Moderate * |  | 0.941 (0.706, 1.252) |  | 1.050 (0.468, 2.301) | Low ‡ |

* Limitation (risk of bias) ‡Imprecision; § Greater precision; ¥ Inconsistency; †Publication bias.

Ref-Ref: patients taking reference biologics drugs continued the treatment (non-switching group); Bios-Bios: patients taking biosimilars continued the treatment (non-switching group); Ref-Bios: patients taking reference biologics drugs switched to biosimilars (switching group); Bios-Ref: patients taking biosimilars switched to reference biologics drugs (switching group).

RR: Relative Risk; CrI: credible intervals; CI: confidence interval; ADAs: Positive anti-drug antibodies; Nabs: Positive neutralizing antibodies

# Supplementary Results file 9. Deviance information criteria

# Supplementary Table S14. Deviance information criteria values of consistency and inconsistency models.

| **Outcome** | **DIC**  **Consistency model** | **DIC**  **Inconsistency model** | **Difference DIC**  **(Consistency – Inconsistency)** |
| --- | --- | --- | --- |
| **Binary (Efficacy)** |  |  |  |
| ACR20 | 241.8 | 243.6 | -1.80 |
| ACR50 | 246.3 | 247.8 | -1.50 |
| ACR70 | 249.4 | 251.1 | -1.70 |
| **Binary (Safety)** |  |  |  |
| Overall TEAE | 252.4 | 254.1 | -1.70 |
| Hypersensitivity | 25.89 | 25.8 | 0.09 |
| Overall Serious TEAE | 167.2 | 167.5 | -0.30 |
| Infusions | 54.74 | 55.91 | -1.17 |
| Injection | 77.47 | 76.92 | 0.55 |
| Malignancies | 80.49 | 79.83 | 0.66 |
| Serious Infections | 78.05 | 77.94 | 0.11 |
| Overall Discontinuation | 219.8 | 220.7 | -0.90 |
| **Binary (Immunogenicity)** | |  |  |
| ADAs | 229 | 229.8 | -0.80 |
| NABs | 135 | 136.6 | -1.60 |
| **Continuous (Efficacy)** |  |  |  |
| HAQ-DI | -16.34 | -16.29 | -0.05 |
| CDAI | -18.5 | -17.1 | -1.40 |
| SDAI | -18.58 | -17.11 | -1.47 |
| DAS28-CRP | -30.76 | -31.04 | 0.28 |
| DAS28-ESR | -30.24 | -28.27 | -1.97 |
| MTRSS | -13.08 | -12.25 | -0.83 |

DIC: deviance information criteria.

# Supplementary Results file 10. Publication bias assessment

**
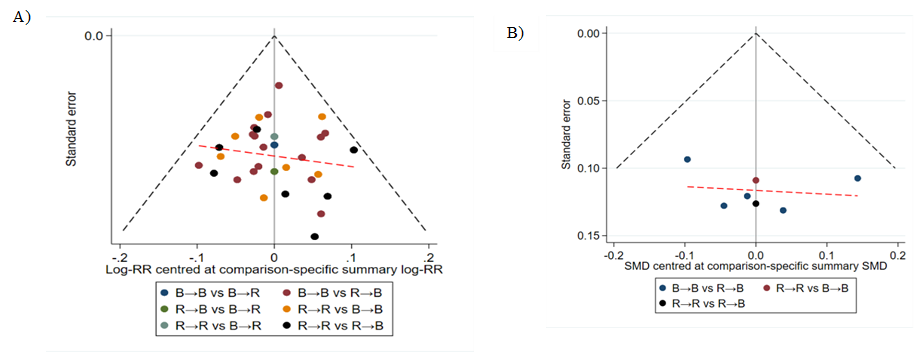
**

# Supplementary Figure S7. Comparison-adjusted funnel plot for primary outcomes: ACR 20.

The horizontal axis shows the difference between the study-specific effect sizes from the corresponding comparison-specific summary effect. The x-axis represents the difference between the ln (RR) for each study-specific effect estimate and the pooled effect estimate for each comparison The gray line represents the null hypothesis that the study-specific effect sizes do not differ from the respective comparison-specific pooled effect estimates. The red diagonal line represents the regression line. Comparisons are color-coded, corresponding to different comparisons between switching and non-switching arms.

B🡪B vs. B🡪R: patients who continued to take biosimilars vs. patients who switched from biosimilars to reference; R🡪B vs. B🡪R: patients who switched from reference to biosimilars vs. patients who switched from biosimilars to reference; R🡪R vs. B🡪R: patients who continued to take reference vs. patients who switched from biosimilars to reference; B🡪B vs. R🡪B: patients who continued to take biosimilars vs. patients who switched from reference to biosimilars; R🡪R vs. B🡪B: patients who continued to take reference vs. patients who continued to take biosimilars; R🡪R vs. R🡪B: patients who continued to take reference vs. patients who switched from reference to biosimilars.

ACR20: the American College of Rheumatology 20% response criteria; RR: relative risk.

# Supplementary Figure S8. Comparison-adjusted funnel plot for secondary outcomes of efficacy: A) ACR50 and B) ACR70.

The horizontal axis shows the difference between the study-specific effect sizes from the corresponding comparison-specific summary effect. The x-axis represents the difference between the ln (RR) for each study-specific effect estimate and the pooled effect estimate for each comparison The grey line represents the null hypothesis that the study-specific effect sizes do not differ from the respective comparison-specific pooled effect estimates. The red diagonal line represents the regression line. Comparisons are color-coded as per the legend at the bottom of the figure, corresponding to different comparisons between switching and non-switching arms.

ACR50: the American College of Rheumatology 50% response criteria; ACR70: the American College of Rheumatology 70% response criteria; RR: relative risk; B🡪B vs. B🡪R: patients who continued to take biosimilars vs. patients who switched from biosimilars to reference; R🡪B vs. B🡪R: patients who switched from reference to biosimilars vs. patients who switched from biosimilars to reference; R🡪R vs. B🡪R: patients who continued to take reference vs. patients who switched from biosimilars to reference; B🡪B vs. R🡪B: patients who continued to take biosimilars vs. patients who switched from reference to biosimilars; R🡪R vs. B🡪B: patients who continued to take reference vs. patients who continued to take biosimilars; R🡪R vs. R🡪B: patients who continued to take reference vs. patients who switched from reference to biosimilars.


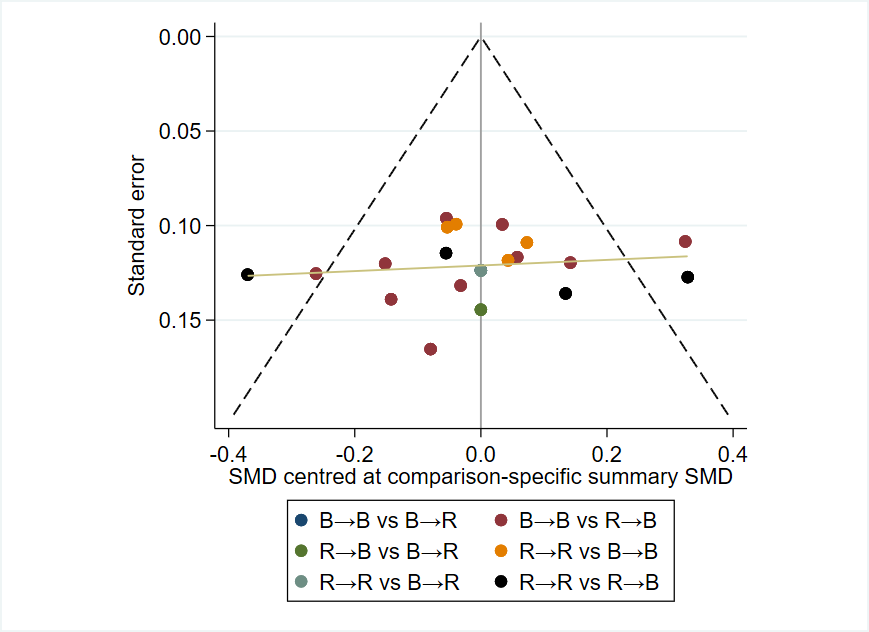


# Supplementary Figure S9. Comparison-adjusted funnel plot for secondary outcomes of efficacy: DAS28-CRP.

The horizontal axis shows the difference between the study-specific effect sizes from the corresponding comparison-specific summary effect. The x-axis represents the difference between the SMD for each study-specific effect estimate and the pooled effect estimate for each comparison The grey line represents the null hypothesis that the study-specific effect sizes do not differ from the respective comparison-specific pooled effect estimates. The red diagonal line represents the regression line. Comparisons are color-coded as per the legend at the bottom of the figure, corresponding to different comparisons between switching and non-switching arms.

DAS28-CRP: disease activity score in 28 joints with four components based on C-reactive protein; SMD: standardized mean differences; B🡪B vs. B🡪R: patients who continued to take biosimilars vs. patients who switched from biosimilars to reference; R🡪B vs. B🡪R: patients who switched from reference to biosimilars vs. patients who switched from biosimilars to reference; R🡪R vs. B🡪R: patients who continued to take reference vs. patients who switched from biosimilars to reference; B🡪B vs. R🡪B: patients who continued to take biosimilars vs. patients who switched from reference to biosimilars; R🡪R vs. B🡪B: patients who continued to take reference vs. patients who continued to take biosimilars; R🡪R vs. R🡪B: patients who continued to take reference vs. patients who switched from reference to biosimilars.


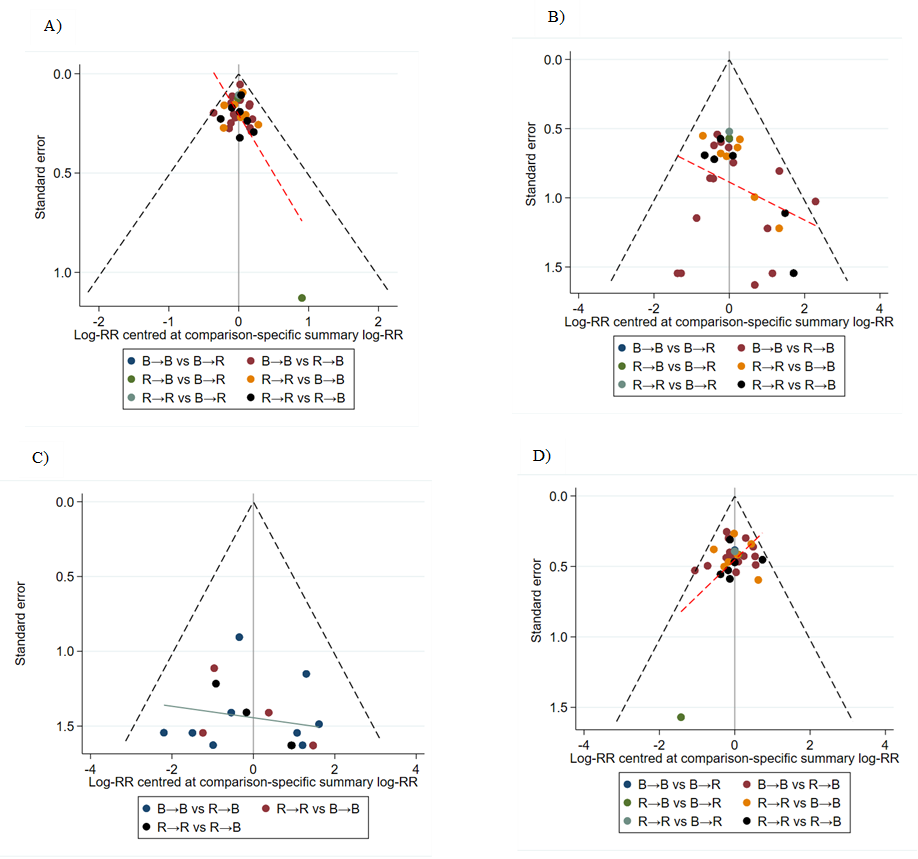


# Supplementary Figure S10. Comparison-adjusted funnel plot for safety outcomes: A) overall TEAEs, B) serious TEAEs, C) Malignancies, and D) overall Discontinuation rates.

The horizontal axis shows the difference between the study-specific effect sizes from the corresponding comparison-specific summary effect. The x-axis represents the difference between the ln (RR) for each study-specific effect estimate and the pooled effect estimate for each comparison The grey line represents the null hypothesis that the study-specific effect sizes do not differ from the respective comparison-specific pooled effect estimates. The red diagonal line represents the regression line. Comparisons are color-coded as per the legend at the bottom of the figure, corresponding to different comparisons between switching and non-switching arms. TEAEs: treatment-emergent adverse events; RR: relative risk; B🡪B vs. B🡪R: patients who continued to take biosimilars vs. patients who switched from biosimilars to reference; R🡪B vs. B🡪R: patients who switched from reference to biosimilars vs. patients who switched from biosimilars to reference; R🡪R vs. B🡪R: patients who continued to take reference vs. patients who switched from biosimilars to reference; B🡪B vs. R🡪B: patients who continued to take biosimilars vs. patients who switched from reference to biosimilars; R🡪R vs. B🡪B: patients who continued to take reference vs. patients who continued to take biosimilars; R🡪R vs. R🡪B: patients who continued to take reference vs. patients who switched from reference to biosimilars.


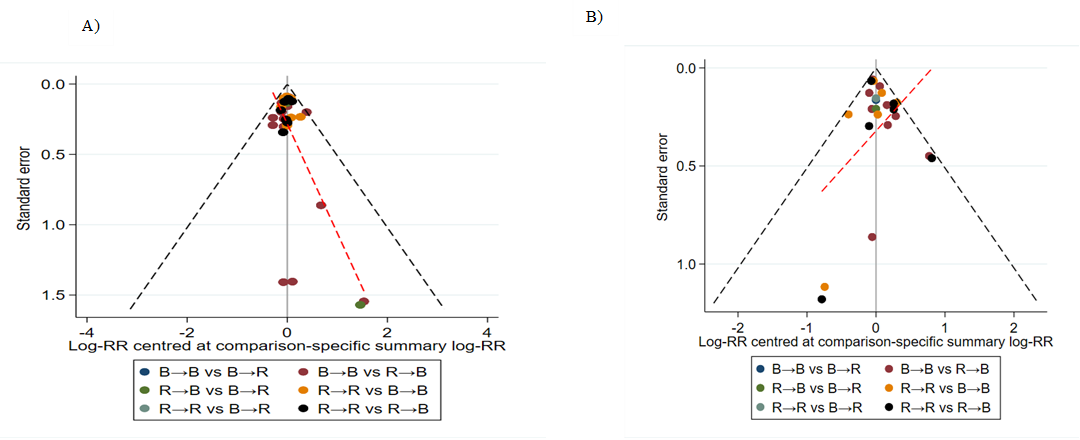


# Supplementary Figure S11. Comparison-adjusted funnel plot for immunogenicity outcomes: A) Positive anti-drug antibodies, and B) Positive neutralizing antibodies.

The horizontal axis shows the difference between the study-specific effect sizes from the corresponding comparison-specific summary effect. The x-axis represents the difference between the ln (RR) for each study-specific effect estimate and the pooled effect estimate for each comparison The gray line represents the null hypothesis that the study-specific effect sizes do not differ from the respective comparison-specific pooled effect estimates. The red diagonal line represents the regression line. Comparisons are color-coded as per the legend at the bottom of the figure, corresponding to different comparisons between switching and non-switching arms. RR: relative risk; B🡪B vs. B🡪R: patients who continued to take biosimilars vs. patients who switched from biosimilars to reference; R🡪B vs. B🡪R: patients who switched from reference to biosimilars vs. patients who switched from biosimilars to reference; R🡪R vs. B🡪R: patients who continued to take reference vs. patients who switched from biosimilars to reference; B🡪B vs. R🡪B: patients who continued to take biosimilars vs. patients who switched from reference to biosimilars; R🡪R vs. B🡪B: patients who continued to take reference vs. patients who continued to take biosimilars; R🡪R vs. R🡪B: patients who continued to take reference vs. patients who switched from reference to biosimilars

# Supplementary References file 1. References

1 Ascef, B. O. *et al.* Equivalence and switching between biosimilars and reference molecules in rheumatoid arthritis: protocol for a systematic review and meta-analysis. *Syst Rev* **10**, 205, doi:10.1186/s13643-021-01754-x (2021).

2 US Food and Drug Administration. (ed U.S. Department of Health and Human Services) (US Food and Drug Administration, Silver Spring, 2019).

3 Barbier, L. *et al.* The Efficacy, Safety, and Immunogenicity of Switching Between Reference Biopharmaceuticals and Biosimilars: A Systematic Review. *Clin Pharmacol Ther* **108**, 734-755, doi:10.1002/cpt.1836 (2020).

4 Efthimiou, O. *et al.* GetReal in network meta-analysis: a review of the methodology. *Res Synth Methods* **7**, 236-263, doi:10.1002/jrsm.1195 (2016).

5 Jansen, J. P. *et al.* Interpreting indirect treatment comparisons and network meta-analysis for health-care decision making: report of the ISPOR Task Force on Indirect Treatment Comparisons Good Research Practices: part 1. *Value Health* **14**, 417-428, doi:10.1016/j.jval.2011.04.002 (2011).

6 Sutton, A. J. & Higgins, J. P. Recent developments in meta-analysis. *Stat Med* **27**, 625-650, doi:10.1002/sim.2934 (2008).

7 Smolen, J. S. *et al.* EULAR recommendations for the management of rheumatoid arthritis with synthetic and biological disease-modifying antirheumatic drugs: 2019 update. *Ann Rheum Dis* **79**, 685-699, doi:10.1136/annrheumdis-2019-216655 (2020).

8 Kay, J. *et al.* Consensus-based recommendations for the use of biosimilars to treat rheumatological diseases. *Ann Rheum Dis* **77**, 165-174, doi:10.1136/annrheumdis-2017-211937 (2018).

9 Allocati, E., Bertele, V., Gerardi, C., Garattini, S. & Banzi, R. Clinical evidence supporting the marketing authorization of biosimilars in Europe. *Eur J Clin Pharmacol* **76**, 557-566, doi:10.1007/s00228-019-02805-y (2020).

10 Treadwell JR *et al.* in *(Prepared by the EPC Workgroup under Contract No. 290-2007-10063.)* Vol. 65 (ed Methods Research Report) 1144-1149 (Agency for Healthcare Research and Quality, Agency for Healthcare Research and Quality, 2012).

11 World Health Organization. Guidelines on evaluation of similar biotherapeutic products (SBPs). (World Health Organization, Geneva, Switzerland, 2009).

12 Felson, D. T. *et al.* American College of Rheumatology. Preliminary definition of improvement in rheumatoid arthritis. *Arthritis Rheum* **38**, 727-735, doi:10.1002/art.1780380602 (1995).

13 Bruce, B. & Fries, J. F. The Stanford Health Assessment Questionnaire: a review of its history, issues, progress, and documentation. *J Rheumatol* **30**, 167-178 (2003).

14 Horta-Baas, G. Patient-Reported Outcomes in Rheumatoid Arthritis: A Key Consideration for Evaluating Biosimilar Uptake? *Patient Relat Outcome Meas* **13**, 79-95, doi:10.2147/PROM.S256715 (2022).

15 Gossec, L., Dougados, M. & Dixon, W. Patient-reported outcomes as end points in clinical trials in rheumatoid arthritis. *RMD Open* **1**, e000019, doi:10.1136/rmdopen-2014-000019 (2015).

16 Kluzek, S., Dean, B. & Wartolowska, K. A. Patient-reported outcome measures (PROMs) as proof of treatment efficacy. *BMJ Evid Based Med* **27**, 153-155, doi:10.1136/bmjebm-2020-111573 (2022).

17 Moots, R. *et al.* Switching Between Reference Biologics and Biosimilars for the Treatment of Rheumatology, Gastroenterology, and Dermatology Inflammatory Conditions: Considerations for the Clinician. *Curr Rheumatol Rep* **19**, 37, doi:10.1007/s11926-017-0658-4 (2017).

18 McGuinness, L. A. & Higgins, J. P. T. Risk-of-bias VISualization (robvis): An R package and Shiny web app for visualizing risk-of-bias assessments. *Res Synth Methods* **12**, 55-61, doi:10.1002/jrsm.1411 (2021).

19 Kwon, D. & Reis, I. M. Simulation-based estimation of mean and standard deviation for meta-analysis via Approximate Bayesian Computation (ABC). *BMC Med Res Methodol* **15**, 61, doi:10.1186/s12874-015-0055-5 (2015).

20 Puhan, M. A. *et al.* A GRADE Working Group approach for rating the quality of treatment effect estimates from network meta-analysis. *BMJ* **349**, g5630, doi:10.1136/bmj.g5630 (2014).

21 Brignardello-Petersen, R. *et al.* Advances in the GRADE approach to rate the certainty in estimates from a network meta-analysis. *J Clin Epidemiol* **93**, 36-44, doi:10.1016/j.jclinepi.2017.10.005 (2018).

22 Brignardello-Petersen, R. *et al.* GRADE approach to rate the certainty from a network meta-analysis: avoiding spurious judgments of imprecision in sparse networks. *J Clin Epidemiol* **105**, 60-67, doi:10.1016/j.jclinepi.2018.08.022 (2019).

23 Ascef BO *et al.* (2022).

24 Alten, R., Glover, J., Matsunaga, N., Chisholm, D. & Genovese, M. in *Oral Presentations* 59-59 (Ann Rheum Dis, 2017).

25 Genovese MC, Glover J, Matsunaga N, Chisholm D & Alten R. in *2017 ACR ARHP Annual Meeting* Vol. 69 (ed Wiley) (Arthritis Reumatol, NJ USA, 2017).

26 Alten, R., Genovese, M. C., Muniz, R. & Kellner, H. 1135.1131-1135 (Ann Rheum Dis, 2019).

27 Alten R *et al.* Vol. 13 S320-S320 (Journal of Crohn's and Colitis, 2019).

28 Alten, R. *et al.* Immunogenicity of an adalimumab biosimilar, FKB327, and its reference product in patients with rheumatoid arthritis. *Int J Rheum Dis* **23**, 1514-1525, doi:10.1111/1756-185X.13951 (2020).

29 Genovese, M. C. *et al.* FKB327, an adalimumab biosimilar, versus the reference product: results of a randomized, Phase III, double-blind study, and its open-label extension. *Arthritis Res Ther* **21**, 281, doi:10.1186/s13075-019-2046-0 (2019).

30 Genovese, M. C., Kellner, H., Arai, Y., Muniz, R. & Alten, R. Long-term safety, immunogenicity and efficacy comparing FKB327 with the adalimumab reference product in patients with active rheumatoid arthritis: data from randomised double-blind and open-label extension studies. *RMD Open* **6**, doi:10.1136/rmdopen-2019-000987 (2020).

31 Cohen, S. *et al.* Efficacy and safety of the biosimilar ABP 501 compared with adalimumab in patients with moderate to severe rheumatoid arthritis: a randomised, double-blind, phase III equivalence study. *Ann Rheum Dis* **76**, 1679-1687, doi:10.1136/annrheumdis-2016-210459 (2017).

32 Cohen, S. *et al.* An open-label extension study to demonstrate long-term safety and efficacy of ABP 501 in patients with rheumatoid arthritis. *Arthritis Res Ther* **21**, 84, doi:10.1186/s13075-019-1857-3 (2019).

33 Fleischmann, R. M. *et al.* A comparative clinical study of PF-06410293, a candidate adalimumab biosimilar, and adalimumab reference product (Humira(R)) in the treatment of active rheumatoid arthritis. *Arthritis Res Ther* **20**, 178, doi:10.1186/s13075-018-1676-y (2018).

34 Fleischmann R *et al.* in *ACR Convergence 2020* Vol. 72 (Arthritis Rheumatol, Berlin, Germany, 2020).

35 Fleischmann, R. M. *et al.* Randomised study of PF-06410293, an adalimumab (ADL) biosimilar, compared with reference ADL for the treatment of active rheumatoid arthritis: results from weeks 26-52, including a treatment switch from reference ADL to PF-06410293. *RMD Open* **7**, doi:10.1136/rmdopen-2021-001578 (2021).

36 Cohen, S. B. *et al.* Similar efficacy, safety and immunogenicity of adalimumab biosimilar BI 695501 and Humira reference product in patients with moderately to severely active rheumatoid arthritis: results from the phase III randomised VOLTAIRE-RA equivalence study. *Ann Rheum Dis* **77**, 914-921, doi:10.1136/annrheumdis-2017-212245 (2018).

37 Cohen, S. B. *et al.* Long-term safety, efficacy, and immunogenicity of adalimumab biosimilar BI 695501 and adalimumab reference product in patients with moderately-to-severely active rheumatoid arthritis: results from a phase 3b extension study (VOLTAIRE-RAext). *Expert Opin Biol Ther* **19**, 1097-1105, doi:10.1080/14712598.2019.1645114 (2019).

38 Weinblatt, M. E. *et al.* Switching From Reference Adalimumab to SB5 (Adalimumab Biosimilar) in Patients With Rheumatoid Arthritis: Fifty-Two-Week Phase III Randomized Study Results. *Arthritis Rheumatol* **70**, 832-840, doi:10.1002/art.40444 (2018).

39 Weinblatt, M. E. *et al.* Phase III Randomized Study of SB5, an Adalimumab Biosimilar, Versus Reference Adalimumab in Patients With Moderate-to-Severe Rheumatoid Arthritis. *Arthritis Rheumatol* **70**, 40-48, doi:10.1002/art.40336 (2018).

40 Wiland, P. *et al.* in *Poster Presentations* Vol. 70 706.702-707 (Arthritis Rheumatol 2019).

41 Wiland, P. *et al.* Switching to Biosimilar SDZ-ADL in Patients with Moderate-to-Severe Active Rheumatoid Arthritis: 48-Week Efficacy, Safety and Immunogenicity Results From the Phase III, Randomized, Double-Blind ADMYRA Study. *BioDrugs* **34**, 809-823, doi:10.1007/s40259-020-00447-6 (2020).

42 Kay, J. *et al.* Efficacy and safety of biosimilar CT-P17 versus reference adalimumab in subjects with rheumatoid arthritis: 24-week results from a randomized study. *Arthritis Res Ther* **23**, 51, doi:10.1186/s13075-020-02394-7 (2021).

43 Furst DE *et al.* Efficacy and safety of switching from reference adalimumab to CT-P17 (100 mg/ml): 52-week randomized, double-blind study in rheumatoid arthritis. *Rheumatology* **0**, 1–11, doi:10.1093/rheumatology/keab46017 (2021).

44 Emery, P. *et al.* A phase III randomised, double-blind, parallel-group study comparing SB4 with etanercept reference product in patients with active rheumatoid arthritis despite methotrexate therapy. *Ann Rheum Dis* **76**, 51-57, doi:10.1136/annrheumdis-2015-207588 (2015).

45 Emery, P. *et al.* 52-week results of the phase 3 randomized study comparing SB4 with reference etanercept in patients with active rheumatoid arthritis. *Rheumatology (Oxford)* **56**, 2093-2101, doi:10.1093/rheumatology/kex269 (2017).

46 Emery, P. *et al.* Long-term efficacy and safety in patients with rheumatoid arthritis continuing on SB4 or switching from reference etanercept to SB4. *Ann Rheum Dis*, doi:10.1136/annrheumdis-2017-211591 (2017).

47 O'Dell J *et al.* Vol. 75 143-143 (Annals of the Rheumatic Diseases 2016).

48 O'Dell, J. *et al.* in *Poster Presentations* Vol. 76 831.832-831 (Ann Rheum Dis, 2017).

49 Matsuno, H. *et al.* Phase III, multicentre, double-blind, randomised, parallel-group study to evaluate the similarities between LBEC0101 and etanercept reference product in terms of efficacy and safety in patients with active rheumatoid arthritis inadequately responding to methotrexate. *Ann Rheum Dis* **77**, 488-494, doi:10.1136/annrheumdis-2017-212172 (2017).

50 Park, M. C. *et al.* Long-term efficacy, safety and immunogenicity in patients with rheumatoid arthritis continuing on an etanercept biosimilar (LBEC0101) or switching from reference etanercept to LBEC0101: an open-label extension of a phase III multicentre, randomised, double-blind, parallel-group study. *Arthritis Res Ther* **21**, 122, doi:10.1186/s13075-019-1910-2 (2019).

51 Matucci-Cerinic, M. *et al.* Efficacy, safety and immunogenicity of GP2015, an etanercept biosimilar, compared with the reference etanercept in patients with moderate-to-severe rheumatoid arthritis: 24-week results from the comparative phase III, randomised, double-blind EQUIRA study. *RMD Open* **4**, e000757, doi:10.1136/rmdopen-2018-000757 (2018).

52 Jaworski, J. *et al.* Switch from reference etanercept to SDZ ETN, an etanercept biosimilar, does not impact efficacy, safety, and immunogenicity of etanercept in patients with moderate-to-severe rheumatoid arthritis: 48-week results from the phase III, randomized, double-blind EQUIRA study. *Arthritis Res Ther* **21**, 130, doi:10.1186/s13075-019-1907-x (2019).

53 Yamanaka, H. *et al.* A Comparative Study to Assess the Efficacy, Safety, and Immunogenicity of YLB113 and the Etanercept Reference Product for the Treatment of Patients with Rheumatoid Arthritis. *Rheumatol Ther* **7**, 149-163, doi:10.1007/s40744-019-00186-3 (2020).

54 Yoo, D. H. *et al.* A randomised, double-blind, parallel-group study to demonstrate equivalence in efficacy and safety of CT-P13 compared with innovator infliximab when coadministered with methotrexate in patients with active rheumatoid arthritis: the PLANETRA study. *Ann Rheum Dis* **72**, 1613-1620, doi:10.1136/annrheumdis-2012-203090 (2013).

55 Yoo, D. H. *et al.* A phase III randomized study to evaluate the efficacy and safety of CT-P13 compared with reference infliximab in patients with active rheumatoid arthritis: 54-week results from the PLANETRA study. *Arthritis Res Ther* **18**, 82, doi:10.1186/s13075-016-0981-6 (2016).

56 Yoo, D. H. *et al.* Efficacy and safety of CT-P13 (biosimilar infliximab) in patients with rheumatoid arthritis: comparison between switching from reference infliximab to CT-P13 and continuing CT-P13 in the PLANETRA extension study. *Ann Rheum Dis* **76**, 355-363, doi:10.1136/annrheumdis-2015-208786 (2017).

57 Kay, J. *et al.* in *Annals of the Rheumatic Diseases* Vol. 73 64.61-64 (2014).

58 Taylor, P., Wyand, M., Knight, A., Costantino, C. & Lassen, C. in *Annals of the Rheumatic Diseases* Vol. 75 488.482-489 (2016).

59 Choe, J. Y. *et al.* A randomised, double-blind, phase III study comparing SB2, an infliximab biosimilar, to the infliximab reference product Remicade in patients with moderate to severe rheumatoid arthritis despite methotrexate therapy. *Ann Rheum Dis* **76**, 58-64, doi:10.1136/annrheumdis-2015-207764 (2017).

60 Smolen, J. S. *et al.* Comparing biosimilar SB2 with reference infliximab after 54 weeks of a double-blind trial: clinical, structural and safety results. *Rheumatology (Oxford)* **56**, 1771-1779, doi:10.1093/rheumatology/kex254 (2017).

61 Smolen, J. S. *et al.* Safety, immunogenicity and efficacy after switching from reference infliximab to biosimilar SB2 compared with continuing reference infliximab and SB2 in patients with rheumatoid arthritis: results of a randomised, double-blind, phase III transition study. *Ann Rheum Dis* **77**, 234-240, doi:10.1136/annrheumdis-2017-211741 (2018).

62 Matsuno, H. & Matsubara, T. A randomized double-blind parallel-group phase III study to compare the efficacy and safety of NI-071 and infliximab reference product in Japanese patients with active rheumatoid arthritis refractory to methotrexate. *Mod Rheumatol* **29**, 919-927, doi:10.1080/14397595.2018.1533063 (2018).

63 Genovese, M. C. *et al.* Comparative clinical efficacy and safety of the proposed biosimilar ABP 710 with infliximab reference product in patients with rheumatoid arthritis. *Arthritis Res Ther* **22**, 60, doi:10.1186/s13075-020-2142-1 (2020).

64 Jani, R. H. *et al.* A prospective, randomized, double-blind, multicentre, parallel-group, active controlled study to compare efficacy and safety of biosimilar adalimumab (Exemptia; ZRC-3197) and adalimumab (Humira) in patients with rheumatoid arthritis. *Int J Rheum Dis* **19**, 1157-1168, doi:10.1111/1756-185X.12711 (2015).

65 Takeuchi, T. *et al.* Evaluation of the pharmacokinetic equivalence and 54-week efficacy and safety of CT-P13 and innovator infliximab in Japanese patients with rheumatoid arthritis. *Mod Rheumatol* **25**, 817-824, doi:10.3109/14397595.2015.1022297 (2015).

66 Bae, S. C. *et al.* A phase III, multicentre, randomised, double-blind, active-controlled, parallel-group trial comparing safety and efficacy of HD203, with innovator etanercept, in combination with methotrexate, in patients with rheumatoid arthritis: the HERA study. *Ann Rheum Dis* **76**, 65-71, doi:10.1136/annrheumdis-2015-207613 (2016).

67 Lila, A. M. *et al.* A phase III study of BCD-055 compared with innovator infliximab in patients with active rheumatoid arthritis: 54-week results from the LIRA study. *Rheumatol Int* **39**, 1537-1546, doi:10.1007/s00296-019-04359-9 (2019).

68 Edwards, C. J. *et al.* Safety of adalimumab biosimilar MSB11022 (acetate-buffered formulation) in patients with moderately-to-severely active rheumatoid arthritis. *Clin Rheumatol* **38**, 3381-3390, doi:10.1007/s10067-019-04679-y (2019).

69 Jamshidi, A. *et al.* A phase III, randomized, two-armed, double-blind, parallel, active controlled, and non-inferiority clinical trial to compare efficacy and safety of biosimilar adalimumab (CinnoRA(R)) to the reference product (Humira(R)) in patients with active rheumatoid arthritis. *Arthritis Res Ther* **19**, 168, doi:10.1186/s13075-017-1371-4 (2017).

70 Strusberg, I. *et al.* Efficacy, Safety, and Immunogenicity of Biosimilar Etanercept (Enerceptan) Versus Its Original Form in Combination With Methotrexate in Patients With Rheumatoid Arthritis: A Randomized, Multicenter, Evaluator-Blinded, Noninferiority Study. *J Clin Rheumatol* **27**, S173-S179, doi:10.1097/RHU.0000000000001616 (2021).

71 Matsuno H *et al.* (Clinical and Experimental Rheumatology, 2021).
